# Porous Carbon-Based Supercapacitors Directly Derived from Metal–Organic Frameworks

**DOI:** 10.3390/ma13184215

**Published:** 2020-09-22

**Authors:** Hyun-Chul Kim, Seong Huh

**Affiliations:** Department of Chemistry and Protein Research Center for Bio-Industry, Hankuk University of Foreign Studies, Yongin 17035, Korea; mdkop@naver.com

**Keywords:** Metal–Organic Frameworks (MOFs), carbonization, pyrolysis, porous carbons, supercapacitors, ultracapacitors, symmetric supercapacitors, asymmetric supercapacitors, pseudocapacitors, hybrid electrochemical capacitors, heteroatom-doped carbons, carbon composites, capacitance

## Abstract

Numerously different porous carbons have been prepared and used in a wide range of practical applications. Porous carbons are also ideal electrode materials for efficient energy storage devices due to their large surface areas, capacious pore spaces, and superior chemical stability compared to other porous materials. Not only the electrical double-layer capacitance (EDLC)-based charge storage but also the pseudocapacitance driven by various dopants in the carbon matrix plays a significant role in enhancing the electrochemical supercapacitive performance of porous carbons. Since the electrochemical capacitive activities are primarily based on EDLC and further enhanced by pseudocapacitance, high-surface carbons are desirable for these applications. The porosity of carbons plays a crucial role in enhancing the performance as well. We have recently witnessed that metal–organic frameworks (MOFs) could be very effective self-sacrificing templates, or precursors, for new high-surface carbons for supercapacitors, or ultracapacitors. Many MOFs can be self-sacrificing precursors for carbonaceous porous materials in a simple yet effective direct carbonization to produce porous carbons. The constituent metal ions can be either completely removed during the carbonization or transformed into valuable redox-active centers for additional faradaic reactions to enhance the electrochemical performance of carbon electrodes. Some heteroatoms of the bridging ligands and solvate molecules can be easily incorporated into carbon matrices to generate heteroatom-doped carbons with pseudocapacitive behavior and good surface wettability. We categorized these MOF-derived porous carbons into three main types: (i) pure and heteroatom-doped carbons, (ii) metallic nanoparticle-containing carbons, and (iii) carbon-based composites with other carbon-based materials or redox-active metal species. Based on these cases summarized in this review, new MOF-derived porous carbons with much enhanced capacitive performance and stability will be envisioned.

## 1. Introduction

The development of efficient electrochemical energy storage materials is an urgent issue for modern scientific society [1,2]. Many electricity-driven devices ranging from small portable electronics to electric vehicles (EVs) absolutely require high performance rechargeable energy storage devices as a primary component [3]. In this sense, high-capacity lithium-ion batteries (LIBs) have been being studied intensively for better performance [4,5]. The impact of LIBs on modern technology is enormous. Meanwhile, there is a growing interest in supercapacitors, or ultracapacitors, as a means of alternative energy storage devices with high power [6]. Generally, LIBs outperform supercapacitors due to their high energy density. Nevertheless, chemically stable supercapacitors are especially useful for various cutting-edge applications [7]. Furthermore, fabrication of supercapacitors is simple and their raw materials are usually cost-effective. In this regard, research towards high performance supercapacitors is continuously growing.

**Scheme 1:**
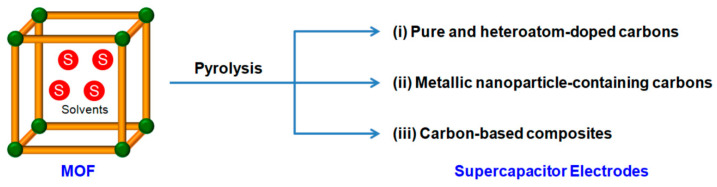
A schematic illustration of the three main types of porous carbons directly derived from MOFs, and which can be used as supercapacitor electrode materials.

**Scheme 2:**
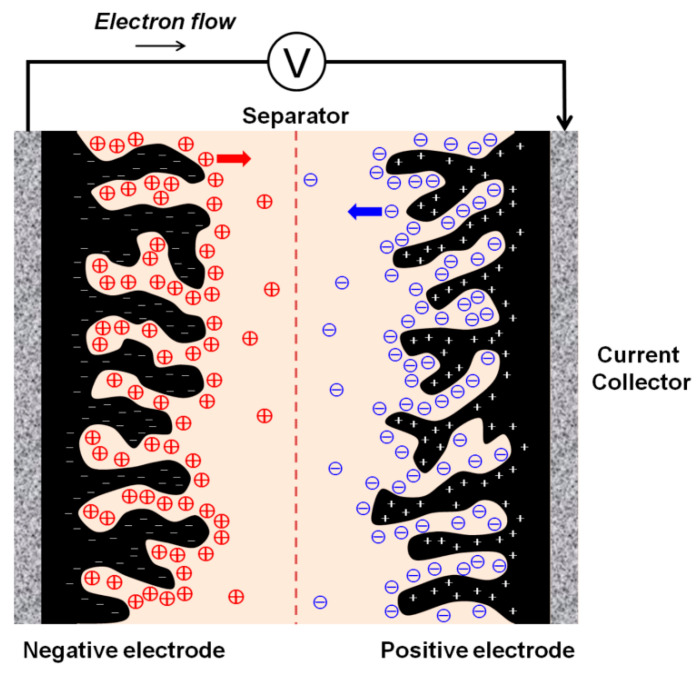
A simplified view of the porous carbon-based supercapacitor to show operating principle of energy storage.

There are previous excellent review articles well covering supercapacitors [6,7,8,9,10,11,12,13]. Therefore, we mostly focus on the supercapacitors directly derived from various metal–organic frameworks (MOFs). Most MOFs are made of two key components, metal ions and bridging organic-based linkers [14,15,16]. In some cases, solvate molecules or counterions can be present. Therefore, it is possible to obtain carbon-based materials through simple pyrolysis of MOFs under inert atmosphere. The numerously different types of organic-based bridging linkers can act as very good carbon precursors. Since the properties of carbon materials are mainly related to their precursors, one can prepare many different types of carbons by choosing different MOFs. MOF-derived supercapacitor electrode materials can be classified into three main types based on their chemical compositions of the resulting porous carbons upon pyrolysis of MOFs under inert atmosphere: (i) pure and heteroatom-doped carbons, (ii) metallic nanoparticle (NP)-containing carbons, and (iii) other carbon-based composites as illustrated in Scheme 1. Simple pyrolysis process of certain MOFs affords almost pure carbonaceous materials with good porosity. In this case, volatile metallic elements are effectively lost during high-temperature pyrolysis conditions [17]. Contrarily, some heteroatoms such as N, S, O, and P can be incorporated into the carbon matrix. These heteroatoms are originated from the organic-based linkers or counterions [18,19,20,21,22,23]. Incomplete removal of metal ions often produces metallic NP-containing carbons and carbon-based composite materials [24,25]. All these porous materials are good candidates for supercapacitor applications under various working conditions. Pure carbonaceous materials can behave as supercapacitors mainly based on electrical double-layer capacitance (EDLC). The charge can be stored in a sandwich-type porous carbon-based electrode is separated by a porous membrane (Scheme 2). When the voltage is applied to two porous carbon electrodes, charges are stored mostly on the surfaces of the electrodes through the formation of EDL. Not only the types of metal ions but also the types of bridging ligands may play a crucial role in determining the structures of MOFs. Generally, the bridging ligands with small physical dimensions tend to form MOFs with low porosity. Carbons prepared from these MOFs are likely to show less developed porosity with decreased capacitive performance. Additional pseudocapacitances apply for both heteroatom-doped carbons and carbon composites due to the redox-active components. Now that there is promising progress using these materials as supercapacitor electrodes, we would like to review this interesting area for electrochemical energy storage.

The performances of supercapacitors are evaluated by several key parameters [26]: gravimetric specific capacitance (*C*, F g^−1^), specific energy (*E*, W h kg^−1^), specific power (*P*, W kg^−1^), and charge/discharge recyclability. The relevant basic equations are shown below:

$$C=\frac{I\Delta t}{m\Delta V}\;E=\frac{1}{7.2}C{\left(\Delta V\right)}^{2}\;P=3600\frac{E}{\Delta t}$$

$$E=\frac{1}{7.2}C{\left(\Delta V\right)}^{2}$$

$$P=3600\frac{E}{\Delta t}$$

where, *I*: charge/discharge current in A, Δ*t*: discharge time in s, *m*: mass of the active electrode material in g, Δ*V*: voltage window in V.

## 2. Pure and Heteroatom-Doped Carbons

### 2.1. Zn-MOF-Derived Carbons

The most well investigated MOF precursors for porous carbons are Zn-based MOF systems. Zn-MOFs can be easily assembled from divalent Zn*^2+^* ion and various carboxylate-based bridging ligands. There are fairly many reports relevant this case as summarized in Table 1. The volatile Zn species tend to be simultaneously removed during high-temperature carbonization. Therefore, we analyze this system first.

#### 2.1.1. MOF-5-Derived Carbons

Xu et al. reported the unprecedented example of MOF-derived nanoporous carbon (NPC) as an electrode material for EDLC capacitor [27]. A furfuryl alcohol (FA) vapor as a carbon source was infiltrated into the pores of MOF-5 ([Zn_4_O(bdc)_3_], bdc = 1,4-benzenedicarboxylate) template. Polymerization of the infiltrated FA was performed to construct PFA/MOF-5 composite (PFA = poly(furfuryl alcohol)), and this composite was transformed into the NPC via one-step carbonization at 1000 °C (8 h, Ar flow). Without any additional purification process, the resulting NPC contained ultra-high Brunauer–Emmett–Teller surface area (S_BET_) as well as large total pore volume (V_total_ = 2.06 cm^3^ g^−1^). The NPC with hierarchical porosity had good electrochemical performance for symmetric EDLC-type supercapacitor electrode operating in 1 M H_2_SO_4_ electrolyte. It showed a good gravimetric capacitance of 312 F g^−1^ at a scan rate of 1 mV s^−1^ and 159 F g^−1^ at 50 mV s^−1^. The same group applied incipient wetness method to insert FA into MOF-5 for the synthesis of NPCs [28]. The as-prepared NPCs (as-NPC_530_, as-NPC_650_, as-NPC_800_, NPC_900_ and NPC_1000_) were prepared from the carbonization (8 h, Ar flow) of the PFA/MOF-5 composite. Three NPCs (NPC_530_, NPC_650_ and NPC_800_) were generated by HCl (0.1 M) treatment of the as-prepared NPCs. Carbonization temperature-dependent properties of the nanoporous carbons (NPCs) such as S_BET_, pore size distribution, and electrical conductivity had great influence on the electrochemical performance of symmetric NPC supercapacitor at 1 M H_2_SO_4_ electrolyte. Because of their fine mesoporosities combined with superior electric conductivity, the NPC electrodes except for NPC_530_ showed ideal capacitive behavior as well as good rate capability.

MOF-5 as a single-carbon source or MOF-5 with other secondary carbon sources (phenolic resin or carbon tetrachloride and ethylenediamine) was employed to prepare MOF-5-derived carbons [29]. Without or with secondary carbon precursors, three kinds of porous carbons (MC, MPC and MAC) were synthesized from MOF-5 at 900 °C (N_2_ flow). Subsequent potassium hydroxide (KOH) activation (700 °C, 3 h, the mass ratio of KOH/carbon = 5/1) of each carbon generated corresponding three activated samples (MC-A, MPC-A, and MAC-A). Electrochemical performances of the obtained carbon electrodes were investigated under both 6 M KOH electrolytic three-electrode (3E) system and 1.5 M NEt_4_BF_4_ electrolytic two-electrode (2E) system in acetonitrile. The maximum gravimetric energy densities of 9.4 and 31.2 W h kg^−1^ could be obtained for MAC-A in 6 M KOH and 1.5 M NEt_4_BF_4_/AN electrolytes, respectively. Because of high packing density of 0.93 g cm^−3^, MAC-A also delivered high volumetric energy density of 8.8 W h L^−1^ at aqueous electrolyte and 29.0 W h L^−1^ at organic electrolyte. Facile ionic transport due to hierarchical porosity also led to superior energy density rate capabilities for MC, MC-A, MPC, and MPC-A. For MAC-A at a discharge time of 10 s, the largest volumetric energy density and power density of 6.6 W h L^−1^ and 2.4 kW L^−1^ were shown in an aqueous electrolyte.

Glucose was employed as a secondary carbon source for the MOF-5-based three-dimensional (3D) hierarchical porous carbon [30]. A green and effective synthetic method of the 3D hierarchical porous carbon from MOF-5/glucose composite was accomplished via solvothermal reaction and a single-step carbonization. Primary construction of 3D structure of MOF-5 sacrificial template and subsequent infiltration and polymerization of glucose carbon source were performed at solvothermal condition (160 °C). Direct carbonization of optimally synthesized composite (solvothermal reaction time = 25 h) at 950 °C (4.5 h) could form 3D coral-like porous carbon with hierarchical porosity. As an active electrode material for a symmetric supercapacitor (SSC) in 1 M NEt_4_BF_4_/propylene carbonate electrolyte, this carbon exhibited capacitances of 175 F g^−1^ at 0.6 A g^−1^ and 165 F g^−1^ at 12 A g^−1^. These good electrochemical results were assignable to its micro-/meso-/macroporous hierarchical structure. The roles of macropores, mesopores, and micropores are efficient ion-buffering storage, generation of ionic transport pathway, and providing ionic diffusion space, respectively. Another 3D hierarchically porous carbon with controlled large-scale meso-/macroporosity was synthesized from a facile solvent evaporation strategy during the carbonization of MOF-5 without the requirement of templating and replication process (900 °C, 3 h, N_2_ flow) [31]. A direct pyrolytic evaporation of non-volatile *N*,*N*-dimethylformamide (DMF) solvent molecules captured in MOF-5 micropores was performed to reorganize the MOF template for the construction of ultra-highly porous MOF-derived carbon, MDC-D. The resulting MDC-D exhibited an interconnected macropore structure with 3D wormhole-like shape, high S_BET_, hierarchical porosity, and exceptionally high V_total_ of 5.45 cm^3^ g^−1^. The electrochemical function of micropores was thought to be an ionic reservoir, and the mesopores and macropores were considered to be pathways of ionic transport. As a result, the MDC-D working electrode of 3E system operating in 1 M NaCl showed high rate capacities in CV and electrochemical impedance spectroscopy (EIS).

MOF-5-derived 3D interconnected porous carbons (IPCs) were also synthesized through sonochemical KOH activation (600 W, 20 min) combined with acid washing (2 M HCl) [32]. The obtained carbon samples known as IPC_x-M_ (x: the mass ratio of MOF-5/KOH, M: microwave heating) showed a unique structure and better electrochemical performance compared to the control sample of IPC_2-C_ (C: conventional heating at 850 °C). Very thin wall structure provided short pathway for fast ionic transport. Ion accessible porosity was also beneficial for ionic storage. The interconnected framework improved electron conduction. Among them, IPC_3-M_ was the best electrode material for SSC in 6 M KOH electrolyte. Mesoporous carbons derived from MOF-5-based carbon composites were reported [33,34]. A soft templating/carbonization strategy was applied to the synthesis of carbon nanospheres (CNSs) from MOF-5/AC composite (AC stands for activated carbon) [33]. AC was used as a nucleation mediator for the crystallization of MOF-5 at mild room temperature. Thus, AC acted as a soft template for the preparation of MOF-5/AC. Subsequently, MOF-5/AC-C nsp 850 was obtained via carbonization of the composite at 850 °C (6 h, Ar flow) and subsequent 1 M hydrofluoric acid (HF) treatment. Compared to the fragmented porous carbon (MOF-5-C 850) directly derived from the as-prepared MOF-5, the obtained MOF-5/AC-C nsp 850 exhibited well-defined and spherical particle morphology with improved mechanical strength. In addition, it also possessed better porosity than the MOF-5-C 850. Therefore, enhanced electrochemical capacitive performance for the 3E system with 6 M KOH could be seen in the MOF-5/AC-C nsp 850 electrode.

**Figure 1:**
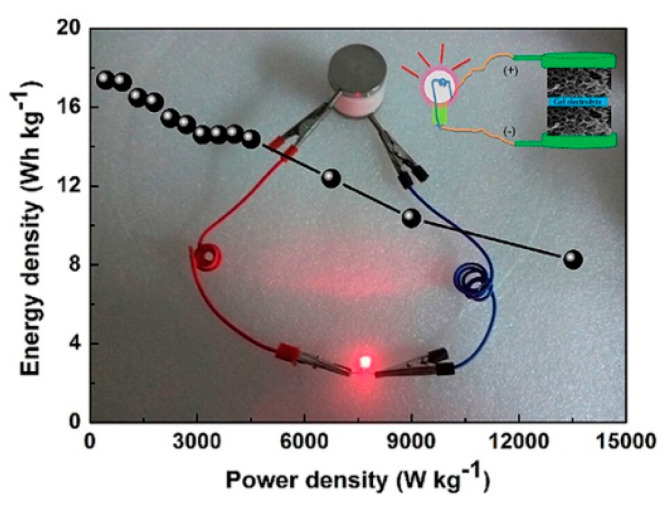
A Ragone plot and schematic descriptions of the all-solid-phase SSC assembled from MOF-5 derived porous carbon electrode with Na_2_SO_4_-PVA gel electrolyte [35].

The KOH activation combined with one-step carbonization of MOF-5/coal tar pitch composites was introduced to simply fabricate the interconnected mesoporous carbon sheets (IMCSs) [34]. KOH-mixed KOH/coal tar pitch composites were prepared from cheap high yielded carbon precursor, i.e., coal tar pitch, and room temperature synthesized MOF-5 as both a template and additional carbon precursor. KOH-mixed KOH/coal tar pitch composites were carbonized at 850 °C (1 h, N_2_ flow) and then neutralized with 2 M HCl to form IMCS_x-y-z_ (mass of coal tar pitch (x), MOF-5 (y), and KOH (z)). Among the samples, the IMCS_4-8-6_-based carbon electrode showed superior SSC performance in 6 M KOH electrolyte. Highest specific capacitance of the IMCS_4-8-6_ at 0.05 A g^−1^ was 242 F g^−1^, and it retained to 80.2% (194 F g^−1^) up to 20 A g^−1^. MOF-5-derived electrode for symmetric all-solid-phase supercapacitor was reported [35]. Porous carbon materials with high purity as well as an ultra-large S_BET_ was synthesized from single-step carbonization of MOF-5 precursor at 900 °C (2 h, N_2_ flow). The amorphous porous carbon showed rich porosity with hierarchical pore size distribution (average D_pore_ = ~7 nm). Under 3E system operating in 0.5 M Na_2_SO_4_ electrolyte, the as-made porous carbon electrode yielded maximum specific capacitance of 148.8 F g^−1^ at 5 A g^−1^ and high rate capacity (88.6% retention) at 50 A g^−1^. With the extension of operating voltage up to 1.8 V, the carbon-based electrode was also applied to the symmetric all-solid-phase supercapacitor acting in Na_2_SO_4_/PVA (PVA = polyvinyl alcohol) gel electrolyte. At 1 A g^−1^, the SSC delivered a high energy density of 17.37 W h kg^−1^ with a power density of 449.9 W kg^−1^. This was a rare example of MOF-derived carbon employed as electrode material for solid-state supercapacitor (Figure 1).

The MOF-5-derived carbon (CMOF-5) electrode with potential environmental and industrial applicabilities was reported [36]. First, the economical and structurally effective (free from void/cracking) PVDF/CMOF-5 nanocomposite coating on the current collector was easily achieved via evaporation of acetone suspension containing poly (vinylidene fluoride) (PVDF) and CMOF-5. The CMOF-5 was synthesized from simple carbonization of MOF-5 at 1000 °C (1 h, Ar flow) and subsequent HCl treatment. After successive ultra-sonification, dropping, and evaporation, there was an adequate contact of CMOF-5 with the PVDF binder in the nanocomposite. The solvent evaporated PVDF/CMOF-5 composite held thinner thickness and more stable architecture in comparison with PVDF/CMOF-5 composite prepared by high-pressure pressing. The PVDF/CMOF-5 composite prepared by evaporation also exhibited better porosity than PVDF/CMOF-5 composite made from compressing. Therefore, when applied as a working electrode for SSC in 1 M H_2_SO_4_ electrolyte, the evaporation induced CMOF-5 electrode showed high specific capacitance of 138 F g^−1^ at 3 A g^−1^ and a good rate capacity. Interestingly, the CMOF-5 could be prepared from the recycled raw materials. Using distilled waste DMF solvent and hydrothermally depolymerized polyethylene terephthalate (PET)-based terephthalic acid, or 1,4-benzenedicarboxylic acid, the obtained r1-CMOF-5 possessed good electrochemical performance. Finally, the CMOF-5 was a reusable working electrode fabricated from spent electrodes. Acetone washing of the previously used electrode and following HCl washing, the CMOF-5 (r2-CMOF-5) was effectively recovered.

#### 2.1.2. Other Zn-MOF-Derived Carbons

There are two reports about carbon electrodes derived from nonporous Zn-MOF [37,38]. A hollow porous carbon (HPC) with laminated morphology was prepared from a nonporous metal–organic coordinate polymer (MOCP) self-sacrificing template and phenol resin polymer as a secondary carbon source [37]. After the carbonization of MOCP/phenol resin polymer composite at 850 °C (3 h, N_2_ flow), the obtained HPC showed high S_BET_ and laminated nanostructure containing both micropores and mesopores. The enhancement of ionic diffusion and electrochemically effective surface area could be attributable to this hierarchical porosity. Therefore, the carbon material for EDLC in 3E system in 6 M KOH electrolyte yielded a maximum capacitance of 234 F g^−1^ at 0.01 A g^−1^ with high capacitive retention. A series of porous carbon materials have been synthesized through simple direct carbonization of as-prepared nonporous Zn-MOFs at 1000 °C (3 h, Ar flow) except for MOF-5 with S_BET_ = 2385 m^2^ g^−1^ [38]. The self-sacrificing templates were MOF-5 [Zn_4_O(bdc)_3_], MOF-2 [(H_2_NEt)_2_[Zn_3_(bdc)_4_]∙3DEF], Zn-btc [Zn_3_(btc)_2(_DMSO)_4_], Zn-ndc [Zn(ndc)(H_2_O)], and Zn-paa [Zn_2_(paa)_2_]. In addition, the corresponding carbons were C-MOF-5, C-MOF-2, C-Zn-btc (btc = 1,3,5-benzenetricarboxylic acid), C-Zn-ndc (ndc = 2,6-naphthalenedicarboxylic acid), C-Zn-paa (paa = 1,4-phenylenediacetic acid), and C-Zn-ada (ada = 1,3-adamantanediacetic acid). Systematic studies about the influence of linker nature upon the resultant carbons’ properties were performed by variation of linkers in the MOF templates. The linkers contained flexible alicyclic or rigid aromatic backbones. The morphology of the resulting carbons was dependent on the flexibility of linkers. The rigid aromatic linker containing MOF-based carbons (C-MOF-5, C-MOF-2, C-Zn-ndc, and C-Zn-btc) showed well-defined and periodically arranged carbon sheets. However, the flexible alicyclic linker containing MOF-based carbons (C-Zn-paa and C-Zn-ada) had less-ordered and 2D carbon sheets. The linker flexibility also affected the porosity of the obtained carbons. The carbons derived from the rigid linker containing MOFs possessed improved S_BET_ (C-MOF-5, C-MOF-2, C-Zn-btc, and C-Zn-ndc are 2184, 1378, 1326, and 920 m^2^ g^−1^, respectively) compared with flexible linker possessing MOF-derived ones (C-Zn-paa and C-Zn-ada were 495 and 513 m^2^ g^−1^, respectively). In addition, the linear correlation between S_BET_ of carbons and the Zn/C proportion of the MOFs could be only applied to the MOF precursors constructed from rigid linkers. Furthermore, the former carbons contained larger pores (5–10 nm) than the latter carbons (2–7 nm). Interestingly, the flexibility of the linker could control the electrochemical performance of SSC assembled from the relevant porous carbons in 2E symmetric cell with 1 M H_2_SO_4_ electrolyte. The specific capacitance of each carbon was mainly dependent on the surface area.

Many other reports that introduced different aromatic linkers [39,40,41,42,43] or aliphatic linkers [44,45,46] into the Zn-MOF templates were published. The former reports are further divided into two parts based on whether bdc modified linkers were employed [39,40] or not [41,42,43]. A NH_2_-bdc or 2-aminobenzene-1,4-dicarboxylic acid-based Zn-MOF (IRMOF-3) was used as a self-sacrificing template for the scalable synthesis of a hierarchical N-doped porous carbon (N-doping level = 3.3%) [39]. With no extra nitrogen- or carbon-containing precursors and purification process, CIRMOF-3-950 was prepared from a single-step carbonization of the template at 950 °C (6 h, Ar flow). Other CIRMOF-3-s (s = carbonization temperature) carbons carbonized at lower temperature required further HCl treatment to remove residual ZnO. Compared with them, the CIRMOF-3-950 had high hierarchical porosity and graphitization degree. A N-free-control CMOF-5-950 derived from MOF-5 exhibited similar pore features to the CIRMOF-3-950. However, there were different electrochemical properties between CIRMOF-3-950 and CMOF-5-950 electrodes for SSC operated in 1.0 M H_2_SO_4_. The difference might be originated from the N-dopant related factors, such as improved electrolyte wettability (electrolyte-electrode interaction), reduced carbon defects, and additional pseudocapacitance. As a result, the CIRMOF-3-950 showed good specific capacitances of 239 F g^−1^ at 5 mV s^−1^ and 212 F g^−1^ at 0.5 A g^−1^. However, the CMOF-5-950 showed only 24 F g^−1^ at 0.5 A g^−1^. In addition, the observed specific capacitances of CIRMOF-3-950 were 166 F g^−1^ at 100 mV s^−1^ and 162 F g^−1^ at 10 A g^−1^. These high rate capabilities were resulted from efficient electrolyte diffusion facilitated by the hierarchical pore structure.

A facile and effective method to prepare an outstanding 2D carbon electrode for supercapacitor was successfully accomplished [40]. MOF-74 with salicylic acid as a modulator facilitating rod-shaped morphology was synthesized from the anhydrous zinc acetate and 2,5-dihydroxyterephthalic acid under mild reaction condition at room temperature. Without another catalyst or carbon source, MOF-74-Rod self-sacrificing template was transformed into 1D-carbon nanorods (CNrod) by simple carbonization at 1000 °C (4 h, Ar flow). Subsequently, 2D-graphene nanoribbons (GNRib) composed of two- to six-laminated structure was obtained with high yield (>75%) and purity (>90%) by sonochemical KOH treatment of the CNrod followed by pyrolytic activation at 800 °C (2 h, Ar flow) with acid washing (1 N HCl). The GNRib exhibited high S_BET_ and electrical conductivity (4.93 S cm^−1^). Furthermore, it held 2D laminated structure for enhanced connectivity and interlayer void for easy ionic accessibility. Thus, the GNRib was a good electrode material for SSC. The specific capacitances obtained from CV are 193 and 123 F g^−1^ at 10 and 400 mV s^−1^, respectively, in 1 M H_2_SO_4_ electrolyte. The electrode could operate in ionic liquid of 1-ethyl-3-methylimidazolium dicyanamide with the extension of applied voltage from 1.0 to 3.0 V. It delivered high specific capacitances of 273 F g^−1^ at 10 mV s^−1^ and 227 F g^−1^ at 0.05 A g^−1^.

**Figure 2:**
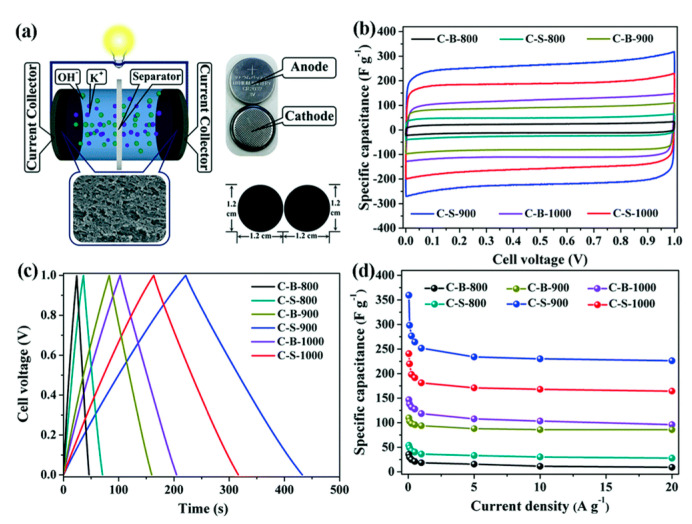
(**a**) Illustrations of the SSC in 6 M KOH electrolyte; (**b**) CV curves at 50 mV s^−1^ for C-B-n and C-S-n; (**c**) Galvanostatic charge-discharge (GCD) curves at 0.5 A g^−1^ for C-B-n and C-S-n; (**d**) dependence of specific capacitance on applied current densities [41].

Zn(thip) (thip = 5-*tert*-butyl isophpthalate) was employed as a precursor for the preparation of 3D sponge shaped porous carbons (PCs) with interconnected hierarchical porosity as shown in Figure 2 [41]. From a single-step carbonization (2 h, Ar flow) of two different particle sized Zn-MOFs (Zn(thip)-B and Zn(thip)-S), the corresponding carbon samples (C-B-n, C-S-n, n = carbonization temperature in °C) were obtained. C-S-900 was the best sample for good structural and electrochemical properties. The hierarchically porous C-S-900 had high S_BET_ and V_total_ (2.72 cm^3^ g^−1^). These structural characteristics could effectively improve ion diffusion/transport, electrochemically accessible surface area, and conductivity. In addition, maximum energy density of 12.5 W h kg^−1^ and maximum power density of 7200 W kg^−1^ were obtained. A flexible all-solid-phase device for SSC was investigated in PVA/KOH electrolyte, too. Its highest areal specific capacitance was found to be 159 mF cm^−2^ at 0.15 A cm^−2^. Furthermore, high mechanical stability was shown up to bending angle of 180°.

There was an interesting report of carbon derived from an anionic Zn-MOF containing 4,4′-biphenyldicarboxylate (bpdc) bridging ligand [42]. In the report, a cation-exchanged form of the anionic Zn-MOF template has been prepared and carbonized for the sake of generating well-developed microporosity of the resultant MOF-derived carbon through in situ activation by the encapsulated K^+^ ions. The anionic bio-MOF-1 has a formula of [Me_2_NH_2_]_2_[Zn_8_(ad)_4_(bpdc)_6_] (ad = adeninate). The K^+^ ion-encapsulated bio-MOF-1 (K@bio-MOF-1) was prepared from a DMF solution of 0.1 M KNO_3_. [Me_2_NH_2_]^+^ ions were easily exchanged with K^+^ ions. After carbonization of the templates (4 h, N_2_ flow) and followed by 2 M HCl treatment, mesoporous BM-T (from bio-MOF-1) and microporous KBM-T (from K@bio-MOF-1) were produced (T = carbonization temperature in °C). As a result of effective in situ K^+^-activation, KBM-700 with a narrow pore size distribution has high values of S_BET_, V_micro_ ratio (73%), and nitrogen content (10.16%). Its 3E cell also showed a good electrochemical performance in 6 M KOH electrolyte. It showed high specific capacitance of 230 F g^−1^ at 1 A g^−1^. These excellent properties could be ascribed to the hierarchical microporosity as well as the high N-dopant content.

PFA/Zn-btc-MOF composite was used to synthesize microporous carbons (MPCs) [43]. The composite was prepared by incorporating FA secondary carbon source into the bare MOF template and subsequent polymerization of the encapsulated FA. After a single-step carbonization of the composite at 950 °C (6 h, Ar flow), the resultant MPC-950 showed high S_BET_ with large V_total_ (2.013 cm^3^ g^−1^). Furthermore, improved carbon yield (28%) and a wide range of pore regions (micropore, mesopore, and macropore) were accomplished for MPC-950. Combined with good S_BET_ and V_total_, these various pore regions played a key role in enhancing the specific capacitance of MPC-950-based working electrode for 3E system in 6 M KOH electrolyte. The electrode showed high specific capacitances of 471 F g^−1^ at 2 mV s^−1^ and 320 F g^−1^ at 100 mV s^−1^.

Wang et al. published two papers about carbon materials derived from Zn-glutamate MOF [44,45]. In the first paper, a simple and green synthetic approach for carbon precursor of [Zn(L-glu)H_2_O∙H_2_O]_n_ microrods (L-glu = L-glutamate) was reported [44]. The carbon precursor was synthesized from the aqueous solution of zinc acetate hydrate and monosodium glutamate (MSG) at room temperature. In addition, the 3D nanoporous material with hierarchical porosity was obtained from the MOF microrods via carbonization (2 h, N_2_) and acid treatment with 1 M HCl. The resultant carbon with co-doping of N and O had interconnected microporous structure with thin shell cavity. Among three carbons (S700, S800, and S900) prepared from different carbonization temperatures, S800 contained well-developed microporosity (S_micro_ = 403 m^2^ g^−1^, V_micro_ = 0.21 cm^3^ g^−1^) despite smaller S_BET_. As a working electrode material for 3E cell in 6 M KOH electrolyte, the S800 exhibited best performance among them. The specific capacitances were 418 F g^−1^ at 10 mV s^−1^ and 276 F g^−1^ at 200 mV s^−1^. In this electrode, a high N-doping level (7.67%) improved electronic conductivity and surface wettability for effective ionic transport. Moreover, 3D interconnected hierarchical porosity enhanced the charge accommodation. In the second paper, another simple and green synthetic approach for the electrostatically assembled graphene oxide (GO)-MOF composite-based carbon precursor was reported [45]. The etching of MOF facets by GO sheets was discovered for the first time. The GO-MOF composite was prepared from positive GO sheets and a negative Zn-glutamate MOF under aqueous condition. The GO-MOF composite derived porous carbons were synthesized from carbonization (2 h, N_2_) and subsequent acid washing (1 M HCl). The heteroatom-doped and 3D-interconnected porous carbons showed ‘’egg-shell” architecture with crumpled graphene thin sheets. Among the final three carbon products (S800, S900, and S1000) from different carbonization temperatures, S900 exhibited an improved microporosity (S_micro_ = 567 m^2^ g^−1^, V_micro_ = 0.24 cm^3^ g^−1^). Therefore, S900 carbon working electrode showed good capacitive performance in 3E system using 6 M KOH electrolyte. At 20 mV s^−1^, its gravimetric and volumetric specific capacitances were 318 F g^−1^ and 299 F cm^−3^, respectively. Both hierarchical porosity (micro-/mesopore) and 3D-interconnected cellular structure may have synergistic effects on enhanced accommodation of charges. Furthermore, 2D graphene architectures improved conductivity and speed of electronic double-layer formation.

A simple and economical fabrication of mesoporous carbon nanofibers was achieved from the self-sacrificing template of zinc glycolate [46]. The self-sacrificing template was prepared from ethylene glycol (EG) solution of cheap zinc acetate. After heat treatment of the zinc glycolate fibers at 600 °C (2 h, Ar flow) and subsequent 2.0 M HCl treatment, the mesoporous carbon nanofibers (CNFs) were successfully synthesized. The CNFs showed 1D-fibrous nanostructure, high S_BET_, large V_total_ (2.7 cm^3^ g^−1^), well-defined micropores (1.1 nm) and mesopores (3.4 and 10~20 nm), 3D interconnected mesoporosity, and abundant surface oxygen functionalities (~9 wt.%). As a result, the CNFs-based working electrode showed good capacitive performance in both 6 M KOH electrolyte (3E cell) and 1.0 M Et_4_NF_4_/PC (PC = propylene carbonate; 2E cell).

Two other reports using pyridine-containing linker or dabco (dabco = 1,4-diazabicyclo [2.2.2] octane) as N-dopant sources were published [47,48]. Two N-doped porous carbons (NPCs) derived from ([Zn(bpydc)DMA]∙DMF (bpydc = 2,2′-bipyridine-5,5′-dicarboxylate, DMA = dimethyl acetamide, DMF = *N*,*N*-dimethylformamide, the abbreviation of “bpydc” is used here instead of “bpdc” to avoid any confusion with the same term for 4,4′-biphenyldicarboxylic acid) were synthesized from direct carbonization (3 h, N_2_ flow) at two different temperatures (800 and 1000 °C) [47]. The NPC800 showed a higher S_BET_ with a larger V_pore_ as well as a higher N-dopant content than NPC1000. On the contrary, it held better graphitization degree and a good conductivity than NPC800. As a working electrode of supercapacitor for 3E system operating in 6 M KOH electrolyte, the NPC800 possessed superior electrochemical properties than the NPC1000. Synergistic contributions of EDLC (from high surface area and mesoporosity) and pseudocapacitance (from nitrogen contents) could effectively support the result.

Schmidt et al. investigated a facile and effective preparation of nano-MOF with solvent controlled morphology and its influence on volumetric capacitance of the resultant carbon electrode [48]. The dual roles of solvent in mild conditional synthesis of [Zn_2_(bdc)_2_(dabco)]_n_ (ZBD) were template and crystallization modulator. The selective formation of either an 1D-hexagonal rod MOF (ZBDh) or a 2D-tetragonal plate MOF (ZBDt) was induced by DMF and MeOH, respectively. One-step direct carbonization of the ZBDs at 900 °C (2 h, Ar flow) produced nanoporous carbons of ZBDh-D10-900 (hexagonal nanorods) and ZBDt-M10-900 (tetragonal nanoplates). They had similar graphitization degree and nitrogen content (~4.2 wt.%) but different S_BET_ (1490 m^2^ g^−1^ for ZBDh-D10-900 and 1230 m^2^ g^−1^ for ZBDt-M10-900). EDLC-type supercapacitive performance of symmetric ZBD-derived carbon electrodes was measured in 1 M TEABF_4_/AN electrolyte. The two electrodes showed similar gravimetric capacitances. However, the volumetric capacitances of the 2D tetragonal plate carbon electrode were almost 2-fold larger than those of hexagonal carbon rod in the scan rate ranging from 10 to 500 mV s^−1^. The 2D morphology and comparatively high packing density could account for this result.

For pure and heteroatom-doped carbons, the electrodes with high S_BET_ usually exhibited high capacitances. The surface area of MPC-950 with the highest gravimetric capacitance of 471 F g^−1^ is as large as 1455 m^2^ g^−1^ as shown in Table 1. Although there are additional critical factors such as suitable pore dimension and pore structure for capacitance enhancement, the surface area of the porous carbons from MOFs is primarily important.

#### 2.1.3. ZIF-8-Derived Carbons

Porous carbons can be efficiently prepared from zeolitic imidazolate frameworks (ZIFs). Notably, ZIFs contain imidazole-based bridging ligands which are potential N-dopant sources. Since the carbonization of ZIF-8 was most intensively investigated, the electrochemical capacitive properties of the porous carbons derived from ZIF-8 are summarized in Table 2. ZIF-derived carbon electrodes for supercapacitor were first reported by Xu et al. [49]. ZIF-8, one of the most famous zeolite-type MOFs, has been chosen as both a primary carbon source and a sacrificial self-sacrificing template for N-doped porous carbon due to its high chemical and thermal stabilities along with high porosity. As a secondary carbon source, FA was infiltrated into thermally activated ZIF-8 for the construction of FA/ZIF-8 composite despite the limited accessibility of FA to small ZIF-8 pores. After polymerization of FA, the FA/ZIF-8 composite was transformed into PFA/ZIF-8 composite. Carbonization of the PFA/ZIF-8 composite at different temperatures at 800 or 1000 °C (8 h, Ar flow) was conducted to produce the N-doped porous carbon materials (C800 or C1000). C1000 contained higher S_BET_ and V_pore_ than C800. Both N-doped carbon electrodes of SSC operating in 1 M H_2_SO_4_ electrolyte exhibited high specific capacitance about 200 F g^−1^ at 0.250 A g^−1^. They also showed excellent rate performances (188 F g^−1^ for C800 and 161 F g^−1^ for C1000 at 5 mV s^−1^, 160 F g^−1^ for C800 and 137 F g^−1^ for C1000 at 50 mV s^−1^).

The nanoporous carbon series of Z-n (n = carbonization temperature in °C) was synthesized from direct carbonization of ZIF-8 (5 h, N_2_) and following 10 wt.% HF solution treatment [50]. Except for Z-600, the resultant carbons exhibited high surface areas and narrow porosity. Z-900-based working electrode of 3E cell with 0.5 M H_2_SO_4_ electrolyte had gravimetric capacitances of 214 F g^−1^ at 5 mV s^−1^ and 115 F g^−1^ at 100 mV s^−1^. In addition, the electrode held the highest volumetric capacitance of 200 F cm^−3^ at 5 mV s^−1^ among the reported values for MOF-derived carbon electrodes at that time.

Xu et al. reported the 3D carbon framework with well-interconnected hierarchical porosity derived from ZIF-8 [51]. Microporous ZIF-8 NPs were prepared from ultra-sonification method, and additional micro-/macropores of the ZIF-8 were also generated by sonochemically induced interparticle spaces. The resultant S-ZIF-8 with hierarchical porosity was transformed into S-ZC-800 via direct carbonization at 800 °C (10 h, Ar flow) and subsequent HCl (5 vol% in H_2_O) treatment. Hierarchically porous AS-ZC-800 was obtained by KOH activation of the S-ZC-800 (C/KOH = 0.5; 800 °C, 1 h, Ar flow). The AS-ZC-800 carbon framework showed high S_BET_ and large V_total_ (2.56 cm^3^ g^−1^) in addition to well-interconnected micro-, meso-, and macropores. The AS-ZC-800 electrode for SSC under 1 M H_2_SO_4_ electrolytic condition presented high specific capacitances and excellent capacitive rate performance (187 F g^−1^ at 400 mV s^−1^ and 204 F g^−1^ at 50 A g^−1^).

A single-template synthesis of N-doped mesoporous carbons (NMCs) with ordered morphology were achieved by only a single-step carbonization at 1000 °C (2 h, N_2_ flow) of nano-sized ZIF-8 polyhedrons [52]. The NMCs exhibited ultra-high S_BET_ of 2737 m^2^ g^−1^ as well as dominant mesoporosity (average D_pore_ = 4.27 nm, the ratio of mesopore volume/total pore volume = 74.09%) with moderate N-doping level (4.84 at%). Under 3E supercapacitor cell at 1 M H_2_SO_4_ electrolyte, it showed 307 F g^−1^ at 1 A g^−1^ and 182.5 F g^−1^ at 50 A g^−1^. It also had great capacitive rate capability of 74.6% retention when the scan rate increased from 10 mV s^−1^ (212.1 F g^−1^) to 1000 mV s^−1^ (158.3 F g^−1^). The efficient formation of electrical double-layer (EDL) from mesoporosity-driven facile ionic transport, nitrogen functional group induced enhancement of electrode wettability, effective redox reaction may synergistically operate for the superior performances.

An easy and cost-effective synthetic strategy was developed for ZIF-8-based carbon series with bimodal micro-/mesoporosity [53]. With or without incorporation of extra porogenes of silica colloids (fumed silica or Ludox) into a sacrificial template of ZIF-8, carbonization (800 °C, 5 h, Ar flow) of the as-prepared ZIF-8 or silica/ZIF-8 and subsequent 10 wt.% HF treatment generated a series of hierarchical porous carbons (C-ZIF-8, LC-ZIF-8, and FC-ZIF-8). In addition to microporous ZIF-8 cavities and mesopores from self-aggregation of carbon NPs, the colloidal silica/ZIF-8 derived carbon samples had additional mesopores (3 nm for LC-ZIF-8, and 17 nm for FC-ZIF-8) resulted from self-assembly or elimination of additional silica porogenes. The resultant mesoporosity of the carbon series greatly influenced electrochemical performance of the working electrode for 3E system operating in 6 M KOH electrolyte. At a scan rate of 5 mV s^−1^, the specific capacitances of C-ZIF-8, LC-ZIF-8, and FC-ZIF-8 were 181, 175, and 164 F g^−1^, respectively. Interestingly, these values matched well with the decreasing order of micropore volumes (0.23, 0.16, and 0.15 cm^3^ g^−1^ of C-ZIF-8, LC-ZIF-8, and FC-ZIF-8) possibly because of the critical role of micropore for charge storage. In addition, mesopore volumes of the carbons greatly influenced equivalent series resistance (ESR) and capacitive retention performance due to their different ionic transfer abilities. Therefore, the ESR values for C-ZIF-8, LC-ZIF-8, and FC-ZIF-8 were 0.21, 0.25, and 0.42 Ω cm^2^, respectively.

Another easy and economical synthetic approach was applied to nanoporous carbon (NPC) via direct carbonization of particle size-controlled ZIF-8 self-sacrificing template at 800 °C (5 h, N_2_ flow) and subsequent 10% HF treatment [54]. The obtained NPC had high S_BET_ with microporosity (mean D_pore_ = ~1 nm). With the help of polyvinylpyrrolidone (PVP) modulator, a large-size ZIF-8 self-sacrificing template was prepared and could be transformed into a large-size NPC. Under 3E configuration in 1 M H_2_SO_4_ electrolyte, the large-size NPC showed superior capacitance (251 F g^−1^) at 5 mV s^−1^ than the small-size NPC (125 F g^−1^) because particle aggregation limiting the electrolyte transport was not observed. In addition, the SSC from the large-size NPC within the same electrolyte showed high cell capacitance at 0.5 A g^−1^ (98 F g^−1^). It also delivered a high energy density of 10.86 W h kg^−1^ with a power density of 225 W kg^−1^ at 0.5 A g^−1^. In addition, at 5 A g^−1^, the specific energy was slightly decreased (6.97 W h kg^−1^) but the specific power was largely increased (2281 W kg^−1^).

A simple single-step co-carbonization of ZIF-8 with secondary carbon precursors (such as melamine, urea, xylitol, and sucrose) at 950 °C (5 h, Ar flow) was performed to synthesize a series of N-doped porous carbon electrodes operating in basic electrolyte [55]. The ZIF-8/secondary carbon source co-derived carbons (Carbon-ZM, Carbon-ZU, Carbon-ZX, and Carbon-ZS) with hierarchical porosity exhibited higher S_BET_ and V_total_ as well as specific capacitances than bare ZIF-8 derived carbon (Carbon-Z). During the carbonization, pre-melting and polymerization of the additional carbon precursor incorporated into the ZIF-8 self-sacrificing template could facilitate the graphitization and construct a protective layer for the passivation of structural collapse and nitrogen loss from the ZIF-8. As a result, the Carbon-ZS showed good electrical conductivity with moderate N-dopant level (4.5%). Therefore, it was the best working electrode for supercapacitor with 3E in 6 M KOH electrolyte among the five samples.

Yamauchi et al. introduced organic electrolyte into ZIF-8-derived carbon-based supercapacitor [56]. Simple direct carbonization of ZIF-8 at 900 °C (5 h, N_2_ flow) and subsequent HF (10 wt.%) treatment were conducted to prepare N-doped nanoporous carbon (NPC). The N-doped NPC had high S_BET_ with micro-/mesoporosity. At 10 mV s^−1^, the NPC-based SSC in 2 M NEt_4_BF_4_/PC electrolyte exhibited stack cell capacitance of 9.24 F cm^−3^ and gravimetric cell capacitance of 21.0 F g^−1^. The supercapacitor showed low ESR values, which resulted from low ionic resistance within the pores. At 0.001 Hz, it also held high specific cell capacitances (9.10 F cm^−3^ and 20.6 F g^−1^). Its stack cell capacitance obtained at 5 mA was 4.23 F cm^−3^ (gravimetric cell capacitance = 10.6 F g^−1^). Its peak value of volumetric energy was found to be ~6.6 mW h cm^−3^ with ~0.64 W cm^−3^ of volumetric power.

A single-template synthesis of 3D hybrid-porous carbon with hierarchical porosity has been successfully accomplished by the carbonization of ZIF-8 microcrystal at 950 °C (3 h, N_2_ flow) [57]. The obtained bimodal porous carbon (micro- and mesopores) showed high S_BET_ and large V_total_ of 0.693 cm^3^ g^−1^. Porosity of the carbon was micropore-dominant (S_micro_ = 747 m^2^ g^−1^, V_micro_ = 0.380 cm^3^ g^−1^). Using electrode of SSC in 1 M KOH electrolyte, it showed high specific capacitance of 332 F g^−1^ with volumetric capacitance of ~400 F cm^−3^ at 500 mA g^−1^. It yielded high capacitive rate capability at 40 A g^−1^ as well. It held superior surface properties for good ionic accessibility and low resistance for ionic diffusion.

Yamauchi et al. conducted direct carbonization of ZIF-8 crystals (5 h, N_2_ flow) and following HF (10 wt.%) treatment to prepare nanoporous carbon materials [58]. The resultant carbons stood for S-n (n = carbonization temperature in °C). S-900 sample had highest values of S_BET_ and V_total_. In addition, its hierarchical micro-/mesoporous bimodal porosity and suitable ratio of micropore/mesopore were critical factors for electrochemical performance in 1 M H_2_SO_4_ electrolyte. Under 3E system, the S-900 yielded 219 F g^−1^ at 5 mV s^−1^ and ~109 F g^−1^ at 500 mV s^−1^. In addition, S-900-based SSC showed a maximum specific capacitance value of 53.8 F g^−1^ (or 23.6 F cm^−3^ or 11.80 μF cm^−2^) with high capacitance retention of 73.9% at 2 A g^−1^.

Production of hollow particle-based N-doped carbon nanofibers (HPCNFs) with hierarchical porosity was effectively achieved by two-step pyrolysis (550 °C, 1 h and 900 °C, 2 h) of the electrospun 1D ZIF-8/PAN (PAN = polyacrylonitrile) composite precursor under N_2_ atmosphere and subsequent 3.0 M H_2_SO_4_ treatment [59]. The 1D structure and high nitrogen content of the ZIF-8 NP/PAN composite were well preserved in the HPCNFs-N. Compared to N-doped carbon (C-N; S_BET_ = 223 m^2^ g^−1^) derived from bare ZIF-8 particles, the HPCNFs-N exhibited improved S_BET_ value of 418 m^2^ g^−1^. Under 2.0 M H_2_SO_4_ electrolyte, SSC assembled from the HPCNFs-N electrode showed 307.2 F g^−1^ at 1.0 A g^−1^ and 193.4 F g^−1^ at 50.0 A g^−1^. The SSC also had good recycling stability of 98.2% retention of initial capacitance at 5.0 A g^−1^ after 10,000 cycles. Enhancements of electrochemical active sites and electrochemical kinetics as well as structural stability were due to the hierarchical porosity from many interconnected hollow carbon NPs. Furthermore, good ionic and electronic transport were resulted from the 1D fibrous structure.

Other N-doped hierarchical nanoporous carbon fibers (NPCFs) with 1D hollow structure was synthesized from two-step thermolysis (240 °C, 1 h and 800 °C, 3 h) of electrospun ZIF-8/PAN nanofibers under N_2_ [60]. The optimized sample of NPCFs-0.6 had S_BET_ of 315 m^2^ g^−1^ and V_total_ of 0.33 cm^3^ g^−1^. The NPCFs-0.6 showed a high specific capacitance of 332 F g^−1^ at 1 A g^−1^ under 3E system with 1 M H_2_SO_4_ electrolyte. The N-doped hollow structure of the NPCFs-0.6 with hierarchical micro-/mesoporosity generated high conductivity of 7.74 S cm^−1^ for fast electronic and ionic transport.

The electrospinning method was also employed for the synthesis of N-enriched hierarchically porous carbon nanofibers (NHCFs) for binder-free and flexible electrode [61]. The ZIF-8/PAN composite precursor was transformed into the NHCFs by thermal stabilization at 250 °C (120 min, air), carbonization (2 h, Ar flow), and HCl treatment. After fine control of ZIF-8 contents (66.7 wt.% ZIF-8 contained PAN) and carbonization temperature (800 °C), the optimized NHCF-66.7% ZIF-8 sample with hierarchical and interconnected porosity was obtained. It held high values of S_BET_ (559.63 m^2^ g^−1^), V_total_ (0.789 cm^3^ g^−1^), and N-dopant content (15.59 wt.%). In addition to these favorable textural properties, both pseudocapacitance from N-/O-functionalities and good electrical conductivity from high graphitization degree made the NHCF-66.7% ZIF-8 had the best capacitive performance for 3E cell in 6 M KOH electrolyte.

Kim et al. reported a binder-free working electrode made of B/N-co-doped 3D porous carbon nanofiber network derived from the electrospun PAN/ZIF-8 raw material [62]. After electrospinning embedment of ZIF-8 NPs into PAN nanofiber, NABH_4_ treatment of the PAN/ZIF-8 nanofiber was performed to preserve 3D structure. B/N-co-doping of the NaBH_4_ treated PAN/ZIF-8 was achieved by wet chemical treatment with ammonium borate (NH_4_HB_4_O_7_∙3H_2_O). The product 3D-BN-CNF-ZF-900 was obtained from the following successive steps of freeze-drying (for maintaining cross-linking), stabilization at 250 °C (2 h, air), carbonization at 900 °C (2 h, N_2_ flow), and acid washing (2 M H_2_SO_4_). The 3D-BN-CNF-ZF-900 had 3D-layered structure, cross-linked network, B/N-co-doping (B = 1.3 at%, N = 11.24 at%), S_BET_ of 352 m^2^ g^−1^, high V_pore_ of 1.789 cm^3^ g^−1^, mesoporosity, and hydrophilic character (contact angle = 0°). Therefore, as a binder-free working electrode of supercapacitor with 3E cell in 2 M KOH electrolyte, it held high specific capacitance of 295 F g^−1^ at 0.5 A g^−1^ with good rate performance (50% retention) of 147 F g^−1^ at 5 A g^−1^. In addition, its normalized areal capacitance at 0.5 A g^−1^ was 83.9 μF cm^−2^.

The electrospinning method was applied to the synthesis of polymer nanofibers [63]. The electrospun polymer nanofiber was decorated with a monolayer sheath of ZIF-8 particles with uniform size and morphology by polarity-assisted approach. The strong interaction between polar–carbon nanosheets (CN) group on PAN side chains and Zn^2+^ ions initiated nucleation and coordinative growth of ZIF-8. The obtained PAN@ZIF-8 hybrid nanofibers were transformed into N-doped nanofibrous carbon by dual-step pyrolysis (550 °C, 2h and 900 °C, 3 h under N_2_ flow). From the fine-tuned molar ratio of 8 organic ligand to 1 metal ion, the optimized N-NFC-8 with a hollow frame had S_BET_ of 277.2 m^2^ g^−1^ and high doping of heteroatoms (N = 7.68 at%, O = 12.83 at%). When the N-NFC-8 was used as a working electrode for supercapacitor with 3E cell in 1 M H_2_SO_4_ electrolyte, its specific capacitance at 1 A g^−1^ was as high as 387.3 F g^−1^. A symmetric N-NFC-8-based supercapacitor operating in the same electrolyte exhibited maximum cell capacitance of 85.0 F g^−1^ at 1 A g^−1^ and the rate retention of 60% at 10 A g^−1^. Solid-state SSC fabricated from the N-NFC-8 electrode and PVA-H_2_SO_4_ gel showed the highest cell capacitance of 42.2 F g^−1^ at 5 A g^−1^. The device held good flexibility at different bending angles and good recycling performance of ~90% retention after 10,000 cycles at 1 A g^−1^. Highly conductive core containing 1D nanofiber facilitated electronic transport. Additionally, the incorporated heteroatoms (N and O) rendered extra electrochemical active sites. Moreover, a monolayer sheath of hollow carbon frames decreased the interfacial resistance and increased ionic transport with favorable electrode kinetics.

**Figure 3:**
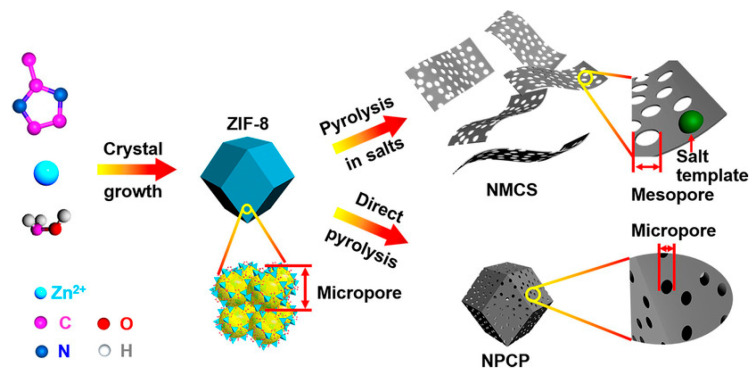
Synthetic procedure of NMCS and NPCPs (N-doped porous carbon polyhedrons) with or without the mediation of molten salts [64].

Inorganic molten salts such as LiCl and KCl were employed for the synthesis of ZIF-8 derived carbon with 2D sheet structure and mesopores (Figure 3) [64]. From the carbonization of ZIF-8/molten salt mixture at 800 °C (5 h, N_2_ flow) followed by 0.5 M HCl treatment, N-doped mesoporous carbon nanosheets (NMCS) with 2D structure and hierarchical porosity were successfully prepared due to the activation of molten salts. The series of 2D carbon nanosheets were denoted as NMCS-X (X = carbonization time in h). Compared to bare ZIF-8 derived N-doped porous carbon polyhedron (NPCP-5) with dominant microporosity (S_meso_ = 37 m^2^ g^−1^, V_meso_ = 0.398 cm^3^ g^−1^), the three NMCS-X have well-developed mesoporosity with high S_meso_ (200–407 m^2^ g^−1^) and large V_meso_ (0.425–0.886 cm^3^ g^−1^). As a result, they hold higher S_BET_ and larger V_total_ than the NPCP-5. The improvements of ionic accessible active sites from mesoporosity, wettability from N-doped polar surface, and electrode packing, and ionic conductivity from the 2D nanosheet structure with large aspect ratio synergistically supported the superior electrochemical performance of NMCS-X-based supercapacitor (6 M KOH, 3E cell).

It is clearly revealed that porous carbons directly derived from ZIF-8 are supercapacitor electrodes with good performance (Table 2). The unique crystal structure and pore geometry of ZIF-8 provided ideal carbon matrices for supercapacitors upon pyrolysis.

#### 2.1.4. Other ZIF-Derived Carbons

The electrochemical performances of porous carbon electrodes derived from other ZIFs are collected in Table 3. Generally, their capacitances are a little bit lower than those of ZIF-8-derived carbon electrodes depicted in Table 2. Thus, ZIF-8 may be a superior precursor for porous carbons compared to other ZIFs.

The correlation between local structure of carbon products and composition of carbon sources was systematically investigated by employment of three ZIFs (ZIF-8, ZIF-68, and ZIF-69) [65]. Direct carbonization of the ZIFs at 1000 °C (5 h, Ar) yielded carbonization products (CZIF8, CZIF68, and CZIF69). After KOH activation of the resultant N-doped carbons at 750 °C (1 h, Ar flow, mass ratio of C/KOH = 0.2), the activated carbon materials (CZIF8a, CZIF68a, and CZIF69a) were obtained. Macropores were only displayed in benzimidazolate-containing ZIF-based carbons (CZIF68, CZIF69, CZIF68a, and CZIF69a). The chloride functional group in ZIF-69′s benzimidazole ligand was able to greatly influence the structure and property of carbonized porous products. As a result, CZIF69a showed high S_BET_, large V_total_ (1.184 cm^3^ g^−1^), and hierarchical micro-/mesoporosity. Therefore, as a working electrode of 3E cell supercapacitor in 0.5 M H_2_SO_4_ electrolyte, it yielded 168 F g^−1^ at 5 mV s^−1^ and 135 F g^−1^ at 200 mV s^−1^.

A simple co-carbonization (5 h, Ar flow) of ZIF-7 with additional green carbon precursors (glucose, ethylene glycol, glycerol, and FA) was employed to prepare a series of conducting agent-free carbon electrodes [66]. The resultant carbons were Carbon-L-T (T = carbonization temperature in °C, L = glucose), Carbon-E-950 (ethylene glycol), Carbon-G-950 (glycerol), Carbon-F-950 (FA), and Carbon-Z-950 (carbon source free ZIF-7), respectively. The optimized Carbon-L-950 sample contained high S_BET_ and large V_total_. It also had hierarchical micro-/mesoporosity with conductive graphene-like structure as well as good surface properties (hydrophilicity and wettability). When applied to conducting agent-free working electrode of 3E cell in 6 M KOH electrolyte, its maximum specific capacitance at 0.1 A g^−1^ was 228 F g^−1^.

Flexible and free-standing carbon electrodes (Z800, Z900, and Z1000) with no current collector and separating substrate were synthesized from the thermal stabilization for cross-linking at 280 °C (30 min, air flow) and subsequent carbonization of electrospun ZIF-7/PAN composite (60 min, Ar flow) [67]. The ZIF-7/CNF (CNF = carbon nanofiber) was simply prepared in a large scale with an optimal PAN concentration of 7 wt.%. In addition, as an electrode for SSC operating in 6 M KOH electrolyte, Z950 (the sample carbonized at 950 °C) showed the best performance among the three samples.

Zhang et al. prepared ZIF-11-derived carbon materials. After single-step carbonization of benzimidazole containing ZIF-11 polyhedrons at 1000 °C (2 h, N_2_ flow), the N-doped porous carbon micropolyhedrons (N-PCMPs) were successfully synthesized [68]. The activated forms of N-PCMPs (N-PCMPs-A) were also generated from KOH activation of the N-PCMPs (mass ratio of C and KOH = 1:5; 750 °C, 1 h, N_2_). The N-PCMPs-A with improved mesoporosity had higher S_BET_ (2188 m^2^ g^−1^) and V_total_ (1.24 cm^3^ g^−1^) than the N-PCMPs (895 m^2^ g^−1^, 0.58 cm^3^ g^−1^). In addition, compared to the N-PCMPs, O- and N-containing functional groups of the N-PCMPs-A were more exposed to the carbon surface. Therefore, as a working electrode of 3E cell with 1.0 M H_2_SO_4_ electrolyte, the N-PCMPs-A was superior to the N-PCMPs. At a scan rate of 1 mV s^−1^, their specific capacitances were 295 F g^−1^ for N-PCMPs-A and 180 F g^−1^ for N-PCMPs. When the scan rate increased to 50 mV s^−1^, the N-PCMPs-A exhibited better capacitance retention (194 F g^−1^) than the N-PCMPs (106 F g^−1^).

The highest specific capacitance of 283 F g^−1^ at 10 A g^−1^ among the already reported MOF-derived carbon nanosheet electrodes was achieved by the ultrathin carbon nanosheets (UT-CNSs) in 2018 [69]. He et al. reported the effective and economical bottom-up synthetic strategy for 2D ultrathin Zn(bim)(OAc) (UT-Zn(bim)(OAc), bim = benzimidazole) and N-doped porous UT-CNSs derived from Zn(bim)(OAc). From the hydrophilic gluconate-aided synthetic approach, the UT-Zn(bim)(OAc) self-sacrificing template with a small thickness (~5 nm) was prepared in high yield (~65%). The corresponding slightly less thickened (~2.5 nm) UT-CNSs were produced in high yield (45.7%) by the direct carbonization of the template (800 °C, 3 h, Ar/H_2_ 10 vol%) and subsequent 1 M HCl treatment. The UT-CNSs had many promising properties for the improved electrochemical performance such as high graphitization degree, few layered ultrathin nanosheets morphology, high S_BET_, N-doping, and hierarchical porosity. Consequently, key favorable factors for supercapacitive properties of UT-CNSs-based working electrode were displayed such as high electric conductivity of 4.5 S cm^−1^, small contact resistance, facilitated charge transfer and ionic diffusion, and highly effective specific surface area for ion storable capacity.

N-decorated nanoporous carbons (NNPCs) were derived from ZTIF-1 containing 5-methyltetrazole and 2-ethylimidazole ligands [70]. From carbonization for 2 h and HCl etching, the TZIF-1 polyhedral crystals could be converted into NNPCs (NNPC-700, NNPC-800, and NNPC-900). The NNPC-800 had high values of S_BET_ as well as N-dopant content (14.23 wt.%), and contained micro-/mesoporous hierarchical porosity. This carbon working electrode of 3E cell in 6 M KOH electrolyte showed 272 F g^−1^ at 0.1 A g^−1^. Its maximum specific capacitance in the same electrolytic 2E system at 0.1 A g^−1^ was 154 F g^−1^. At 2 A g^−1^, it showed 57% capacitive retention.

### 2.2. Other MOF-Derived Carbons

Table 4 summarizes the electrochemical performances of porous carbons derived from other types of MOFs. Depending on MOF precursors, the surface areas and capacitances show a wide range of values. It is notable that capacitances are strongly dependent on the surface area. Thus, the use of MOF precursors which can give high-surface porous carbons is primarily important for better supercapacitors.

#### 2.2.1. Al-MOF-Derived Carbons

An unprecedented double-template method using Al-MIL-101-NH_2_ and the encapsulated Cu^2+^ ions was introduced as a simple and effective synthetic method of MOF-derived carbons [71]. For the fabrication of *X*Cu@Al-MIL-101-NH_2_ (*X* = the weight percent of metal ions in the MOFs), the encapsulation of Cu^2+^ ions was employed to the NH_2_-bdc containing Al-MOF self-sacrificing template via double-solvents method. MOF-derived carbons (MIL-C, MIL-C-X (X = 0.5, 1, and 2)) were prepared from carbonization of the MOF or Cu@MOF composite at 800 °C (5 h, Ar flow), followed by acid treatment. The encapsulated Cu^2+^ ions were greatly influenced by pore generation and the control of porosity. As a result, the increment of encapsulated amount of the Cu^2+^ ions improved hierarchical porosity and V_total_ of the resultant carbons. The development degree of the hierarchical porosity could be seen according to the following sequence: carbon with dominant microporosity (MIL-C), carbons with micro-/mesoporosity (MIL-C-0.5, MIL-C-1), and carbons with hierarchical micro-/meso-/macroporosity (MIL-C-2). Under 2E system with 1 M H_2_SO_4_ electrolyte, the maximum specific capacitances at 10 mV s^−1^ were 145, 143, 180, and 185 F g^−1^ for the carbon series of MIL-C, MIL-C-0.5, MIL-C-1, and MIL-C-2, respectively.

Liu et al. introduced a simple and effective self-sacrificing template synthetic strategy for constructing porous carbon nanosheets (CNs) from Al-based MOF (DUT-5) [72]. The 4,4′-biphenyldicarboxylate (bpdc) containing DUT-5 self-sacrificing template was converted into the pure mesoporous carbon nanosheets by carbonization at 700 °C (3 h, N_2_ flow) and subsequent HCl washing. The obtained CNs exhibited moderately high values of S_BET_ and V_pore_ (0.99 cm^3^ g^−1^). In the 2E system, the maximum value of specific energy was 4.3 W h kg^−1^ at 0.25 A g^−1^. At 3 A g^−1^, the specific energy was minimized to 2.7 W h kg^−1^ and specific power was maximized to 2068 W kg^−1^. High specific power was due to the 2D sheet-shaped structure and well-developed mesoporosity.

#### 2.2.2. Cd-MOF-Derived Carbons

Li et al. revealed two reports about Cd-MOFs-based carbon electrodes [73,74]. The Li’s group first introduced Cd-MOFs as carbon precursors [73]. The four Cd-MOFs were prepared by employment of H_2_bdc linker combined with four structure-interconnected bis(imidazole) linkers (bib = 1,4-bis(imidazol-1-yl)benzene, bbib = 1,4-bis(benzimidazol-1-yl)benzene, bibp = 4,4′-bis(imidazol-1-yl)biphenyl, and bbibp = 4,4′-bis(benzimidazol-1-yl)biphenyl. The Cd-MOF-derived porous carbons (PCs) were synthesized from the carbonization of Cd-MOFs (1000 °C, 12 h, N_2_) and following acid treatment (5 vol% HCl). PC@KOH composites (PC/KOH = 1/4) were transformed into activated porous carbon (APC) via pyrolysis at 800 °C (1 h, N_2_ flow) and subsequent washing (5 vol% HCl and water). The APC-bib possessed the largest S_BET_ and V_total_ (1.37 cm^3^ g^−1^) among the eight samples. Electrochemical performance was investigated in 3E system with 6 M KOH electrolyte. The order of specific capacitances of PCs was PC-bib > PC-bibp > PC-bbib > PC-bbibp at low scan rates and low current loads. It was the same order of S_BET_ controlled by Cd/C ratio of the MOF templates. Moreover, the APCs showed enhanced specific capacitances compared with the PCs due to the increase of conductivity, ionic diffusion, and charge transfer.

They also investigated the role of different solvents for Cd-MOF carbon precursors in the preparation of MOF-derived PCs [74]. Five different solvents (methanol, ethanol, isopropanol, DMF, NMP = N-methyl-2-pyrrolidone) were used to synthesize a series of new Cd-MOFs under the same mixed ligand system (bib and H_2_ipa = isophthalic acid). After carbonization at 1000 °C (12 h, N_2_ flow) and following 2 M HCl washing, the five Cd-MOFs were transformed into the PCs. The resultant PCs possessed relatively low S_BET_. The composites of PC@KOH (PC/KOH = 0.25) were also transformed into activated APCs through carbonization at 800 °C (1 h, N_2_ flow) and followed by washing process (2 M HCl and water). The values of S_BET_ of the APCs (APC-me, APC-eth, APC-ipr, APC-dmf, and APC-nmp) were 1143, 1312, 1074, 1408, and 1337 m^2^ g^−1^, respectively. These textural improvements inducing both microporosity and mesoporosity by KOH activation supported the electrochemical performance differences found in 3E cells with the electrolyte of 6 M KOH. The APCs exhibited improved specific capacitances compared to the PCs because of the enhanced properties of conductance, ionic diffusion, and charge transfer.

#### 2.2.3. Co-MOF-Derived Carbons

**Figure 4:**
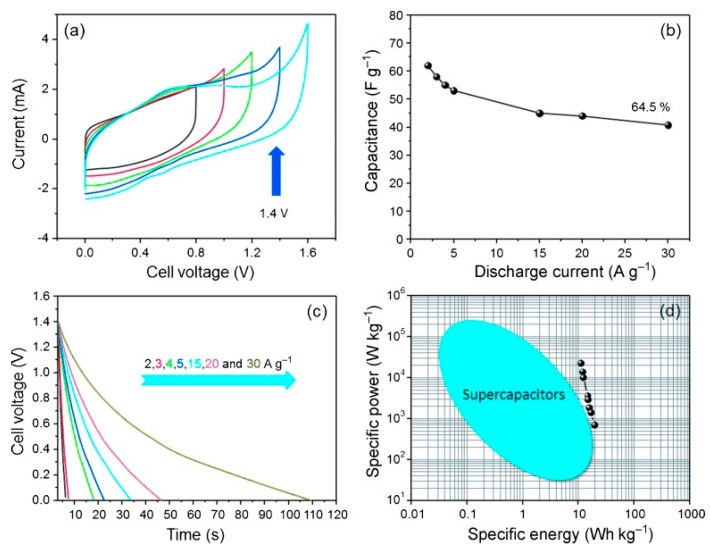
The electrochemical performance of SSC based on NPC-800 using 0.5 M H_2_SO_4_ electrolyte. (**a**) Cyclic voltammograms at 20 mV s^−1^ combined with diverse cell voltages (from 0.8 to 1.6 V). (**b**) Specific capacitances obtained by charge-discharge data with a cell voltage of 1.4 V. (**c**) Galvanostatic discharge curves at different current loads (from 2 to 30 A g^−1^) with 1.4 V cell voltage. (**d**) Ragone plot [75].

Yamauchi et al. reported the ZIF-67 derived nanoporous carbon (NPC) with high degree of graphitization [75]. The direct carbonization of the ZIF-67 at 800 °C (5 h, N_2_ flow) produced Co/NPC composite, and this composite was transformed into the pure nanoporous carbon (NPC-800) via 10% HF etching. The NPC-800 had high S_BET_ with a large V_pore_ (0.84 cm^3^ g^−1^). It also exhibited micro-/mesoporous porosity and ultra-high graphitization degree (*I*_G_/*I*_D_ = 2.07). In the 3E system with 0.5 M H_2_SO_4_ electrolyte, it delivered 238 F g^−1^ at 20 mV s^−1^ and 171 F g^−1^ at 200 mV s^−1^ (Figure 4). Moreover, with an operation cell voltage of 1.4 V, the specific capacitance of 2E cell at 2 A g^−1^ was 62 F g^−1^. High rate performance was observed in both 3E and 2E cells because of the hierarchical porosity and high graphitization degree of NPC-800.

#### 2.2.4. Cu-MOF-Derived Carbons

A Cu-MOF containing 1,4-bis(imidazol-1-yl) benzene (bib) was employed as a self-sacrificing template to prepare hierarchical flower-like N-doped porous carbon materials (NPCs) by Li et al. [76]. The self-sacrificing template possessed a formula of {[Cu(bib)_2_(H_2_O)_2_] (ClO_4_)_2_(H_2_O)}, and its 1D pores contained potentially explosive perchlorate ions. This unique pore structure could enhance the porosity of Cu@C composites during the carbonization of the template (12 h, N_2_ flow). In addition, HCl etching of the composites might induce the additional pore generation. Among the NPCs, NPC-900 (carbonization at 900 °C) showed superior porosity and graphitization to other NPC samples. Therefore, better electrochemical performance was displayed for the NPC-900 with 3E system employing 6 M KOH electrolyte.

New bib-based Cu-MOFs for the synthesis of novel porous carbon nanosheets were prepared by the same group under different solvent system, MeOH and chloroform, and Cu source, Cu(NO_3_)_2_∙3H_2_O [77]. A formula of the Cu-MOF was {[Cu(bib)_2_(NO_3_) (H_2_O)](NO_3_)(H_2_O)_0.5_}. Direct carbonization of the Cu-MOF at 800 °C (3 h, N_2_) induced the formation of Cu@C composite with the preservation of hierarchically layered morphology, and helped Cu-catalyzed graphitization. After the etching process of Cu@C with HCl, the activation of acid-etched Cu@C with KOH (800 °C, 1 h, N_2_ flow, Cu@C/KOH = 1/4) generated APC with hierarchical porosity. The exfoliation of the layered morphology into nanosheet also occurred. Thus, the APC nanosheet showed a high S_BET_ with a large V_pore_ (1.50 cm^3^ g^−1^). In 6 M KOH electrolyte with 3E cell, the specific capacitances of the APC were 231 F g^−1^ at 5 mV s^−1^ and 260.5 F g^−1^ at 0.5 A g^−1^. The APC nanosheet electrode also exhibited good electrochemical properties in symmetric 2E.

#### 2.2.5. Fe-MOF-Derived Carbons

Nonporous iron-based coordination polymer (CP) nanodisks were synthesized and used as carbon precursor for the fabrication of hexagonal-nanodisk shaped mesoporous graphitic carbons [78]. The different iron-based CPs (CP-FeSO_4_ and CP-FeCl_2_) were constructed from constructed by hydrothermal reaction of the NTCDA (1,4,5,8-naphthalenetetracarboxylic dianhydride) with Fe sources (FeSO_4_ or FeCl_2_) under economical and green condition. Catalytic carbonization (4 h, N_2_ flow) of the CPs controlled by carbonization temperature and Fe source could determine the hierarchical porosity and graphitization degree of the carbonized sample. In addition, HCl (2 M) washing of the carbonized sample generated additional mesopore. More porous but less graphitized carbons were produced by lower carbonization temperature (900 °C) and CP-FeSO_4_-based conditions. These conditions could also influence the capacitive property of the carbon samples in 6 M KOH electrolyte with 3E system. At 2 mV s^−1^, the gravimetric specific capacitance values of C-S700, C-S-900, C-Cl700 and C-Cl900 were 182, 156, 117 and 70 F g^−1^, respectively.

Zhuang et al. reported porous carbon derived from MIL-101(Fe) [79]. Mild and green synthetic condition (room temperature, organic solvent-free) of the MOF self-sacrificing template with a cheap metal source (FeSO_4_) in a large scale could meet the needs of industrial and environmental chemistry. Carbonization of MIL-101(Fe) at 800 °C (0.5 h, Ar flow) followed by 2 M HCl treatment generated hollow carbon polyhedra (HCPs). The resultant porous HPCs (V_pore_ = 1.68 cm^3^ g^−1^) showed highly graphitized and ultrathin carbon layer with micro-/mesoporous hierarchical porosity. Therefore, the HPCs were good electrode materials under symmetric 2E system in 6 M KOH electrolyte. The capacitances were high levels for 2E cells, 214 F g^−1^ at 0.05 A g^−1^ and 145 F g^−1^ at 5 A g^−1^.

#### 2.2.6. K-MOF-Derived Carbons

The first example of MOF-derived carbon-based SSCs in a redox electrolyte (KI in H_2_SO_4_) was reported by Fischer and coworkers [80]. As a self-sacrificing template and a precursor for 2D porous carbon nanosheets (NPCs), the K-MOF was prepared from KI and BTC. The formula of the K-MOF was {K_3_{C_6_H_3_(CO_2_) (CO_2_H_0.5_)(CO_2_H)}_2_}(H_2_O)_2_. After two-step pyrolysis under N_2_ (450 °C for 6 h and 800 °C for 8 h) followed by HCl (5 wt.%) treatment, the activated MOF nanorods was successfully transformed into 2D graphitic nanoporous carbon sheets (NPS-800). The obtained NPS-800 with high specific surface area had hierarchical porosity as well as large V_total_ (1.06 cm^3^ g^−1^). Electrochemical properties of SSC based on the NPS-800 electrode were investigated in 1 M H_2_SO_4_ electrolyte with/without addition of redox-active KI. The supercapacitor exhibited outstanding value of BET-surface–normalized capacitance (21.4 μF cm^−2^) at 5 mV s^−1^. In addition, the cell showed large values of gravimetric and volumetric energy densities, 24.8 W h kg^−1^ and 14.93 mW h cm^−3^, at 533 W kg^−1^ (320 mW cm^−3^) power density. Furthermore, great improvements of electrochemical properties of the symmetric cell could be seen after addition of 0.2 M KI to 1 M H_2_SO_4_ electrolyte. From CV data, significantly increased gravimetric and areal capacitances were observed, 1636 F g^−1^ and 137.3 μF cm^−2^. It also delivered remarkably enhanced values of volumetric specific capacitance (151 F cm^−3^) and energy/power density (89.7 W h kg^−1^, 53.8 W h cm^−3^) based on GCD cycles. Thus, both battery grade energy density and capacitor grade power density were achieved by this NPC-based cell.

#### 2.2.7. Mg-MOF-Derived Carbons

From using the inexpensive ligands (bdc or btc) and Mg salts, four Mg-MOFs were solvothermally synthesized with/without polyethylene terephthalate (PET) inducer for tuning the quantity of MgCl_2_ impurity [81]. The Mg-MOF/MgCl_2_ hybrid products had different morphologies, and they were used as carbon precursors. They were transformed into the carbons via carbonization at 800 °C (5 h, N_2_ flow) and HCl washing. Addition of the inducer decreased the MgCl_2_ impurity of the hybrid products. Therefore, the resultant carbon samples (CP-II and CP-III) contained higher values of S_BET_ and specific capacitance than those of carbons derived from PET-free precursors (C-II and C-III). Electrochemical properties of the carbon samples were studied in 6 M KOH electrolyte with 3E cell. The CP-II carbon derived from btc-based Mg-MOF exhibited the highest specific capacitance of 127 F g^−1^ at 1 A g^−1^. On the other hand, the CP-III carbon (with laminated structure) derived from bdc-based Mg-MOF showed a better capacitive rate capability (80%) with slightly smaller specific capacitance of 121 F g^−1^ at 1 A g^−1^.

#### 2.2.8. Ni-MOF-Derived Carbons

Fu et al. reported N-doped porous graphitic carbon (NPGC) from a combined synthetic route of coordination-pyrolysis [82]. Precursor of the NPGC was prepared from coordinative assembly of porogen (tetraethyl orthosilicate or TEOS), graphitic catalyst source (nickel nitrate), carbon precursor (glucose), and nitrogen source (melamine) by a low-temperature reaction (80 °C, 4 h). After carbonization at 900 °C (1 h), SiO_2_-Ni-N-doped graphitic carbon was prepared. The SNGC-2-900 (from 2 g of melamine) was transformed into NPGC-2-900 via 10 wt.% HF solution treatment. The nanosheet-like NPGC-2-900 showed a large S_BET_ (1027 m^2^ g^−1^), interconnected porosity (micro-/mesopore, D_pore_ = 5 nm), a high N-dopant content (7.72 wt.%), and a high graphitic degree (*I*_G_/*I*_D_ = 2.33). These properties were adequate for electrochemical energy storage in both 6 M KOH and 1 M Et_4_NBF_4_/PC electrolytes. When the NPGC-2-900 was applied to 3E cell with 6 M KOH, it showed 293 F g^−1^ at 1 A g^−1^ and 184 F g^−1^ at 30 A g^−1^. For 2E cell system, its total-cell specific capacitances at 1 A g^−1^ were 73 F g^−1^ in 6 M KOH and 47 F g^−1^ in 1 M Et_4_NBF_4_/PC.

#### 2.2.9. Sr-MOF-Derived Carbons

**Figure 5:**
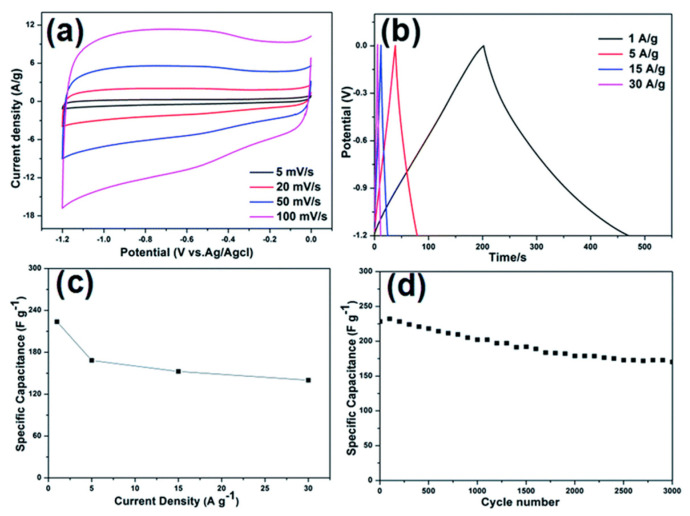
The electrochemical property of N-C-650-based supercapacitor for 3E system in 6 M KOH electrolyte. (**a**) CV curves at various scan rates. (**b**) GCD curves at various current densities. (**c**) Specific capacitances calculated from the GCD curves. (**d**) Cyclic stability at 1 A g^−1^ for 3000 cycles [83].

There was a rare example of N-doped carbon materials derived from Sr-MOF. 7,7,8,8-tetracyanoquinodimethane (TCNQ) was chosen as a N-rich linker to synthesize N-doping Sr-MOF self-sacrificing template and its corresponding N-doped porous carbons [83]. Temperature-controlled carbonization under N_2_ atmosphere of the Sr-MOF self-sacrificing template and subsequent Sr species removal by c-HCl etching could generate the N-doped carbon series (N-C-450, N-C-550, and N-C-650). Interestingly, the carbonization temperature-driven adjustment of heteroatom (N and O) contents strongly influenced capacitive properties of the carbon series. As a result, N-C-650, carbonized at 650 °C, was the best working electrode material for 3E system under 6 M KOH electrolytic conditions (Figure 5).

#### 2.2.10. Zr-MOF-Derived Carbons

Nanoporous carbons (NCs) with controlled particle size and morphology were efficiently prepared from MOF-525 nanocrystals [84]. The porphyrin-based Zr-MOFs were synthesized from ZrCl_2_ and porphyrin ligand (5,10,15,20-tetrakis(4-carboxyphenyl) porphyrin) under different quantitative addition of benzoic acid modulator. The size of the cubic MOF was proportional to the amount of the modulator, and it also determined the size of resultant NCs. Direct carbonization at 800 °C (1 h, N_2_ flow) and subsequent HF (10 wt.%) treatment of the Zr-MOF series (MOF0.9, MOF1.1, MOF1.35, MOF2.0, and MOF2.7) produced a series of corresponding star-shaped NCs (NC0.9, NC1.1, NC1.35, NC2.0, and NC2.7). The porous NC1.35 electrode for capacitor was operable in 1 M H_2_SO_4_ electrolyte with 3E configuration. It exhibited high specific capacitance of 425 F g^−1^ at 2 A g^−1^. This excellent performance was mainly due to the combination of the large S_BET_ and hierarchical pore structure. Enhanced surface wettability and pseudocapacitance generated from N-/O-functionalities could also contribute to the specific capacitance.

Gan et al. pioneered a facile and effective synthetic approach for MOF-derived ultra-microporous carbon NPs (UCNs) [85]. MOFs of UiO-67 nanocrystals played the key dual roles as a sacrificial template and a carbon precursor for the UCNs preparation. The Zr-based MOF was synthesized form ZrCl_2_ and H_2_bpdc with benzoic acid modulator. The UiO_67_-x derived UCN-x-y (x = modulator equivalent based on the ZrCl_2_, y = carbonization temperature) was constructed from carbonization (3 h, N_2_ flow) of the Zr-MOF crystals and HF treatment. Both the amount of modulator and temperature of carbonization could determine morphological and porous properties of the UCNs. As the optimal condition, UCN-20-750 achieved balance between specific surface area and conductivity. Nanoscale UCN-20-750 particles had high S_BET_ together with narrow distribution of micropore (0.53 nm). As an electrode material for supercapacitor working in 3E cell under 6 M KOH electrolytic condition, it showed a high value of specific capacitance.

### 2.3. Other Kinds

There exist two reports about ZnBi-MOF-derived carbon which were published by Yuan et al. The carbons were synthesized via single-step carbonization at 1000 °C (8 h, N_2_ flow) [86,87]. In the first example, worm-like mesoporous carbon (WMC) was derived from glycerol/MOCP (MOCP = metal–organic coordination polymer) composites [86]. The self-sacrificing template of bdc-based ZnBi-MOCP was combined with a secondary carbon source of glycerol to construct the composites. An environmentally friendly and simple direct carbonization of the composites generated the NMC, and the obtained mesoporous carbon exhibited high S_BET_ and a large V_pore_ (3.14 cm^3^ g*^−^*^1^). High maximum specific capacitance of 344 F g*^−^*^1^ at 50 mA g*^−^*^1^ in 6 M KOH as well as high rate capability behavior of 109 F g*^−^*^1^ at 2 A g*^−^*^1^ was supported by low ohmic property resulting from micro-/mesoporous hierarchical porosity. Yuan et al. investigated the synergistic effect of the amount of Bi (NO_3_)_3_∙5H_2_O and the volume of glycerol on porosity of the resultant series of hierarchical porous carbon (HPC) [87]. HPCs-0.4 sample had the highest S_BET_ and V_pore_ of 3.9 cm^3^ g*^−^*^1^ among the series. The specific capacitance of HPCs-0.4 reached up to 241 F g*^−^*^1^ at 0.1 A g*^−^*^1^. This performance could also be ascribed to hierarchical porosity especially micro- and mesopore regions.

MOF-derived carbons (MCs) were obtained from the direct carbonization at 800 °C (5 h, Ar flow) of three MOFs (HKUST-1, MOF-5 and Al-porous carbon polyhedrons (PCP)) and further HF treatment [88]. All the carbon samples had mesoporosity. In addition, the S_BET_ of the corresponding MC-Cu, MC-Zn and MC-Al samples were 50, 420, and 1103 m^2^ g*^−^*^1^. Among them, the Al-PCP derived carbon (MC-Al) possessed the highest degree of graphitization and the most suitable hierarchical pore structure. Therefore, the high S_BET_ and hierarchical porosity of the MC-Al might have synergistic effect on the best electrochemical properties under 3E cell in 30% KOH electrolyte. At 100 mA g*^−^*^1^, the maximum specific capacitances of the MC-Cu, MC-Zn, and MC-Al carbons were 142.3, 121.3, and 232.8 F g*^−^*^1^, respectively.

Li et al. investigated the role of metal ion in MOF self-sacrificing templates during the synthesis of MOF-derived carbon using six isomorphous MOFs in 2018 [89]. The 3D window-beam structured MOFs had a formula with {[M_3_(btc)_2_(bibp)_2_(H_2_O)_2_]∙3H_2_O} (M = Zn^II^, Cd^II^, Ni^II^, Mn^II^, Co^II^, and Cu^II^). In the cases of Zn- and Cd-MOFs, a single-step carbonization at 1000 °C (3 h, N_2_ flow) was required to synthesize carbon products known as PC-Zn and PC-Cd, respectively. However, in other cases, additional HCl etching was performed to prepare pure carbonaceous products (GC-Ni, GC-Mn, GC-Co, and C-Cu). From the carbon precursors with same morphology, the resultant carbons with different types were produced and they were categorized into three types: porous carbons (PCs), graphitized carbons (GCs), and C-Cu. The PCs exhibited pore generation effect by Zn or Cd vapor during the carbonization process, so the values of S_BET_ were relatively high, 1558 m^2^ g*^−^*^1^ for PC-Zn and 1430 m^2^ g*^−^*^1^ for PC-Zn. In the case of Ni, Co, and Mn-MOFs, the GCs with relatively small S_BET_ were formed by metal species induced catalytic graphitization of carbon matrix. In addition, C-Cu with no graphitization showed the lowest S_BET_ with no decent microporosity. The specific capacitances from 3E cell in 6 M KOH electrolyte decreased in the order of PCs > GCs > C-Cu. Therefore, the S_BET_ was thought to be a key factor determining the electrochemical properties by controlling electrochemically accessible surface area and ionic diffusion.

### 2.4. Asymmetic Supercapacitor (ASC)

Table 5 depicts the electrochemical performances of asymmetric supercapacitors (ASCs) made of pure and heteroatom-doped carbons derived from MOFs and other electrode materials.

MOF-derived carbon (MOF-DC) was first employed as cathode electrode material for Li-ion hybrid electrochemical capacitors (Li-HECs) [90]. The MOF-DC was facilely synthesized from a single-step carbonization of MOF-5 at 1000 °C (8 h, Ar flow). The MOF-DC with 3D cuboid structure showed ultra-high S_BET_, hierarchical assembly of crumpled sheet, and hierarchical porosity (micro- and mesopores). After control of mass ratio (anode: cathode = 1:2), the Li-HEC of MOF-DC/Li_4_Ti_5_O_12_ was operated in 1 M LIPF_6_ in EC-DMC (ethylene carbonate–dimethyl carbonate) electrolyte. The Li-HEC could deliver a high energy density about 65 W h kg^−1^ with good power performance. This value was superior to symmetric cell assembled from the MOF-DC electrode (~20 W h kg^−1^). Another Li-HEC was reported by Huo et al. from ZIF-8-derived carbon cathode material [91]. After carbonization of the ZIF-8 at 800 °C followed by HCl treatment, MOF-derived N-doped carbon hollow polyhedrons (MOF-NC) were synthesized. The MOF-NC had high S_BET_ (S_micro_ = 496.3 m^2^ g^−1^, S_meso_ = 974.6 m^2^ g^−1^). As a working electrode of the 3E cell in 1 M LiPF_6_ in EC/DMC, it showed a capacitance of 269.9 F g^−1^ at 0.2 A g^−1^. In addition, at 6 A g^−1^, it yielded good rate performance (63% retention). WO_3_/C//MOF-NC Li-HEC was also operable in the same electrolyte with a controlled mass ratio of WO_3_/C : MOF-NC = 1 : 7.6. At 0.5 A g^−1^, the Li-HEC exhibited the highest values of discharging cell capacitance (184.3 F g^−1^) and energy density (159.97 W h kg^−1^) with a power density of 172.6 W kg^−1^.

ZIF-8-derived PCPs were first used as a negative electrode for ASCs operating in an aqueous electrolyte [92]. The PCPs were synthesized form two-step pyrolysis of the ZIF-8 polyhedrons (150 °C for 2 h and 1000 °C for 8 h in N_2_). The well-shaped PCPs with plentiful micropores (~1.8 nm) and mesopores (~3.6 nm) showed high S_BET_ of 1980 m^2^ g^−1^ as well as large V_total_ of 1.13 cm^3^ g^−1^. Under 1 M KOH electrolyte with 3E cell, carbon electrode based on the PCPs with hierarchical porosity yielded capacitances of 245 F g^−1^ at 1 A g^−1^ and 162 F g^−1^ at 20 A g^−1^. After the optimization of mass ratio (CNT@NiO: PCPs = 0.5), the ASC of CNT@NiO//PCPs behaved well in the same electrolyte. In the ASC, the CNT@NiO was a battery-like faradaic positive electrode for energy source, while the PCP was a capacitive negative electrode for power source. At a current density of 0.5 A g^−1^, the ASC had maximum values (capacitance = 72 F g^−1^, energy density = 25.4 W h kg^−1^) with a specific power of 400 W kg^−1^. A new solid-phase hybrid supercapacitor (HSC) of Mn_2_O_3_@NF//MC was reported [93]. The MC with mesoporous structure was prepared by direct carbonization at 800 °C (4 h, N_2_ flow) of ZIF-8 polyhedra and subsequent 2 M HCl washing. Its S_BET_ was 365 m^2^ g^−1^. With the proper mass ratio of MC to Mn_2_O_3_ (1.033), the HSC was assembled from binder-free positive electrode (Mn_2_O_3_@NF) and MC negative electrode in PVA/LiCl electrolyte. At a current density of 0.4 A g^−1^, the solid-phase HSC yielded the highest values of capacitance (317.5 F g^−1^) and energy density (112.82 W h kg^−1^) with a power density of 320 W kg^−1^.

The flexible ASC of Zn-Co-O@CC//NPC@CC was reported [94]. The NPC was synthesized via carbonization of 2-methylimidazole-based Zn-Co-MOF and followed by HCl treatment. The all-solid-phase ASC was assembled from binder-free electrodes using flexible carbon cloth (CC) substrate and PVA/LiCl electrolyte. At a current density of 1.5 A g^−1^, it yielded maximum capacitance of 210 F g^−1^ and energy density of 117.92 W h kg^−1^ with delivering power density of 1490.4 W kg^−1^. In addition, at 20 A g^−1^, it showed the highest power density of 13.52 kW kg^−1^ with high rate performance (capacitance = 138.75 F g^−1^, energy density = 76.69 W h kg^−1^). Moreover, it had great structural stabilities (84.52% preservation after 1000 bending cycles with 180° bending angle, 89.41% preservation after 6000 cycles with different bending states).

The 2-methylimidazole-based Zn-Co-MOF was used for the fabrication of NPC@CFP (CFP = carbon fiber paper) electrode for a flexible ASC of Ni-Co-O@CFP//NPC@CFP [95]. The bimetallic MOF was directly grown on CFP support. Then, the NPC@CFP was synthesized by carbonization at 800 °C (2 h, N_2_ flow) of the NPC@CFP. The NPC@CFP anode exhibited a high specific capacitance (265 F g^−1^ at 1 A g^−1^) in 1 M KOH. The ASC showed a high specific capacitance (201 F g^−1^ at 0.55 A g^−1^) with excellent rate performance (69 W h kg^−1^ at 840 W kg^−1^ and 58 W h kg^−1^ at 2985 W kg^−1^) in KOH/PVA hydrogel electrolyte.

A 2D ZIF-67 was first used to single-templated synthesis of electrode materials (both anode and cathode) for flexible solid-phase ASC [96]. At first, the direct growth of Co-based ZIF-67 on conducting CC was performed to form flexible precursor of CC@Co-MOF. A cathode of CC@Co_3_O_4_ nanosheet was prepared by two-step pyrolysis (500 °C, 1 h, N_2_ flow and 350 °C, 2 h, air flow) of the precursor. However, the precursor was transformed into an anode of CC@N-doped carbon nanosheet (CC@NC) via carbonization at 800 °C (5 h, N_2_) and subsequent 1 M FeCl_3_ treatment. In the 3E cell with 2 M KOH electrolyte, the CC@NC yielded high capacitance of 321.9 F g^−1^ at 6.25 A g^−1^. The ASC of CC@Co_3_O_4_//CC@NC was fabricated in PVA/KOH electrolyte. This device also exhibited a maximum capacitance of ~116.8 F g^−1^ at 7.7 A g^−1^.

Another self-templating synthetic approach from ZIF-67 was applied to flexible supercapacitor of CC@NiCo_2_O_4_//CC@NC [97]. The raw precursor of CC@Co-MOF was converted into CC@Ni-Co LDH (LDH = layered double-hydroxide) by Ni (NO_3_)_2_-driven ion-exchange and etching. The cathode, CC@NiCo_2_O_4_, was synthesized via a one-step pyrolysis of the CC@Ni-Co LDH at 350 °C (2 h, air flow). In addition, the anode, CC@NC, was prepared from carbonization at 800 °C (2 h, Ar) and following 3 M HCl treatment. The CC@NiCo_2_O_4_//CC@NC cell was operated in PVA/KOH electrolyte. At 5 mA cm^−2^, it yielded maximum values of capacitance (~89.7 F g^−1^) and energy density (31.9 W h kg^−1^) with a power density of 2.9 kW kg^−1^. In addition, at 40 mA cm^−2^, it exhibited maximum power density of 22.9 kW kg^−1^ with ultra-high rate performance of capacitance (~76 F g^−1^) and energy density (27.3 W h kg^−1^). Other types of ASC were constructed from another self-sacrificing template of Ni-DMOF-TM ([Ni(tmbdc)(dabco)_0.5_], tmbdc = 2,3,5,6-tetramethyl-1,4-benzenedicarboxylic acid) [98]. A new battery-like positive electrode was CFP@TM-nanorods, synthesized from two-step thermal growth (85 °C for 24 h and 120 °C for 24 h) of the binder-free TM-nanorods on conducting carbon fiber paper (CFP) with the assistance of triethylamine. This positive electrode was transformed into negative electrode of CFP@TM-NPCs (NPCs = N-doped hierarchical porous carbon nanorods) via carbonization at 1000 °C (3 h, Ar flow) followed by HCl treatment. The TM-NPCs contained large S_BET_ of 998 m^2^ g^−1^ as well as hierarchical porosity. Under 3E mode with 2 M KOH electrolyte, the TM-NPC had capacitances of 330 F g^−1^ at 1 A g^−1^ and 290 F g^−1^ at 20 A g^−1^. With the optimization of mass ratio of TM-nanorods to TM-NPCs (1:3.6), the ASC of TM-nanorods//TM-NPCs was operated in the same electrolyte. At 1 A g^−1^, high capacitance (161 F g^−1^) and energy density (47.1 W h kg^−1^) could be found with a power density of 1018 W kg^−1^.

## 3. Metallic NP-Containing Carbons

The metallic NPs as well as heteroatom dopants in carbon matrices can enhance the capacitive properties by good electrical conductivity, pseudocapacitance, and favorable wetting of electrode surfaces. The electrochemical performances of metallic NP-containing carbons are summarized in Table 6.

### 3.1. Ni-MOF-Derived Carbons

#### 3.1.1. Ni-bdc MOF-Derived Carbons

**Figure 6:**
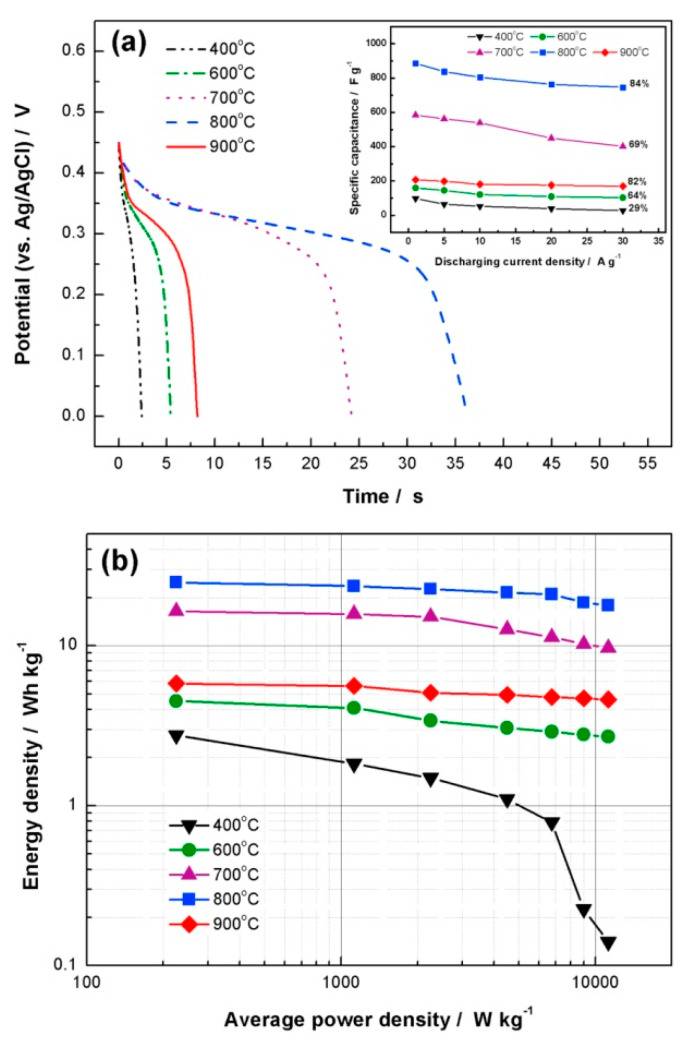
Electrochemical behaviors of working electrodes based on carbonized Ni-BDC for 3E setup using 1 M KOH electrolyte. (**a**) Discharge curves at 10 A g^−1^ and influence of discharge current load on the specific capacitance (inset). (**b**) Ragone plots [99].

Ni-bdc MOF was introduced as a carbon precursor for supercapacitor [99]. After calcination of the Ni-bdc at 400 °C in air, Ni particles embedded porous carbons were prepared. After further carbonization at 800 °C under N_2_ atmosphere, the resultant C800MOF showed optimized porosities. Electronic conductivity (from graphitic structure), ionic transport (from large mesopores and macropores), and pseudocapacitance (from redox reactions by uniformly dispersed Ni particles: Ni(OH)_2_ + OH^−^ ↔ NiOOH + H_2_O + e^−^, NiO + OH^−^ ↔ NiOOH + e^−^) synergistically supported good capacitive properties of the C800MOF-based working electrode in 3E cell under 1 M KOH electrolyte (Figure 6). It showed 886 F g^−1^ at 1 A g^−1^ and 746 F g^−1^ at 30 A g^−1^. High rate capability could be seen in energy density (from 25 to 18 W h kg^−1^) when the power density increases from 225 W kg^−1^ to 11,250 W kg^−1^.

Ni-bdc was also unprecedentedly used as a self-sacrificing template for in situ growth of CNT [100]. Without additional materials such as secondary carbon source, catalyst, and reducing agent, the single sacrificial template was directly carbonized at 800 °C (8 h, N_2_ flow) and then treated with HNO_3_. The resultant CNT was first employed as an electrode material for all-solid-phase SSC using PVA/KOH gel electrolyte. High areal specific capacitance with good capacitive rate capability could be obtained (272 mF cm*^−^*^2^ at 0.5 mA cm*^−^*^2^ and 168 mF cm*^−^*^2^ at 10 mA cm*^−^*^2^). Its highest energy density and power density were 0.0544 mW h cm*^−^*^2^ and 5.988 mW cm*^−^*^2^, respectively. These good properties might result from high S_BET_, uniform porosity, and high electronic conductivity from graphitized carbon matrix and a few residual Ni species.

#### 3.1.2. Ni-btc MOF-Derived Carbons

Yoon et al. reported Ni-btc-derived hierarchical porous Nickel@Carbon nanospheres (HP-Ni@C-N) [101]. During the shape-preserved simple carbonization at 400 °C (2 h, N_2_ flow), individual core-shell structure was constructed with graphitized carbon layers which encapsulated nickel NPs. The resultant HP-Ni@C-N exhibited good mesoporosity (main V_pore_ = 13.27 nm). Compared to the Ni-btc MOF, the HP-Ni@C-N showed better capacitive performance in 3 M KOH electrolyte in 3E cell system (Ni-MOF: 493 F g*^−^*^1^ at 10 mV s*^−^*^1^ and 221 F g*^−^*^1^ at 100 mV s*^−^*^1^, HP-Ni@C-N: 912 F g*^−^*^1^ at 10 mV s*^−^*^1^ and 397 F g*^−^*^1^ at 100 mV s*^−^*^1^). Porous structure with high S_BET_ as well as electronic conductivity improved by core-shell structure might have synergistic effects on these superior properties.

#### 3.1.3. Other Ni-MOF-Derived Carbons

The carbonization of nanorod-shaped hhtp-based Ni-CAT MOF (hhtp = 2,3,6,7,10,11-hexahydroxytriphenylene, CAT = catecholate) and subsequent 2 M HCl etching were conducted to prepare nanoporous carbon nanorod (CNRods) showing micro-/mesoporosity [102]. The carbonization was performed at 700, 800, and 900 °C (2 h, N_2_ flow). The particle morphology was preserved during carbonization. After suitable control of O-dopant contents and Ni-catalyzed graphitization degree during the carbonization, CNRod_800_ (carbonized at 800 °C, S_BET_ = 367 m^2^ g*^−^*^1^) showed improved electrochemical performance in 3E cell in 6 M KOH due to good ionic and electronic conductivities as well as wettability. At 0.5 A g*^−^*^1^, it had a gravimetric specific capacitance of 127 F g*^−^*^1^ and normalized areal specific capacitance of 34.6 μF cm*^−^*^2^.

A unique Ni-MOF with multi-component dopant sources, such as P, N, and O, was synthesized and used as a self-sacrificing template for the preparation of uncommon carbon materials (Ni/P/N/C-T, T = carbonization temperature in °C). The resulting Ni/P/N/C-T carbons showed uniformly incorporated aforementioned multi-component dopants and Ni element (Ni, P, N, and O) [103]. The Ni-MOF with a formula of [Ni_3_(H_2_O) (bpy)_3_(L1)] was prepared from the mixed ligand system of bpy and hexakis(4-formylphenoxy)cyclotriphosphazene (H_6_L1). Then, it was transformed into the Ni/P/N/C-T carbons through single-step carbonization at varying temperatures. During the carbonization, many properties influencing electrochemical performance, such as hierarchical porosity, dopant distribution, wettability, and conductivity, were regulated. The Ni/P/N/C-500 was found to be the best electrode material for capacitor in 6 M KOH with 3E cell configuration.

### 3.2. Co-MOF-Derived Carbons

#### 3.2.1. ZIF-67-Derived Carbons

Wei et al. introduced a single-step carbonization of ZIF-67 at 1000 °C (5 h, N_2_ flow) to produce metallic Co-containing carbon electrode without additional purification steps [104]. The resultant carbon (S_BET_ = 236 m^2^ g*^−^*^1^) had well-interconnected structure. The metallic Co particles (34.05 wt.%) were crucial centers for electronic transport and pseudocapacitive faradaic reaction in 1 M KOH electrolyte (Co (OH)_2_ + 2e^-^ ↔ Co + 2OH^‾^). As a result, the obtained carbon electrode under 3E cell showed capacitances of 144.5 F g*^−^*^1^ at 0.5 A g*^−^*^1^. They also reported binder-free and Co-containing carbon electrodes on Ni foam conducting substate [105]. The uniformly and closely packed ZIF-67 membrane precursor on the Ni foam was prepared for the synthesis of Co/carbon on the conducting substrate. The resultant Ni foam/ZIF-67 was directly carbonized for 5 h to produce the Co/carbon-based electrodes. The optimized carbon obtained at 800 °C with a ladder-like morphological surface formed thin membrane (thickness ≈ 1.5 μm). In addition, Co NPs on the carbon surfaces showed well-defined size (diameter ≈ 2.6 nm) and homogeneous distribution with no evident agglomeration. Thus, the facilely synthesized working electrode of 3E configuration with 1 M KOH electrolyte exhibited mainly pseudocapacitive behavior and good conductivity. It delivered a remarkably high capacitance of 512.0 F g*^−^*^1^ at 1 A g*^−^*^1^ and 274.3 F g*^−^*^1^ at 20 A g*^−^*^1^.

**Figure 7:**
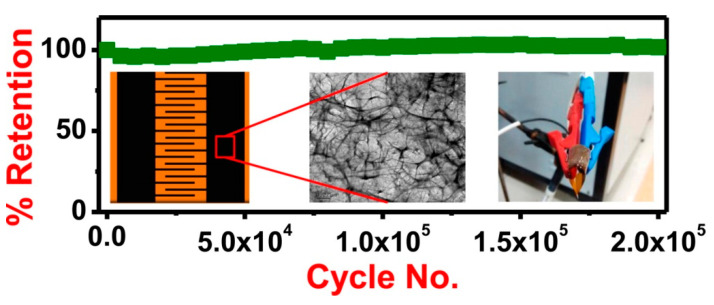
Graphical summary of structural and electrochemical properties for LIMDG electrode [106].

Ogale et al. employed laser carbonization method under ambient conditions to synthesize carbon electrode for a flexible interdigitated micro-supercapacitor (Figure 7) [106]. The new term of LIMDG represents laser-induced MOF-derived graphene. The LIMDG had interconnected network along with curled edges containing few layered graphenes. The hierarchically micro-/mesoporous LIMDG exhibited the S_BET_ of 440 m^2^ g^−1^. This carbon-based symmetric device was operable with PVA/H_2_SO_4_ gel electrolyte. The device displayed great cycling stability (~100% capacitive retention up to 200,000 cycles) and mechanical durability (98% capacitive retention at bending angle of 150°). Therefore, the LIMDG electrode can be potentially applied to flexible electronic devices.

#### 3.2.2. Other Kinds

Co and N co-doped mesoporous carbons were synthesized from the carbonization of N-heterocyclic ligands containing four Co-coordination compounds [107]. Different carbonization temperatures (700 °C or 900 °C, 2 h, N_2_ flow) and subsequent 0.5 M H_2_SO_4_ etching process were applied. Increasing the carbonization temperature generated reduced S_BET_ in the carbons in the absence of acid treatment. Meanwhile, after the acid etching, they increased to 307–462 m^2^ g*^−^*^1^. This result could be explained by the increase of the S_meso_, from 78–152 to 202–290 m^2^ g*^−^*^1^, during the metallic species dissolution caused by the acid etching. Electrochemical measurements of the 3E system were conducted in 6 M KOH and 0.5 M H_2_SO_4_ electrolytes. Acid-treated carbon sample derived from Co (CO_2_) pz at 700 °C had a specific capacitance of 330 F g*^−^*^1^ at 1 A g*^−1^.* The acid-treated carbon sample derived from Co (CO*_2_*) pz at 900 °C showed improved capacitance of 430 F g*^−^*^1^ at 0.5 A g*^−^*^1^ in a mixed electrolyte of 1 M K_4_Fe (CN)_6_ and 3 M KOH. From the parameters of S_meso_ and the Co content (%Co), the following relationship equation about specific capacitance was proposed, C(simulated) = s(S_meso_) + c (% Co). It can be inferred that the overall specific capacitance is the sum of pseudocapacitance contribution (% Co) and EDLC contribution (S_meso_). In 2E system with the Co(CO_2_)pz at 700 °C with acid etching in 6 M KOH electrolyte, the specific capacitance reached to 33.5 F g*^−^*^1^ and the specific energy was 9.1 W h kg*^−^*^1^ at 0.1 A g*^−^*^1^. At 10 A g*^−^*^1^, its maximum specific power was 7 kW kg*^−^*^1^.

### 3.3. Fe-MOF-Derived Carbons

A commercially available Fe-btc MOF (Basolite F300) was employed as a carbon precursor for the hierarchically porous CNSs with hollow nanostructure [108]. Carbonization of the precursor in Ar and following EtOH/HCl (1:5 *v*/*v*) treatment of the carbonized sample produced the CNSs. The hierarchical porosity of the CNSs was composed of micropores in their graphitic layered shell and mesopores in their inner voids. Among the three CNSs (CNS-700, CNS-800, CNS-900), the CNS-800 had optimized property from good balance between graphitization degree and accessible surface area. CNS-800-based SSC operating in 1 M H_2_SO_4_ electrolyte yielded capacitances of 91 F g*^−^*^1^ at 200 mV s*^−^*^1^ and 72 F g*^−^*^1^ at 500 mV s*^−^*^1^. When the electrolyte was replaced with 1-ethyl-3-methylimidazolium tetrafluoroborate (EMIBF_4_), it delivered capacitances of 156 F g*^−^*^1^ at 2 mV s*^−^*^1^ and 88 F g*^−^*^1^ at 100 mV s*^−^*^1^.

### 3.4. Other Kinds

Ni-Co-MOF-74 was used as a raw precursor for the preparation of carbon-based supercapacitor electrode materials [109]. The spherical precursor was solvothermally synthesized from bimetallic salts with 2,5-dihydroxyterephthalic acid under PVP modulator. After a simple single-step carbonization at 800 °C (2 h, N_2_ flow), NC-800 was obtained. Because bimetallic system-driven redox reaction was more effective than monometallic Co-MOF-74 derived C-800, improved specific capacitances were observed in the NC-800 with 6 M KOH electrolytic 3E cell. The NC-800 exhibited fairly high capacitances of 1069 F g*^−^*^1^ at 0.5 A g*^−^*^1^ and 566 F g*^−^*^1^ at 5 A g*^−^*^1^. These good results of the NPC-800 could be explained by electric conductivity from bimetallic system and graphitic carbon, electrolytic diffusion from mesoporosity, and large electrochemically active surface area. The similar single-step carbonization (600 °C, 3 h, N_2_ flow) was applied to the pseudo-bimetallic MOF (nickelocene filled ZIF-67) [110]. The nickelocene@ZIF-67-derived carbon contained dispersed small Ni-Co core particles. In addition, it showed the S_BET_ of 237.6 m^2^ g*^−^*^1^ with well-defined mesoporosity (D_pore_ = 4.2 nm). As a working electrode material of supercapacitor operating in 6 M KOH electrolytic 3E mode, the resultant carbon showed a capacitance of 236 F g*^−^*^1^ at 1 A g*^−^*^1^. Synergistic heterostructure of Co (Co + 2OH^−^ ↔ Co (OH)_2_ + 2e^−^) and Ni (Ni + 2OH^−^ ↔ Ni(OH)_2_ + 2e^−^) with highly mesoporous architecture could result in good electrochemical performance.

### 3.5. Asymmetic Supercapacitor (ASC)

The Co-MOF, ZIF-67, was used for the efficient fabrication of ASCs [111]. The positive electrode of uniform 3D nanoporous Co_3_O_4_ was synthesized from two-step thermolysis (500 °C, 30 min, N_2_ and 350 °C, 2 h, air). The negative electrode of 3D Co-doped NPC (S_BET_ = 350 m^2^ g*^−^*^1^) was prepared by carbonization at 800 °C (5 h, N_2_ flow) followed by HF treatment. As a working electrode of 3E cell in 6 M KOH electrolyte, the capacitances of NPC were moderately good levels of 272 F g*^−^*^1^ at 5 mV s*^−^*^1^ and 163 F g*^−^*^1^ at 200 mV s*^−^*^1^. In the same electrolyte, the ASC of Co_3_O_4_//NPC yielded capacitances of 101 F g*^−^*^1^ at 2 A g*^−^*^1^ and 43 F g*^−^*^1^ at 10 A g*^−^*^1^. Interestingly, the Co_3_O_4_//NPC cell showed a much higher value of maximum specific energy of 36 W h kg*^−^*^1^ at 2 A g*^−^*^1^ compared to the NPC//NPC (7.1 W h kg*^−^*^1^) and Co_3_O_4_//Co_3_O_4_ (7.9 W h kg*^−^*^1^) cells.

The Ni-MOF, [Ni(bdc)(ted)_0.5_∙2H_2_O] (ted = triethyldiamine), was employed as a precursor for the cathode carbon material (Ni/N-doped PC-500) of ASC [112]. After a single-step carbonization at 500 °C of the precursor (2 h, N_2_ flow), the Ni/N-doped PC-500 (S_BET_ = 112 m^2^ g^−1^, D_pore_ = 2.7 nm) with graphitic carbon walls was successfully synthesized. In the basic electrolyte of 6 M KOH, Ni NPs on its surface could be effectively transformed into NiO or Ni(OH)_2_, and these two Ni species showed pseudocapacitive faradaic reactions (NiO + OH^−^ ↔ NiOOH + e^−^, Ni(OH)_2_ + OH^−^ ↔ NiOOH + H_2_O + e^−^). Therefore, it showed very large capacitances of 2002.6 F g^−1^ at 1 A g^−1^ and 534.8 F g^−1^ at 20 A g^−1^ in 3E cell. Combined with AC negative electrode, the Ni/N-doped PC-500 cathode was use for the assembly of ASC (mass ratio of Ni/N-doped PC-500 to AC ≈ 0.3:1). The ASC delivered a capacitance of 115.7 F g^−1^ and an energy density of 41.14 W h kg^−1^ at 1 A g^−1^ with a power density of 800 W kg^−1^.

## 4. Carbon-Based Composites

### 4.1. Carbon/Carbon Composites

#### 4.1.1. Zn-MOF-Derived Composites

##### MOF-5-Derived Composites

The rGO/CMOF-5 (rGO = reduced graphene oxide) composite was easily synthesized from the carbonization of GO/MOF-5 composite at 1000 °C (3 h, Ar flow) [113]. The mesoporous (~3.6 nm) rGO/CMOF-5 showed high S_BET_. In the composite, uniformly intercalated rGO layers effectively operated as small current collectors for electronic transfer and conduction. In addition, the nanoporous CMOF-5 efficiently inhibited the self-restacking of the composite via incorporation of rGO. Therefore, rGO/CMOF-5-based working electrode exhibited a good performance in 6 M KOH electrolyte. In the 3E cell, it showed high capacitances of 265 F g^−1^ at 40 mV s^−1^ and 312 F g^−1^ at 0.5 A g^−1^. MOF-5/GO composite (GMOF)-derived C-GMOF was prepared by two-step pyrolysis (270 °C, 30 min in air and 800 °C, 2 h, N_2_ flow) and following 2 M HCl treatment [114]. The obtained C-GMOF had “brick-and-mortar” lamellar structure with high S_BET_ as well as a large V_total_ (1.43 cm^3^ g^−1^). The basic role of brick (2D graphene sheets) was a conductive and structural substrate, and that of mortar (carbon film with interconnected mesoporosity) was a kind of divider of bricks for charge storage sites and ion transfer channels. In the 3E mode with 6 M KOH electrolyte, it yielded 345 F g^−1^ at 2 mV s^−1^ and 201 F g^−1^ (402 mF cm^−2^) even at the fast scan rate of 2 V s^−1^. In the 2E mode with 1 M Na_2_SO_4_ electrolyte, C-GMOF//C-GMOF cell delivered a maximum energy density of 30.3 W h kg^−1^ at a power density of 137 W kg^−1^.

##### ZIF-8-Derived Composites

Composites containing reduced graphene oxide (rGO). ZIF-8-derived carbon/graphene composite was first reported for supercapacitor electrode application [115]. ZIF-8/3D-graphene (G-ZIF8) composite was synthesized via a counter diffusion synthetic strategy. After carbonization of the G-ZIF-8 at 800 °C (4 h, Ar flow), HCl washing was conducted to afford a product named C-GZ. As a result of effective union of porous ZIF-8 to the 3D-graphene (hydrothermally reduced GO), the C-GZ had enhanced S_BET_ (280.4 m^2^ g^−1^) compared to the bare 3D-graphene (186 m^2^ g^−1^). In addition, in comparison with microporous C-ZIF-8, the C-GZ exhibited micro-/meso-/macroporous hierarchical porosity. This multilength scale porosity was a very favorable factor for good capacitive performance. Therefore, binder-free and conducting agent-free working electrode from the C-GZ for SSC yielded good results in 1 M H_2_SO_4_ electrolyte. The C-GZ electrode showed a high capacitance at 0.1 A g^−1^ as well as high rate capability up to 50 A g^−1^ (86% retention). In addition, at 5 A g^−1^, it showed good cycling stability after 5000 cycle (84% capacitance retention, 90.3% coulombic efficiency). Moreover, C-GZ-2 derived from G-ZIF-2 (two-cycled growth of the ZIF-8 on the graphene) showed improved results because of the increase of both S_BET_ (352.8 m^2^ g^−1^) and D_pore_ (from 1.6 to 4 nm). Its capacitance obtained at 1 A g^−1^ was 238 F g^−1^ and capacitance improvements could be seen up to 50 A g^−1^. The electrochemical capacitive properties of carbon/carbon composites are summarized in Table 7.

The N-doped nanoporous carbon/graphene (NPC/G) network hybrids derived from ZIF-8/graphene (ZIF-8/G) composite were prepared for supercapacitor electrode materials [116]. A homogeneous encapsulation of the ZIF-8 to GO nanosheets started from the electrostatic interaction-driven adsorption of Zn^2+^ ions onto O-functionalized GO surfaces. After the homogeneous nucleation of ZIF-8, the ZIF-8 formed passive layers to inhibit aggregation of rGO during carbonization process at 800 °C (5 h, N_2_ flow). In this way, the carbonized product resulted from freeze-dried composite was transformed into the NPC/G. The acid-treated NPC/G (S_BET_ = 703 m^2^ g^−1^) contained hierarchical micro-/meso-/macroporosity. Thus, large contact area between electrode and electrolyte along with improved ionic diffusion could be seen in the NPC/G. Moreover, high content of N-dopant (11.17 wt.%) induced simultaneous enhancements of electronic conductivity and pseudocapacitance. Therefore, these factors could account for good electrochemical performance of the NPC/G in a 3E system with 1 M KOH electrolyte. It showed high capacitances with acceptable rate capabilities (136 F g^−1^ at 100 mV s^−1^ and 119 F g^−1^ at 5 A g^−1^).

The carbonization of in situ synthesized GO@ZIF-8 carbon precursor and subsequent HCl (5 wt.%) treatment were conducted to prepare graphene-based N-doped porous carbons (GNPC) [117]. The obtained GNPC, which had a long-range 2D ordering of graphitic carbon structure, contained layered morphology, high N-dopant content (10.61%), and well-defined porosity. In the 3E configuration coupled with 6 M KOH electrolyte, the GNPC-based electrode showed a capacitance of 144 F g^−1^ at 0.1 A g^−1^. This result was attributed to synergistic effects of graphene structure and N-dopant. The PVP-aided ultra-sonification method was introduced as an exfoliation method of GO. The uniform growth of ZIF-8 on the exfoliated GO surface was achieved for the preparation of ZIF-8/GO precursor [118]. In the precursor, the layered GO structure prevented the aggregation of ZIF-8 crystals. The ZIF-8/GO was converted into HPNCs/rGO-800 by a single-step carbonization at 800 °C under N_2_ flow. Although it had lower S_BET_ than HPNCs derived from bare ZIF-8 (820 m^2^ g^−1^), its higher graphitization degree (*I*_G_/*I*_D_ = 1.2 vs. 10.1), larger N-dopant content (13.53 at% vs. 10.18 at%), and mesoporosity led to better capacitive results than those of HPNCs.

**Figure 8:**
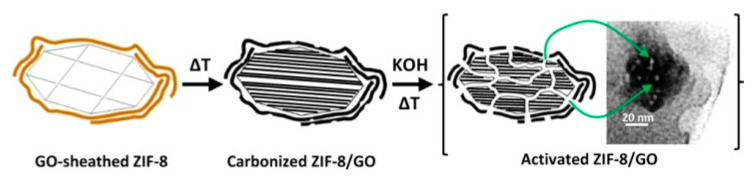
Mesopore generation process to the activated ZIF-8/GO composite via “Nanopore Lithography” approach [119].

A novel mesopore-templating approach of “Nanopore Lithography” (Figure 8) was introduced for the fabrication of carbon composite with hierarchical porosity [119]. In this approach, GO nanosheets were used as both mesopore template and lithography mask. The uniform-sized ZIF-8 obtained by using 25 mg mL^−1^ of Hmim concentration was effectively wrapped by the GO nanosheets with the help of PVP-induced interaction. After carbonization of ZIF-8/GO at 700 °C (30 min, N_2_ flow), the carbonized ZIF-8/GO along with generation of incipient porosity was obtained. This incipient porosity in the carbonized ZIF-8/GO was employed as selective activation site for the mesopore formation during KOH activation process (weight ratio of KOH/C = 1; 800 °C, 1 h, N_2_ flow). The resultant carbon composite activated at 800 °C, ZIF-8/GO (1|800), had 2D-layered network morphology with well-defined mesoporosity (~3–4 nm diameters). Moreover, it also showed high S_BET_ and V_total_ (1.219 cc g^−1^). Therefore, it gave a high capacitance of 246 F g^−1^ at 2 mV s^−1^ for the 3E cell with 1 M H_2_SO_4_ electrolyte.

The composites of N-doped carbon decorated graphene sheets (NCGs) were synthesized from in situ crystal growth of ZIF-8 on GO sheets and the subsequent carbonization of ZIF-8/GO and 3 M HCl treatment [120]. With no aggregations of the graphene sheets or the ZIF-8 derived carbon, the unique sandwich-like structure of the NCGs induced highly accessible surface area for ionic diffusion and electron transport. The optimized sample of NCGs-800 (800 °C, N_2_ flow) contained high S_BET_ (816.4 m^2^ g^−1^) and with an appropriate N-doping (18.6%). As a result, NCGs-800 electrode in a 3E cell in 6 M KOH electrolyte had capacitances of 225 F g^−1^ at 0.5 A g^−1^ and 181 F g^−1^ at 20 A g^−1^. NCGs-800//NCGs-800 cell operating in 1 M Na_2_SO_4_ electrolyte also showed good cycling stability. This symmetric cell had maximum values of capacitance (28.5 F g^−1^) and energy density (12.7 W h kg^−1^) at 0.5 A g^−1^ with a power density of 447 W kg^−1^. An easy and green synthetic process of hierarchically carbon composite (HC) derived from ZIF-8@GO hybrid was first applied to flexible solid-phase supercapacitor [121]. The precursor of ZIF-8@GO was constructed via in situ growth of nanoscale ZIF-8 on the surface of GO layer in aqueous environment. ZC@G-x (x = the weight of GO) was obtained by carbonization of the precursor at 800 °C (5 h, Ar) followed by 2 M HCl washing, then ZC@G-x was transformed into HC-x-y (y = mass ratio of KOH/ZC@G-x) through KOH activation at 700 °C (2h, Ar flow) and subsequent 2 M HCl treatment. The HC-x-y had hierarchical structure with strong binding between ZIF-8 derived micro-/mesoporous carbons and highly conductive graphene networks. Moreover, HC-40-4 showed ultra-high S_BET_ with large V_pore_ (2.12 cm^3^ g^−1^) and well-defined mesoporosity (D_pore_ = 3.03 nm) compared to the control sample of microporous ZC@G-40. It also presented appropriate heteroatom doping (O = 9.68 at%, N = 2.41 at%), which enhanced electrical conductivity (6678 S m^−1^), pseudocapacitance, and surface wettability. By using the ionic liquid electrolyte of [EMIM][BF_4_], symmetric HC-40-4//HC-40-4 cell exhibited capacitances of 206 F g^−1^ at 0.5 A g^−1^ and 122 F g^−1^ at 50 A g^−1^,. It also delivered high energy density (87.5 W h kg^−1^ at 438 W kg^−1^) and high power density (43750 W kg^−1^ at 52 W h kg^−1^). When the polymer gel electrolyte of [EMIM][BF_4_]/PVDF-HFP was used for the symmetric cell, the flexible solid-phase supercapacitor showed capacitances of 201 F g^−1^ at 0.5 A g^−1^ and 117 F g^−1^ at 50 A g^−1^, ultra-high energy density (86 W h kg^−1^ at 438 W kg^−1^) and power density (17500 W kg^−1^ at 61 W h kg^−1^).

Binder-free electrode of NC/rGO nanopaper was synthesized from NC/GO and applied to flexible SSC. The N-doped porous carbon polyhedrons (NC) were prepared by carbonization at 900 °C (2 h, Ar flow) and 2 M HNO_3_ treatment [122]. After that, the NC/GO nanopaper was produced by sonification in DMF and subsequent vacuum filtration on a nylon filter. By thermal reducing process at 500 °C under Ar, the NC/GO was transformed into the NC/rGO with a special inter-layered structure and strong interfacial interactions. The best sample of NC/rGO-2 (from 10 mg NC) had high S_BET_, large V_total_ (0.73 m^2^ g^−1^), and hierarchical porosity (micro-/mesopores) and proper heteroatom contents (N = 2.35 at%, O = 14.73 at%). Therefore, the binder-free electrode showed good electrochemical activities in a 2E cell with 6 M KOH electrolyte. A new precursor of ZIF-8@GQDs (GQDs = graphene quantum dots) was first introduced to the synthesis of C(ZIF-8)@GQDs by Yuan et al. [123]. The precursor was facilely prepared from in situ self-assembly between Zn^2+^ ions and imidazole-modified GQDs. After carbonization of the precursor at 1000 °C (3 h, N_2_ flow) and acid treatment (1 M HCl), the C(ZIF-8)@GQDs (V_pore_ = 0.807 cm^3^ g^−1^) was produced, which had narrow distribution of mesopore (~2.3 nm) and higher C- and N-dopant contents (C = 84.58%, N = 5.67%) than bare C(ZIF-8). As a result, the 3E cell with 6 M KOH electrolyte, C(ZIF-8)@GQDs-based working electrode showed high capacitance (159.6 F g^−1^ at 0.25 A g^−1^), and good rate capacity (115 F g^−1^ at 10 A g^−1^).

Composites containing CNT. CNTs/ZIF-8 precursor was first employed for the preparation of hierarchical porous CNT/porous carbon polyhedrons (hCNT/PCP) composite [124]. The precursor was prepared from electrostatic bonding between Zn^2+^ ions and –COOH functionalized CNT followed by in situ homogeneous loading of ZIF-8 crystals. After carbonization of the CNTs/ZIF-8, the resultant hCNT/PCP showed large S_BET_, V_pore_ (1.13 cm^3^ g^−1^), proper N content (9.43 wt.%), and enhanced electrical conductivity due to high graphitization degree of carbon (*I*_G_/*I*_D_ = 1.10) compared to bare PCP (*I*_G_/*I*_D_ = 0.97). Moreover, the hCNT/PCP formed a conductive network composed of CNT-embedded-PCP with hierarchical porosity. As a result, the hCNT/PCP-based SSC operating in 1 M NaCl electrolyte showed a capacitance of 104.2 F g^−1^ at 5 mV s^−1^. A new hybrid of C-ZIF-8@MWCNT was synthesized from ZIF-8@MWCNT with necklace-like structure [125]. MWCNTs was modified their surface by PVP. Then, the oriented growth of ZIF-8 on the modified surface of MWCNTs occurred. The necklace-like ZIF-8@MWCNT was carbonized at 800 °C (3 h, N_2_ flow) and treated with 3 M HCl to produce the C-ZIF-8@MWCNT. The C-ZIF-8@MWCNT (V_pore_ = 0.9867 cm^3^ g^−1^) showed synergistic effects of high conductivity (from MWCNT and partially graphitized C-ZIF-8) and hierarchical porosity (micropores from ZIF-8 and mesopores from MWCNT). Therefore, it exhibited high capacitances and good rate performance (225.5 F g^−1^ at 100 mV s^−1^ and 298 F g^−1^ at 10 A g^−1^). Pan et al. reported CNTs/NCP (NCP = N-doped carbon polyhedrons). CNTs/ZIF-8 precursor was synthesized via electrostatic adsorption of Zn^2+^ ions onto acid-treated CNTs and following coordinative in situ crystal growth of ZIF-8 [126]. The CNTs/NCP with intertwined architecture was prepared by carbonization of the precursor at 1000 °C (4 h, N_2_ flow). The obtained CNTs/NCP (V_pore_ = 1.31 cm^3^ g^−1^) with high N-dopant content (9.43 wt.%) showed hierarchical porosity: microporosity from ZIF-8, mesoporosity from small pore connection and CNT, and macroporosity from interparticle voids. Due to the 3D conducting network as well as hierarchical porosity, it showed good electrochemical results at 1 M H_2_SO_4_ electrolyte. In a 3E cell, it had a capacitance of 308 F g^−1^ at 5 mV s^−1^. Moreover, in a 2E cell, the symmetric cell assembled from the CNTs/NCP yielded high cell capacitance (135.0 F g^−1^ at 5 mV s^−1^).

The size-controlled ZIF-8 crystals were introduced as MWCNT/ZIF-8 precursor for the preparation of MWCNT/N-doped porous carbons (MWCNT/NPCs) composite [127]. Different sized ZIF-8 crystals were grown on the surface of acid-functionalized MWCNTs. The resultant MWCNT/ZIF-8-S/M/L (S = small; M = middle; L = large) precursors were directly carbonized at 800 °C (5 h, Ar flow) and then washed with acid (35% HCl) to obtain MWCNT/NPC-S/M/L products containing NCPs (high surface area, N-doping) and MWCNTs (improved conductivity, hierarchical porosity). Among the three products, the MWCNT/NPC-L derived from large-size ZIF-8 had the highest value of S_BET_ and V_total_ (0.64 cm^3^ g^−1^) because of the effective inhibition of NPC particle aggregation. Thus, MWCNT/NPC-L-based working electrode showed superior capacitive results in 1 M H_2_SO_4_ electrolyte. For the 3E cell, it yielded high capacitances (293.4 F g^−1^ at 200 mV s^−1^, 302.2 F g^−1^ at 2 A g^−1^) with excellent rate capabilities (219.5 F g^−1^ at 200 mV s^−1^, 247.8 F g^−1^ at 10 A g^−1^). For the 2E cell, it delivered a high capacitance (112.4 F g^−1^) and an energy density (12.65 W h kg^−1^) with a power density of 225.1 W kg^−1^ at 0.5 A g^−1^.

Peng et al. reported new binder-free electrodes derived from ZIF-8/CNT composite thin film [128]. First, oxidized CNTs with negative charge and zinc hydroxide nanostrands (ZHN) with positive charge were mixed to form a homogeneous suspension. After that, a free-standing thin film of ZHN/CNT was prepared via vacuum filtration of the suspension on polycarbonate (PC) substrate. The ZHN/CNT was reacted with 2-methylimidazole in EtOH/H_2_O solution to obtain ZIF-8/CNT. The ZIF-8/CNT-N (N = weight ratios of ZIF-8/CNT: 5/1, 10/1, and 15/1) was converted into CNT-threaded N-doped porous carbon film (CNCF-N) through carbonization at 900 °C (3 h, N_2_ flow) and diluted HCl treatment. The lightweight and free-standing CNCFs had good mechanical flexibility, high S_BET_, hierarchical porosity with micropores and mesopores, proper heteroatom doping, and suitable electrical conductivity. Among the three samples, the CNCF-10/1 showed maximum S_BET_. Therefore, the CNCF-10/1 electrode operating under 6 M KOH electrolytic condition with 3E configuration exhibited a high capacitance of 340 F g^−1^ at 2 A g^−1^ with moderate rate capability of 43% at 400 A g^−1^. In addition, with 2E configuration, it delivered high maximum capacitance of 191.2 F g^−1^ and an energy density of 26.6 W h kg^−1^ at 1 A g^−1^.

Wang et al. synthesized noble CNT/N-doped porous carbon composites (CNT@CZIFs) from carbonization of CNT/ZIF-8 composite at 800 °C (3 h, Ar flow) followed by acid (2 M HCl) washing [129]. Through the effective introduction of –OH functionalities by polydopamine (PDA) coating on 2D CNT, the resultant CNT@PDA had many nucleation sites on the surface. Next, heterogeneous growth of ZIF-8 on the CNT@PDA afforded CNT@ZIF-N (ZIF-8 growth time: 3 min for CNT@ZIF-1, 30 min for CNT@ZIF-2). The CNT@PDA electrostatically attracted Zn^2+^ ions to give CNT@ZIF-N. Both CNT and ZIF-8-derived carbons in CNT@CZIF-N could synergistically affect electrical energy storage. For example, highly conducting networks of the CNT promoted ionic diffusion and reduced interfacial resistance for electron transfer. In addition, the N-doped ZIF-8-derived carbons with high surface area provided fast ionic transport route as well as rich active sites for electrochemical accessibility. Moreover, the CNT@CZIF-2 showed high surface area along with large pyridinic N-dopant content (29.56%) and mesoporosity compared to the CNT@CZIF-1. Therefore, the CNT@CZIF-2-based working electrode showed superior performance under 6 M KOH electrolytic 3E cell.

A CC substrate was used for assembling electrode material of flexible SSC using CNT containing carbon composite derived from ZIF-8 [130]. After in situ catalytic carbon layer deposition on ZnO@ZIF-8 nanorod arrays (NRAs) and subsequent reduction-evaporation steps, the ZnO@ZIF-8 NRA templates were converted into N-doped carbon bubbles on hollow carbon tube arrays (CTAs@NCBs) with 3D hierarchical porosity and high flexibility. The ZnO@ZIF-8 NRA on the CC substrate was prepared by 2-MeIM-driven ion-exchange into ZnO NRA on the substrate, where 2-MeIM is 2-methylimidazole. In addition, the deposited carbon layer on the surface of NRA template was generated from in situ thermal decomposition of ethanol vapor (2 h, N_2_ flow). Next, the resultant ZnO@C@ZIF-8 was transformed into the CTAs@NCBs via reduction-evaporation stages at 800 °C (4 h, 10 vol% H_2_/air flow). The obtained CTAs@NCBs had well-interconnected hierarchical micro-/mesoporosity along with large electroactive surface area. The 3-fold increase of 2-MeIM concentration generating ZnO@ZIF-T precursor. The optimized sample, CTAs@NCBs-700, was obtained by catalytic conversion of the ZnO@ZIF-T precursor at 700 °C. CTAs@NCBs-700 yielded good capacitive results. In the 3E configuration with 1 M H_2_SO_4_, it showed high capacitances (244 F g^−1^ at 0.67 A g^−1^, 366 mF cm^−2^ at 1 mA cm^−2^) and good rate performance (139 F g^−1^ at 134 A g^−1^, 208 mF cm^−2^ at 20 mA cm^−2^). In the 2E cell with 1 M H_2_SO_4_, it delivered high areal capacitances (580 mF cm^−2^ at 1 mA cm^−2^, 241 mF cm^−2^ at 200 mA cm^−2^) and volumetric capacitances (7.6 F cm^−3^ at 0.013 A cm^−3^, 3.2 F cm^−3^ at 2.6 A cm^−3^).

Composites containing CN. A facile and economical synthesis of ZIF-8/melamine-derived nanocomposite (ZM-C-800) was accomplished via carbonization at 800 °C (3 h, N_2_) and a subsequent washing (2 M HCl) [131]. The ZIF-8/melamine composite (weight ratio of ZIF-8/melamine = 5) was prepared by a simple sonification. The pre-melted melamine layer was formed at an early stage of the carbonization, and this layer provided stable structural integrity to the composite. The ZM-C-800 contained a 3D broccoli-like carbon nitride architecture, high graphitization degree, ultra-high nitrogen content (28.3 at%), and hierarchical porosity (micro- and mesopore). In the 3E cell with 6 M KOH electrolyte, it showed high specific capacitances, suitable rate performance (253.6 F g^−1^ at 20 A g^−1^), and good cycling durability. In the same electrolyte at 0.5 A g^−1^, the symmetric ZM-C-800//ZM-C-800 cell delivered high values of capacitance (118.5 F g^−1^) and energy density (16.4 W h kg^−1^) along with a power density of 249.1 W kg^−1^. In addition, at 10 A g^−1^, it yielded a power density (4985 W kg^−1^) with good retention (80 F g^−1^, 11.4 W h kg^−1^). Graphitic carbon nitride/N-doped carbon polyhedral particles (g-CN/NCPPs) hybrid was synthesized via two-step carbonization and acid etching (2 M HCl) [132]. The carbonization of ZIF-8 at 800 °C (3 h, N_2_ flow) produced Zn-NCPPs. Then, the mixture of Zn-NCPPs, urea, and glucose was calcined in air at 550 °C (3 h). The second carbonization of the calcined sample at 800 °C (1 h, N_2_) generated g-CN/Zn-NCPPs. The acid washing of the g-CN/Zn-NCPPs gave the final g-CN/NCPPs. The optimized product of g-CN/NCPPs_0.1_ (obtained from 0.1 g of Zn-NCPP) had high S_BET_, well-defined hierarchical porosity (meso- and macropores), and high level of N-doping (20.55%). Therefore, in the 3E cell with 1.0 M H_2_SO_4_ electrolyte, it yielded ultra-high capacitance and moderate rate capacity (188.0 F g^−1^ at 20 A g^−1^). In addition, in the 2E cell with the same electrolyte, its maximum capacitance (349.7 F g^−1^) and energy density (11.89 W h kg^−1^) were obtained at 0.5 A g^−1^ with a power density of 247.40 W kg^−1^.

Other ZIF-8-derived composites. The graphitic carbon nitride polyhedron (GCNP) electrode for flexible supercapacitor with high durability and energy density was prepared by the carbonization (3 h, N_2_ flow) of CNTs wired ZIF-8 composite (CNTs/ZIF-8) and a subsequent acid washing (2 M HCl) [133]. The CNTs/ZIF-8 composite was fabricated by in situ ZIF-8 growth on acid-treated CNT, and then transformed into the GCNPs with a highly N-doped continuous hierarchical structure. Among the GCNPs, GCNP-800 (carbonized at 800 °C) showed the highest value of S_BET_ and electrical conductivity (278 S m^−1^) with O-dopant content (7.03 at%), hierarchical porosity (D_pore_ = 2.5 nm), and large N-dopant content (17.82 at%). Therefore, the GCNP-800 working electrode operating in a 3E cell with 1 M H_2_SO_4_ electrolyte yielded ultra-high maximum capacitance (426 F g^−1^ at 1 A g^−1^) and moderately good rate performance up to 10 A g^−1^. With the introduction of a new electrolyte of 70 wt.% polyoxyethylene/nitrile butadiene (PEO/NBR) interpenetrating polymer network (IPN) with 1-ethyl-3-methylimidazole tetrafluoroborate (EMIBF_4_), which had higher ionic conductivity and mechanical stability than PVA, the GCNP-800-based solid-phase SSC showed a maximum capacitance of 190 F g^−1^ and a large maximum energy density of 59.40 W h kg^−1^ at 1 A g^−1^. It also demonstrated high structural stability and flexibility (at twisted state, stretchable state, and bending state) and long-term cycled foldability.

**Figure 9:**
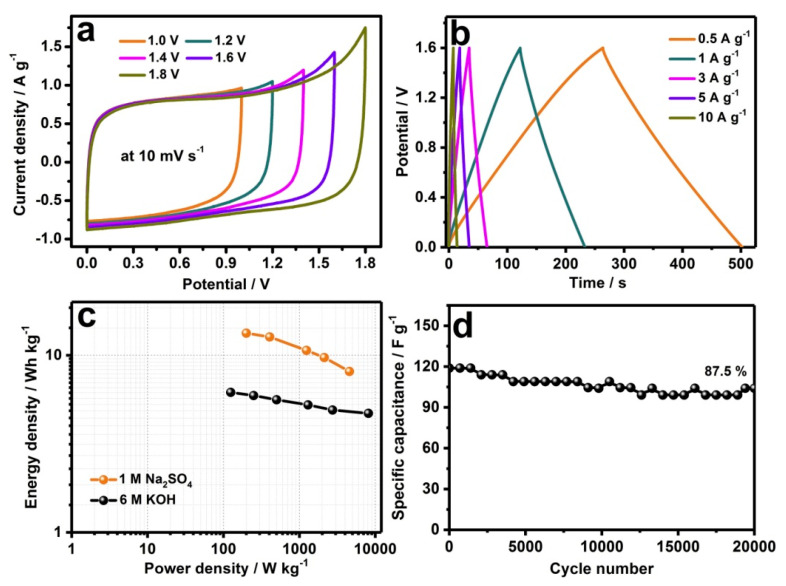
Electrocapacitive features for SSC of NHCSF-3//NHCSF-3 operating in 1 M Na_2_SO_4_ electrolyte. (**a**) CVs at 10 mV s^−1^ under different conditions of potential window; (**b**) GCD curves at different current loads; (**c**) Ragone plots; (**d**) cyclic performance at 4 A g^−1^ up to 20,000 cycles [135].

Zhang et al. reported the conversion of ZIF-8@CTAB hybrid (CTAB = cetyltrimethylammonium bromide) into N-doped porous carbon composite (PC1000@C) through carbonization at 1000 °C (4 h, N_2_ flow) and acid treatment (4 M HCl) [134]. In the ZIF-8@CTAB, the surfactant micelle of CTAB was employed as capping reagents for controlling the growth rate of ZIF-8 crystals. This had a great effect on the structural and capacitive properties of PC1000@C. Compared to PC1000 derived from ZIF-8, the PC1000@C showed high S_BET_, large V_total_ (1.68 cm^3^ g^−1^), and hierarchical micro-/mesoporosity. Therefore, the PC1000@C showed better rate performance (65%) than the PC1000 (53%) when the current load extended from 0.5 to 6 A g^−1^ under 3E mode in 6 M KOH electrolyte. However, at 0.5 A g^−1^, the PC1000 yielded slightly higher maximum capacitance than PC1000@C (225 F g^−1^). This small improvement was ascribed to the enhanced pseudocapacitive N-dopants level (10.2%) and graphitization degree (R = 2.28) of PC1000. Qiu et al. synthesized N-rich hollow carbon shell frameworks (NHCSHs) with 3D structure comprised of hollow porous shells and 2D thin porous nanoflakes derived from core/shell *PP*-SiO_2_/ZIF-8 hybrid precursor [135]. The polyelectrolyte-decorated SiO_2_ (*PP*-SiO_2_) was synthesized through the treatment of 30 wt.% poly(styrene sulfonic acid sodium salt) (PSS) to PDDA-decorated SiO_2_ (*P*-SiO_2_, PDDA = poly(diallyldimethylammonium chloride)). The PDDA-modified SiO_2_ was designated as *PP*-SiO_2_-n (n = amount of added SiO_2_ in g). After electrostatic ion adsorption of Zn^2+^ ions on the negatively charged surface of *PP*-SiO_2_-3, coordination bond-driven reaction of 2-MeIM ligand occurred and the *PP*-SiO_2_-3/ZIF-8 formed where nano-sized ZIF-8 crystals were deposited on the *PP*-SiO_2_-3 surface. The optimized sample of NHCSF-3 was prepared through the carbonization of *PP*-SiO_2_-3/ZIF-8 and subsequent acid etching (1 M HCl and 5% HF). No additional activation process was conducted. The NHCSF-3 with hierarchical porosity held high S_BET_, large V_total_ (1.85 cm^3^ g^−1^), and large heteroatom contents (N = 20.29 at%, O = 8.27 at%). In the 3E setup with 6 M KOH electrolyte, its specific capacitances decreased from 253.6 to 200.4 F g^−1^ (79.0% retention) when current densities increased from 1 to 50 A g^−1^. In the same electrolyte with 2E setup, it showed a high specific capacitance (~180 F g^−1^ at 1 A g^−1^) and a good rate performance (76% retention, 135.4 F g^−1^ at 30 A g^−1^) (Figure 9).

A new and complex carbon composite of N-GQD@cZIF-8/CNT with 3D hierarchical structure was synthesized in the same year through carbonization (800 °C, 8 h, N_2_ flow) and acid washing (2 M HCl) steps and a subsequent electrochemical deposition (2 V, 3 h) of N-doped graphene quantum dots (N-GQDs) [136]. ZIF-8 crystals were uniformly grown on –COOH-functionalized CNTs with PVP modulator, and then CNT-crosslinked ZIF-8 (ZIF-8/CNT) formed. After the carbonization-washing steps, the obtained precursor of c-ZIF-8/CNT with hierarchical networks contained a highly active surface area from c-ZIF-8 as well as good conductivity and mechanical properties from CNTs. Therefore, the c-ZIF-8/CNT could act as an effective template for attracting the N-GQDs. The colloidal N-GQD was prepared from 1,3,6-trinitropyrene and hydrazine hydrate via hydrothermal method at 200 °C (12 h) followed by filtration. In addition, the N-GQD provided efficient pseudocapacitance and surface wettability to the N-GQD@cZIF-8/CNT (S_BET_ = 520 m^2^ g^−1^, N = 16.3 at%). As the result of synergistic effects of pseudocapacitance from N-GQD, accessible surface area from c-ZIF-8, and good electrical conductivity from CNT, the N-GQD@cZIF-8/CNT-based SSC showed an excellent performance in both 1 M H_2_SO_4_ and H_2_SO_4_/PVA electrolytes. In the aqueous electrolyte, it yielded a great areal capacitance (622 mF cm^−2^ at 1 mA cm^−2^), appropriate rate performance (61.5% retention from 0.5 to 20 A g^−1^), a ultra-high energy density (18.75 W h kg^−1^ at 108.7 W kg^−1^) and a high power density (2175 W kg^−1^ at 12.62 W h kg^−1^). The flexible SSC based on the N-GQD@cZIF-8/CNT with the gel electrolyte delivered ultra-high capacitances (400 F g^−1^, 564 mF cm^−2^) and high energy densities (14 W h kg^−1^, 78.3 μW h cm^−2^) with a power density of 89.5 W kg^−1^ (0.5 mW cm^−2^) at 0.3 A g^−1^. More importantly, the flexible solid-phase device demonstrated outstanding mechanical stabilities.

#### 4.1.2. Co-MOF-Derived Composite

Table 8 shows the capacitive data for MOF-derived carbon/carbon composite-based electrode materials. The Co-MOF, Cu-MOF, and Zr-MOF are good precursors for these composites.

The Co-MOF-derived carbon/N-doped graphene aerogel composite (C/NG-A) was prepared via carbonization of Co-MOF/NG-A at 750 °C (2 h, Ar flow) and HCl treatment [137]. As a first step, the dispersed GO was hydrothermally reacted with ammonia to produce N-doped graphene hydrogel. Next, the Co-MOF (ZIF-67) polyhedrons were loaded into the hydrogel, and supercritical CO_2_ drying and subsequent thermal activation of the hydrogel were conducted to form the Co-MOF/NG-A precursor for carbonization. The C/NG-A contained high S_BET_, uniform mesoporosity (D_pore_ = 5 nm), and interconnected graphene networks. In the 3E cell using 1 M H_2_SO_4_, the C/NG-A-based working electrode yielded a high capacitance with great rate performance (305 F g^−1^ at 50 A g^−1^). Using PVA/H_2_SO_4_ electrolyte, solid-phase SSC showed outstanding recycling stabilities.

#### 4.1.3. Cu-MOF-Derived Composite

HKUST-1/CNT was introduced as a precursor for the fabrication of hierarchical porous carbon films (HPCFs) [138]. The precursor was prepared from the assembly between positively charged copper hydroxide nanostrands (CHNs) and negatively charged CNT. The separation of solids by filtration produced CHNs/CNT thin film. The immersion of CHNs/CNT thin film in ethanol-water solution of H_3_btc afforded HKUST-1/CNT thin film. The HKUST-1/CNT thin film was converted into HPCFs with meso-/macroporosity via carbonization (3 h, N_2_ flow) and acid etching (0.7 M HNO_3_). With the fine tuning of the carbonization temperature, i.e., 800 °C, and weight ratio (HKUST-1/CNT = 40/1), the optimized HPCF4 had the largest S_BET_ and V_total_ (0.63 cm^3^ g^−1^) among the HPCFs. In addition, the HPCF4 with hierarchical pore structure also exhibited a good electrical conductivity of 1320 S m^−1^. As a result, both HKUST-l-derived carbon polyhedron and CNT had synergetic effects on the electrochemical performance of flexible and binder-free electrode based on the HPCF4 for supercapacitor in 6 M KOH electrolyte. In the 3E mode, it showed high capacitances and good rate performance (253.9 F g^−1^ at 200 mV s^−1^, 120.9 F g^−1^ at 100 A g^−1^). In the 2E mode, the HPCF4//HPCF4 showed an outstanding rate performance (157.9 F g^−1^ at 1 A g^−1^ and 128.9 F g^−1^ at 10 A g^−1^) and a high energy density (9.1 W h kg^−1^ at 3500 W kg^−1^).

#### 4.1.4. Zr-MOF-Derived Composite

The carbonized UiO-66-NO_2_/reduced carboxyl graphene (CUiO-66-NO_2_/rCXYG) was prepared from the carbonization of a UiO-66-NO_2_/carboxyl graphene composite (5 h, N_2_ flow) followed by HF treatment [139]. The conductive graphene layer was effectively incorporated into bare C_2_-700 to give a high-surface precursor leading to the optimized sample C-700 carbonized at 700 °C. The graphene layer successfully inhibited the aggregation of precursor particles. As a result, the C-700 had high S_BET_, large V_tot_ (0.79 cm^3^ g^−1^), well-defined microporosity (most probable diameter = 1.061 nm; V_micro_/V_total_ = 72%), suitable mesoporosity (2.0–8.0 nm), and good surface wettability. Therefore, in the 3E cell with 6 M KOH electrolyte, it showed a high capacitance of 302 F g^−1^ at 0.15 A g^−1^**.**

#### 4.1.5. Other Kinds

An effective synthesis of light-weighted porous carbon composite electrodes for all-solid-phase SSCs operating in PVA/KOH gel electrolyte started from heterogeneous crystal nucleation of ZIFs (ZIF-8 or ZIF-67) on 3D macroporous graphene aerogels (GAs) support [140]. The carbonized samples (GA@CZIF-8 or GA@CZIF-67) were produced after crystal growth and a subsequent carbonization at 800 °C (3 h, Ar flow). Then, the carbonized samples were transformed into final products (GA@CZIF-8-E or GA@CZIF-67-E) via acid etching (2 M HCl). The obtained GA@CZIFs-E contained 3D hierarchical porosity (interconnected mesopore from carbonized zeolitic imidazolate frameworks (CZIFs) and uniform macropore from the GA) with even distribution of nitrogen element compared to bare CZIFs-E. The GA@CZIF-67-E showed co-catalyzed graphitization compared to the GA@CZIF-8-E. Thus, it showed better capacitances than the GA@CZIF-8-E.

**Figure 10:**
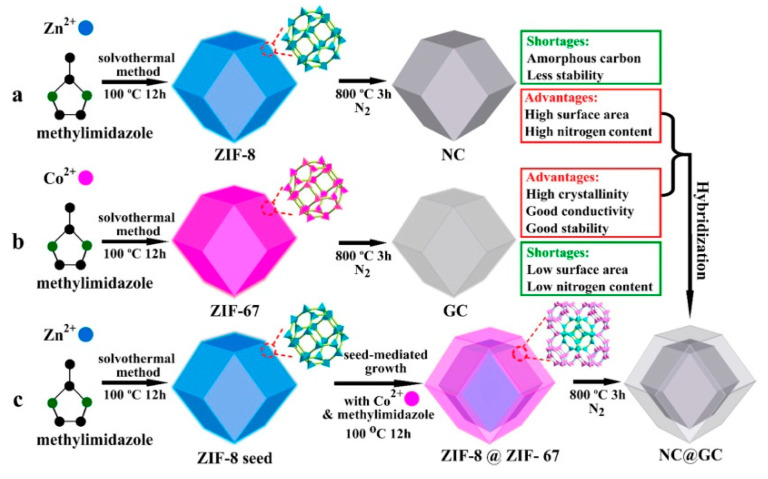
Preparation steps for (**a**) ZIF-8 crystals and NC, (**b**) ZIF-67 crystals and GC, and (**c**) core/shell ZIF-8@ZIF-67 crystals and NC@GC [141].

Yamauchi et al. reported N-doped carbon/highly graphitic carbon (NC@GC) composites by introduction of a new core/shell template of ZIF-8@ZIF-67 (Figure 10) [141]. The template was synthesized from the growth of ZIF-67 on ZIF-8 seed, and then it was converted into the NC@GC through carbonization at 800 °C (3 h, Ar flow) and acid treatment (10 wt.% HF). Co^2+^/Zn^2+^ molar ratio (x) of ZIF-8@ZIF-67(x) templates could control the core size as well as shell thickness, and eventually structural and electrochemical (3E in 1 M H_2_SO_4_) properties of the corresponding NC@GC(x) samples. To summarize, the optimized NC@GC(0.05) had high S_BET_, large V_pore_ (1.78 cm^3^ g^−1^), reasonable N-doping (10.6 wt.%), adequate shell thickness of the GC, and well-developed and hierarchically interconnected micro-/mesoporosity. Therefore, it showed satisfactory maximum capacitance and moderate rate performance.

The same group reported bimetallic-ZIF-derived nanoporous carbon (NPC) with CNT on the surface [142]. The bimetallic-template, Co/Zn-ZIF, was synthesized in a large scale without PVP modulator (Co/Zn molar ratio = 2/1). The hybrid template was transformed into Co/Zn NPC via carbonization (800 °C, 5 h, N_2_ flow) and acid washing (10 wt.% HF). With the introduction of synergistic advantages of both ZIF-8 and ZIF-67, the resultant Co/Zn NPC contained microporosity, mesoporosity, graphitic CNTs, and N-dopant (3.5 at%). As a result, in the 3E cell with 0.5 M H_2_SO_4_, it yielded capacitances of 286 F g^−1^ at 2.5 A g^−1^ and 103.7 F g^−1^ at 10 A g^−1^. Good cycling stabilities of the SSC up to 10,000 cycles could be displayed in both 0.5 M H_2_SO_4_ and 1.0 M TBAPF_6_/AN electrolytes.

### 4.2. Carbon/Metal Oxide Composite

The carbon/metal oxide composites are also good supercapacitors due to the favorable role of metal oxides for better performance. Table 9 shows the electrochemical capacitive properties of the known carbon/metal oxide composite-based electrode materials.

#### 4.2.1. Composite Containing CeO_2_

CeO_2_ has natural abundance, green features, and dynamic redox couple. Thus, CeO_2_-containing carbon composites can show good capacitive behaviors. In this regard, new rod-shaped CeO_2_@C nanocomposites were prepared from the Ce-btc MOFs synthesized under different synthetic conditions (ST = solvothermal, RT = room temperature) [143]. The MOF templates, i.e., Ce-btc (ST) or Ce-btc (RT), were transformed into the CeO_2_@C via a two-step pyrolysis (600 °C, 3 h, Ar flow and 350 °C, 2 h in air). In the CeO_2_@C, CeO_2_ was rod-shaped matrix and pseudocapacitive redox center. Moreover, partially graphitic carbons (C) provided electronic conductivity, surface wettability, and porosity. In addition, compared to CeO_2_@C(ST) (S_BET_ = 53 m^2^ g^−1^, C = 9.5 wt.%), CeO_2_@C(RT) held good crystallinity, high S_BET_ (384 m^2^ g^−1^) and carbon contents (13.4 wt.%), and developed hierarchical mesoporosity (3.4 nm for most probable pore size, 6.2 nm for average pore size, and 6.5 nm for interparticle pore size). As a result, the CeO_2_@C(RT) electrode demonstrated better performance in 2 M KOH + 0.1 M K_4_Fe(CN)_6_ electrolyte through two main electrochemical reactions in a 3E mode: non-faradaic surface adsorption-desorption, (CeO_2_)_surface_ + K^+^ + e^−^ ↔ (CeO_2_^-^∙K^+^)_surface_; faradaic intercalation-deintercalation, Ce^IV^O_2_ + K^+^ + e^−^ ↔ Ce^III^O∙OK. Additional faradaic reaction of CeO_2_ also occurred because of K_4_Fe (CN)_6_ electrolyte: K_4_Fe(CN)_6_ ↔ K_3_Fe(CN)_6_ + e^-^. The CeO_2_@C(RT) electrode showed an ultra-high capacitance of 1102 F g^−1^ at 2 A g^−1^ and moderate rate capacity (418 F g^−1^ at 20 A g^−1^),

#### 4.2.2. Composite Containing Co_3_O_4_

Co_3_O_4_ has many advantages because it is cheap, eco-friendly, and highly active in catalysis. Nevertheless, its pseudocapacitive kinetics was limited by low electric conductivity and slow ionic diffusion rate. To overcome these restrictions, Co_3_O_4_-embedded porous carbon composite (Co_3_O_4_/C) was generated from Co-MOF precursor on a flexible and conductive Ni foam substrate [144]. The precursor, Co-MOF nanowire arrays (NAs), was synthesized by solvothermal reaction of Co(NO_3_)_2_∙6H_2_O and 2,5-dihydroxybenzenedicarboxylic acid (2,5-dhbdc) on the substrate at 120 °C. After two-step thermolysis of the Co-MOF NAs (400 °C, 1 h, Ar flow and 250 °C, 2 h in air), the porous Co_3_O_4_/C NAs with synergistic architecture and hierarchical porosity were obtained. Under 3E mode in 3 M KOH electrolyte, the Co_3_O_4_/C hybrid showed a ultra-high capacitance of 1.32 F cm^−2^ (776.5 F g^−1^) at 1 mA cm^−2^ and outstanding rate performance up to 20 mA cm^−2^. Moreover, under 2E mode in PVA/KOH electrolyte, the Co_3_O_4_/C NAs-based flexible SSC showed maximum values of capacitances (203 mF cm^−2^, 61.5 F g^−1^) and energy densities (0.14 mW h cm^−3^, 8.54 W h kg^−1^) at 1 mA cm^−2^, suitable rate performance (58.9% retention) at 10 mA cm^−2^, and good long-term stabilities of mechanical bending (90.1% retention after 1000 bending cycles at 90°). These excellent results could be explained by the improved electrical conductivity and porosity from the Ni foam and MOF-derived carbon, as well as binder-free direct contact between the Co_3_O_4_/C NAs and Ni foam.

Another 3D composite of Co_3_O_4_/nanoporous carbon (Co_3_O_4_/NC) was fabricated from the new core/shell type of precursor, Co (CO_3_)_0.5_(OH)∙0.11H_2_O@ZIF-67 [145]. The Co(CO_3_)_0.5_(OH)∙0.11H_2_O was hydrothermally synthesized from the mixture of Co(NO_3_)_2_∙6H_2_O, urea, and NH_4_F at 120 °C, then it acted as a nucleation site for chemical vapor deposition (CVD)-mediated growth of ZIF-67 film to prevent self-aggregation. For the crystallinity and uniformity of the film, 2-MeIM vapor was introduced into the nucleation site via the CVD method. The precursor was generated by a subsequent heating (x °C, y h) in a sealed high-pressure reactor. The resultant (CO_3_)_0.5_(OH)∙0.11H_2_O@ZIF-67-x-y was converted into Co_3_O_4_/NC-x-y through two-step heat treatment (400 °C, 1 h, N_2_ flow and 250 °C, 2 h in air). In the Co_3_O_4_/NC-x-y, Co_3_O_4_ nanowire (NW) core was adequate for faradaic reaction site with excellent theoretical capacitance (3560 F g^−1^) while graphitic NC shell contained porous and conductive structure. The Co_3_O_4_/NC-90-15 showed high S_BET_ (74.0 m^2^ g^−1^) and good preservation of 3D NW morphology in combination with effective contact for electric conduction compared to the Co_3_O_4_/NC-90-24. In a 3E cell with 6 M KOH, it yielded high areal capacitance of 1.22 F cm^−2^ at 0.5 mA cm^−2^.

The ZIF-67 as a sole template was also introduced to the cost-effective synthesis of Co-containing nanoporous carbon (NC) polyhedrons with hollow Co_3_O_4_ shells [146]. The ZIF-67 was prepared under rapid and scalable synthetic conditions (molar ratio of Co (NO_3_)_2_:2-MeIM:triethylamine = 1:10:5). After the carbonization of ZIF-67 at 600 °C or 900 °C (6 h, N_2_ flow), as-prepared carbons named NC600 and NC900 were produced. HCl treatment of the as-prepared samples was conducted to generate acid-treated samples named NC600-AT and NC900-AT. The samples synthesized at lower temperature had higher S_BET_ (309.7 m^2^ g^−1^ for NC600, 763 m^2^ g^−1^ for NC600-AT), whereas samples synthesized at higher temperature showed larger D_pore_ (5.7 nm for NC900, 5.9 nm for NC900-AT). Under 0.1 M KOH electrolyte in a 3E cell, maximum specific capacitances at 20 mV s^−1^ were 57 (NC 600), 98 (NC600-AT), 88 (NC900), and 67 (NC900-AT) F g^−1^. Under this condition, the capacitance was mainly influenced by microporosity. However, at higher rate condition, meso-/macroporosity mostly affected on the capacitance.

#### 4.2.3. Composite Containing Copper Oxides

A simple synthetic approach was applied to the preparation of Cu-Cu_2_O-CuO/C composites. After a single-step carbonization of HKUST-1 as a single self-sacrificing template at 700 °C or 800 °C (4 h, Ar flow), Cu-Cu_2_O-CuO/C 700 (Cu1) and Cu-Cu_2_O-CuO/C 800 (Cu2) were produced [147]. The obtained composites containing suitable micropores and mesopores were composed of highly interconnected graphitic carbon fibers with O-dopant and redox-active Cu species. The electrochemical performance of the obtained electrodes were investigated by using a 3E cell in 6 M KOH electrolyte. In CV, both electrodes yielded remarkable maximum gravimetric capacitances at 2 mV s^−1^ (782 F g^−1^ for Cu1, 773 F g^−1^ for Cu2) and moderate rate retentions at 75 mV s^−1^ (41% for Cu1, 43% for Cu2). In addition, in the GCD, they had improved rate retentions from 2 mA cm^−2^ to 25 mA cm^−2^ (65% for Cu1, 67% for Cu2). Moreover, high values of areal capacitances were displayed in both 2 mV s^−1^ (351 μF cm^−2^ for Cu1, 346 μF cm^−2^ for Cu2) and 2 mA cm^−2^ (363 μF cm^−2^ for Cu1, 332 μF cm^−2^ for Cu2).

#### 4.2.4. Composite Containing Fe_3_O_4_

**Figure 11:**
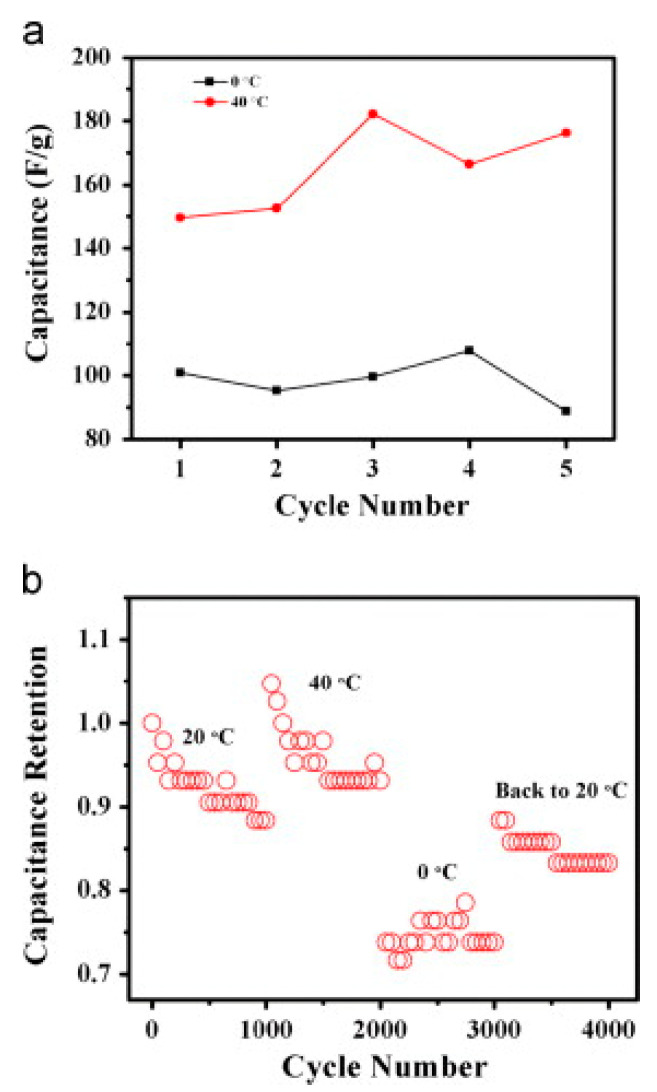
Temperature-dependent performances for composite electrode of Fe_3_O_4_/carbon using 1 M KOH electrolyte. (**a**) Specific capacitance variations at 1 A g^−1^ under repeated cycles of heating and cooling. (**b**) Cyclic stability at 2 A g^−1^ under various temperatures [148].

The MOF-derived carbon supercapacitor electrode containing Fe_3_O_4_ was synthesized, and its temperature-dependent capacitances were studied [148]. The Fe-MIL-88B-NH_2_ self-sacrificing template synthesized from the reaction mixture of F-127, FeCl_3_, acetic acid, and NH_2_-bdc was easily and economically transformed into Fe_3_O_4_/carbon composite with agglomerated rod shape from a just single-step carbonization at 500 °C (1 h, N_2_ flow). The Fe_3_O_4_/carbon with mesoporosity (D_pore_ = 3.0 nm) had both conducting carbon thin layers and pseudocapacitive Fe_3_O_4_. The GCD curves measured at 20 °C showed capacitances of 139 F g^−1^ at 0.5 A g^−1^ and 74 F g^−1^ at 5 A g^−1^ in 1 M KOH electrolyte. When the operating temperature increased from 0 to 60 °C, its specific capacitance at 1 A g^−1^ showed a dramatic increase from 86 to 162 F g^−1^, whereas its internal resistance estimated from the EIS spectra decreased from 0.8 to 0.5 Ω. It exhibited good cycling stability at varying temperature regions (83.3% retention after 4000 cycles) as shown in Figure 11.

Another Fe_3_O_4_/carbon composite was synthesized from Fe-bdc MOF [149]. After one-step carbonization (1 h, Ar flow) of the Fe-bdc MOF prepared by using F-127, FeCl_3_∙6H_2_O, acetic acid, and bdc, MOFC-T (T = 300, 400, 500, 600 and 700 °C) carbons were produced. Among them, the optimized sample of MOFC-600 had mesoporosity (D_pore_ = 3.6 nm), and its thin carbon shell with hierarchical porosity effectively covered the Fe_3_O_4_ cores. Therefore, it showed an ultra-high capacitance of 972 F g^−1^ at 1 A g^−1^ in 6 M KOH.

#### 4.2.5. Composite Containing Mn_3_O_4_

Although Mn_3_O_4_ was a cheap and green electrode material with high capacitive property, its electrochemical capacitor performance was restricted by the low electrical conductivity (10^−5^–10^−6^ S cm^−1^) and slow rates of electrolytic penetration/diffusion processes [150]. Therefore, N-rich Mn-coordination polymer particles (CPPs)-derived carbon/Mn_3_O_4_ composites were prepared to overcome these restrictions. The CPP-1 was HATC-based Mn-CPP (HATC = 3-amino-1,2,4-triazole-5-carboxylic acid), while the CPP-2 was H_2_pdc-based Mn-CPP (H_2_pdc = pyridine-2,6-dicarboxylic acid). After single-step carbonization at 450 °C (30 min, N_2_ flow), the N-doped porous carbon/Mn_3_O_4_ (NC/Mn_3_O_4_) composites were generated. The NC/Mn_3_O_4_-1 prepared from the CPP-1, and the NC/Mn_3_O_4_-2 from the CPP-2. Compared to the NC/Mn_3_O_4_-2, the NC/Mn_3_O_4_-1 showed better capacitive behaviors in 1.0 M Na_2_SO_4_ electrolytic 3E cell mainly due to its ultra-high nitrogen contents (29.87 wt.%). Moreover, its conductive NC part could improve pseudocapacitance of Mn_3_O_4_, facilitate intercalation/deintercalation of the electrolyte, and enhance electrochemical stability at long-term cycling process.

#### 4.2.6. Composite Containing MoO_2_

MoO_2_ has been known for its properties of multiple oxidation state-driven charge transfer but low capacitance. As an alternative to bare MoO_2_, MoO_2_@Cu@C composite was prepared from one-step co-carbonization of POM@MOF (POM = polyoxometalates) composite precursor at 600 °C (2 h, N_2_ flow) [151]. The precursor, [Cu_2_(btc)_4/3_(H_2_O)_2_]_6_[POM]∙(C_4_H_12_N)_2_∙xH_2_O, was hydrothermally synthesized from copper nitrate, phosphomolybdic acid, H_3_btc, and (CH_3_)_4_NOH. The negatively charged Mo-based POM was well-dispersed guests of the Cu-btc-MOF in the precursor. Therefore, the resultant MoO_2_@Cu@C (D_pore_ = 6.0 nm) had numerous micropores and mesopores. Furthermore, it contained excellent dispersity of pseudocapacitive MoO_2_, conductive Cu, and interconnected amorphous carbon sheets. Using 2 M KOH electrolyte in a 3E cell, the MoO_2_@Cu@C yielded high charge capacities (28.27 mA h g^−1^ at 5 mV s^−1^, 28.56 mA h g^−1^ at 0.5 A g^−1^). Moreover, it delivered maximum values of capacity (7.49 mA h g^−1^) and energy density (2.58 W h kg^−1^) with a minimum power density of 86.8 W kg^−1^ at 0.25 A g^−1^, and a maximum power density (790.38 W kg^−1^) with minimum values of capacity (3.33 mA h g^−1^) and energy density (0.71 W h kg^−1^) at 2 A g^−1^.

#### 4.2.7. Composite Containing RuO_2_

A pseudocapacitive RuO_2_ has several favorable factors for good electrochemical properties such as large capacitance, good electronic conductivity, and reversible charge/discharge behavior. However, it is rather expensive due to low natural abundance. Therefore, a method to take advantage of these favorable factors of RuO_2_ is necessary. In this sense, RuO_2_/ZIF-8-derived porous carbons (PCs) composites were synthesized via carbonization-acid washing process followed by a two-step thermal treatment [152]. After carbonization (500 °C, 30 min and 800 °C, 2 h in N_2_) and subsequent 1 M HCl etching, ZIF-8 precursor could be converted into the PCs with high S_BET_ and polyhedral particle shape. Next, the PCs could be transformed into the RuO_2_/PCs composites under reflux conditions (120 °C, 6 h) and subsequent thermolysis at 150 °C in air (6 h). Among the resultant RuO_2_/PCs-n, RuO_2_/PCs-3 (RuO_2_ to PCs mass ratio = 0.707 generated from addition of 10 mL RuCl_3_ solution) yielded excellent capacitive features in 1 M H_2_SO_4_ electrolyte. In a 3E cell, it exhibited a capacitance of 539.6 F g^−1^ at 1 A g^−1^ with outstanding rate performance (81.5% at 200 A g^−1^). In brief, the polyhedral shape-driven prevention of the self-stacking of PCs, graphitic N-dopant induced improvement of electrical conductivity, and heteroatom functionalities (N = 7.33% and O = 10.55%) led to synergistic effects on the excellent capacitive results showing the enhancement of both electrochemically accessible surface area of the RuO_2_/PCs-3 (S_BET_ = 198 m^2^ g^−1^ and the coexistence of micro-/meso-/macropores) and pseudocapacitance (RuO_x_(OH)_y_ + δH^+^ + δe^-^ ↔ RuO_x-δ_(OH)_y+δ_ (0 ≤ δ ≤ 2)).

#### 4.2.8. Composite Containing ZnO

Hollow ZnO/C composite was prepared from hollow Zn-containing carbonaceous (HZC) material [153]. At first, ZIF-8/glucose composite was transformed into the HZC through hydrothermal reaction (180 °C, y h). During the hydrothermal conversion, the ZIF-8/glucose was decomposed into hydrolysis products to generate acids and ZIF-8 was decomposed by these acids. The degraded ZIF-8 was reacted with the glucose-derived polymers to form the HZC. Outward diffusion of the products from the degraded ZIF-8 and inward diffusion of molecules generated by the glucose produced the hollow structure of HZC. After optimization of reaction condition for the hydrothermal transformation (glucose solution concentration = 2.5 M, reaction time = 2 h), the obtained HZC-2.5M-2h was converted into the ZnO/C via one-step carbonization (500 °C, 2 h, N_2_ flow). The resultant ZnO/C showed high values of capacitance, 394 F g^−^^1^ at 1 A g^−^^1^ and 225 F g^−^^1^ at 60 A g^−^^1^, in a neutral 1 M Na_2_SO_4_ electrolyte.

#### 4.2.9. Other Complex Composites

To achieve improved morphology control of Co_3_O_4_-based supercapacitor electrode, Fe(NO_3_)_3_-loaded Co_2_(bdc)_2_(dabco) MOF, [Fe(NO_3_)_3_@Co_2_(bdc)_2_(dabco)], was employed as a precursor for the synthesis of (Co_0.94_Fe_0.06_)_3_O_4_ NPs-incorporated hollow carbon nanowire [(Co_0.94_Fe_0.06_)_3_O_4_@CON] [154]. The layer-pillar style precursor was solvothermally synthesized. After carbonization of the precursor, the (Co_0.94_Fe_0.06_)_3_O_4_@CON with porous NW architecture was produced. As a working electrode of a 3E mode in 1 M KOH electrolyte, the product composite showed a high capacitance (162 F g^−^^1^ at 1 A g^−^^1^) and poor rate capacity (11.2 F g^−^^1^ at 8 A g^−^^1^).

Despite several advantages of using ZnMn_2_O_4_ electrode material such as high capacitance and abundant redox-active sites, there are still some limitations of dispersion, surface area, and conductivity. To overcome these adverse issues, heterometallic Zn-/Mn-MOF-derived ZnMn_2_O_4_/carbon nanorods (ZMCN) was synthesized by carbonization of the MOF self-sacrificing template at 500 °C (1 h, N_2_ flow) followed by calcination in air at 300 °C (1 h) [155]. The heterometallic 1D self-sacrificing template was prepared in the mixed solvent (EtOH/H_2_O) at room temperature using Zn^2+^, Mn^2+^, and H_3_btc. After the two-step pyrolysis, the resultant ZMCN contained 1D pore structure (S_BET_ = 143.4 m^2^ g^−1^) and uniform dispersion of ZnMn_2_O_4_ in the structural support of conductive interconnected porous carbon. In a 3E cell with 1 M Na_2_SO_4_ electrolyte, the ZMCN had very high capacitances of 589 F g^−^^1^ at 1 A g^−^^1^ and 278 F g^−^^1^ at 20 A g^−^^1^.

From the microporous Zn-MOF (BMM-9, [Zn_2_(tpo)_4/3_(dabco)]∙DMA∙H_2_O where H_3_tpo is tris(4-carboxyphenyl)phosphine oxide) containing 3D 1.7 nm cage with high stability, 3D micro-/mesoporous carbon composite of BMM-9-900 was generated by a single-step carbonization at 900 °C (5 h, Ar flow) [156]. In the BMM-9-900, there were coexisting Zn, N, P, and Zn_2_P_2_O_7_. The Zn-MOF precursor was prepared via thermal reaction of the mixture containing zinc nitrate, dabco, HBF_4_, and H_3_tpo. The BMM-9-900 had hierarchical micro-/mesoporosity, conducting carbon support, various doping elements (Zn, P, N), and pseudocapacitive active site (Zn_2_P_2_O_7_). In a 3E cell with 3 M KOH electrolyte the following redox reaction could take place: (Zn_2_P_2_O_7_) _surface_ + K^+^ + e^−^ ↔ (Zn_2_P_2_O_7_^−^∙K^+^)_surface_. The cell showed capacitances of 263.2 F g^−1^ at 5 A g^−1^ and 132.0 F g^−1^ at 10 A g^−1^.

#### 4.2.10. Other Mixed Composites

The CoMn-MOF-74 was first used as a precursor of mixed CoO and MnO incorporated carbon framework (M/MO@C) [157]. After one-step carbonization of the precursor at 700 °C (2 h, N_2_ flow), the obtained M/MO@C-700 showed 1D-nanorod structure, uniform dispersity of NPs (Co, CoO, MnO), high graphitization (*I*_D_/*I*_G_ = 0.927), developed bimodal mesoporosity (4 nm and 6 nm), and favorable wettability from –OH functionalities. In a 3E with 6 M KOH electrolyte, it showed the following diverse redox couples: CoO + OH^−^ ↔ CoOOH + e^−^, Mn_3_O_4_∙2H_2_O + OH^−^ ↔ MnOOH +Mn(OH)_3_ + e^−^, 4MnOOH +2Mn(OH)_3_ + OH^−^ ↔ (6MnO_2_)∙5H_2_O + 3 H^+^ + 6e^−^. The specific capacitances were 894 F g^−1^ at 0.5 A g^−1^ and 272 F g^−1^ at 4 A g^−1^.

Meanwhile, NiCo_2_O_4_ also had many favorable properties such as low toxicity, cheap cost, plentiful resources, and great theoretical capacitance of 1370 F g^−1^. Nevertheless, the use of NiCo_2_O_4_ electrode for electrochemical energy storage was encountered with many practical restrictions including low electrical conductivity and poor rate of electrolyte diffusion. In addition, ZnO could be used as a profitable mechanical conducting support while it showed inferior chemical stability under acidic or basic conditions. Therefore, a new binder-free electrode of ZnO@C@NiCo_2_O_4_ nanorod sheet arrays (NRSAs) on flexible CC support was prepared by thermal decomposition, coordination-driven growth, carbonization, and electrodeposition [158]. As a first step, ZnO nanorod arrays (NRs) were thermally synthesized on the CC substrate using zinc nitrate hexahydrate, hexamethylenetetramine (HMTA), and ammonia. Next, ZnO@ZIF-8 NRs with uniform core/shell structure was obtained from ion-exchange-driven ZIF-8 crystal growth on the ZnO NRs. After that, the ZnO@ZIF-8 NRs were transformed into ZnO@C NRs through a single-step carbonization at 650 °C (2 h, N_2_ flow). Finally, cathodic co-electrodeposition of Co (NO_3_)_2_ and Ni(NO_3_)_2_ (20 cycles) on the ZnO@C NRs generated the ZnO@C@NiCo_2_O_4_. The resultant NRSAs with hierarchical structure was composed of ZnO NR core for the improvements of conductivity and electron transfer, protecting carbon layer for preservation of electrolytic erosion of the ZnO NR, and ultrathin NiCo_2_O_4_ nanosheet shell as a center of faradaic redox reactions: NiCo_2_O_4_ + OH^−^ + H_2_O ↔ NiOOH + 2CoOOH +e^−^, CoOOH + OH^−^ ↔ CoO_2_ + H_2_O + e^−^. As a result, the ZnO@C@NiCo_2_O_4_ possessed a large surface area for electrochemical accessibility in 2 M KOH electrolytic 3E cell. It yielded ultra-high specific capacitance of 2650 F g^−1^ at 5 A g^−1^.

### 4.3. Carbon/Metal Sulfide Composites

Not only the carbon/metal oxide composites but also carbon/metal sulfide and other different types of composites can behave as good electrode materials for supercapacitors as given in Table 10.

#### 4.3.1. Composites Containing Cobalt Sulfides

A 2D porphyrin-based paddlewheel framework-3 (PPF-3) nanosheets (thickness = 42.7 ± 8.4 nm) was used as a starting precursor for ultrathin (thickness = 24.5 ± 6.4 nm) 2D CoS_1.097_/N-doped carbon (CoSNC) nanocomposite [159]. With the assistance of PVP surfactant, the PPF-3 nanosheets composed of the dinuclear Co_2_(COO)_4_ paddlewheel secondary building unit (SBU), tetratopic 5,10,15,20-tetrakis(4-carboxylphenyl)porphyrin (tcpp) bridging ligand, and bpy pillar ligand were synthesized in mixed solvent system (volume ratio of DMF/EtOH = 3:1). After that, the precursor was carbonized at 900 °C (3 h) and further treated with Ar/O_2_ plasma (15 min), and then transformed into the 2D CoSNC via concurrent sulfidation-carbonization process in the presence of S powder at 650 °C (5 h, Ar flow). Despite low contents (21.0 wt.%) of CoS_1.097_ NPs, their strong adsorption on the N-doped (4.57 wt.%) carbon coupled with ultrathin 2D structure of the CoSNC could account for good capacitive output of CoSNC-based working electrode in 2.0 M KOH. The working electrode yielded high capacitance and moderate rate performance (56.8% retention at 30 A g^−^^1^).

Co-MOF-derived CoS_2_@CNTs were synthesized to overcome the weakness of cobalt sulfide which could have potentially high specific capacitances. The cobalt sulfide electrode material often suffers from poor electric conductivity and low surface area [160]. The bib-based Co-MOFs (bib = 1,4-bis(imidazol-1-yl) benzene) were solvothermally prepared with different conformational bdc linkers (L = *o*-bdc for 1, *m*-bdc for 2, and *p*-bdc for 3). The different conformations of these linkers could greatly influence the lattice dimensions of the Co-MOFs and their corresponding carbon composites’ pore dimensions. Carbonization of the Co-MOFs (900 °C, 3 h, N_2_ flow) generated Co@CNTs ((*o*)-Co@CNT, (*m*)-Co@CNT, (*p*)-Co@CNT), and the Co@CNTs were further converted into CoS_2_@CNTs ((*o*)-CoS_2_@CNT, (*m*)-CoS_2_@CNT, (*p*)-CoS_2_@CNT) via gas-sulfurization process with S powder at 400 °C (3 h, N_2_ flow). Among the Co-MOFs, Co-MOF 3 had the greatest Co-L-Co separation (6.3 Å for 1 < 8.9 Å for 2 < 11.2 Å for 3) and the largest lattice dimension, so the 3-derived carbon composite ((*p*)-Co@CNT and (*p*)-CoS_2_@CNT) showed larger pore sizes with hierarchical meso-/macroporositiy. As a result, the (*p*)-CoS_2_@CNT showed many favorable properties for capacitive performance, such as hierarchical architecture, high graphitization (*I*_D_/*I*_G_ = 0.83), suitable pore size distribution. It showed ultra-high capacitances (839 F g^−1^ at 5 mV s^−1^ and 825 F g^−1^ at 0.5 A g^−1^) and good rate capacities (447 F g^−1^ at 100 mV s^−1^ and 268 F g^−1^ at 10 A g^−1^) in 2 M KOH.

Zhang et al. revealed that the solvents with different viscosities, i.e., H_2_O and ethylene glycol, used for the synthesis of Co-MOFs ([Co(tdc)(bpy)]_n_, tdc = thiophene-2,5-dicarboxylate, bpy = 4,4′-bipyridine) played a crucial role on several key features of the Co-MOFs, such as porosity, S/N ratio, size, and morphology, and the corresponding Co_9_S_8_@S-/N-co-doped carbons (Co_9_S_8_@SNCs) [161]. The Co-MOFs were synthesized in a mixed ligand system. Compared to 3D [Co(tdc)(bpy)]_n_-bulk with irregular morphology prepared from H_2_O, 3D [Co(tdc)(bpy)]_n_-cuboid prepared from ethylene glycol showed uniform morphology because the role of more viscous ethylene glycol was a crystal growth stabilizer to inhibit the aggregation of seed crystals. After carbonization at 800 °C (2 h, N_2_ flow), Co_9_S_8_@SNCC derived from the [Co(tdc)(bpy)]_n_-cuboid containing better properties (*I*_G_/*I*_D_ = 1.03, V_pore_ = 0.828 cm^3^ g^−1^) than Co_9_S_8_@SNCB derived from the [Co(tdc)(bpy)]_n_-bulk. Therefore, in a 3E with 6 M KOH electrolyte, it yielded the capacitance as high as 429 F g^−1^ at 1 A g^−1^. The value measured at 50 A g^−1^ was 336 F g^−1^.

#### 4.3.2. Composites Containing Copper Sulfides

Copper sulfides had many favorable properties as electrode materials such as high theoretical capacitance, cheap cost, green, and easy synthesis. With the introduction of a simple and concurrent carbonization-sulfidation process, HKUST-1 octahedrons, or Cu_3_(btc)_2_, were converted into Cu_1.96_S-C composites with the preserved octahedral morphology [162]. In this process, ultrasmall Cu_1.96_S NPs with a dimension around 10 nm were homogeneously incorporated into the carbon octahedrons. Through the selection of optimal reaction temperature of 650 °C, the resultant Cu_1.96_S/C-650 (V_total_ = 0.118 cc g^−1^) showed high graphitization degree (*I*_D_/*I*_G_ = 0.98) and micro-/mesoporous structures. It showed a high capacitance of 200 F g^−1^ at 0.5 A g^−1^. Another type of copper sulfide (Cu_7_S_4_) was embedded into carbon matrix via two-step treatment of the HKUST-1 [163]. In the first step, the HKUST-1 was directly carbonized at 650 °C (2 h, N_2_ flow). In the following step, sulfidation of the carbonized sample was performed with thioacetamide (TAA) under hydrothermal condition at 120 °C (6 h). The obtained Cu_7_S_4_/C nanocomposite with cobblestone-like polyhedral morphology had interconnected structure between amorphous carbon and Cu_7_S_4_ NPs. Therefore, in a 3E mode with 1 M H_2_SO_4_ electrolyte, the Cu_7_S_4_/C composite-based working electrode showed high capacitances and moderately good rate performance (80.7 F g^−1^ at 100 mV s^−1^ and 146.1 F g^−1^ at 5 A g^−1^).

#### 4.3.3. Composite Containing Indium Sulfides

Huh et al. reported new porous carbon materials (PCMs) with uniform incorporation of indium sulfide NPs into carbon matrix [164]. After one-step carbonization of In-MOF ([Et_2_NH_2_] [In(tdc)_2_]·DEF) (2 h, N_2_ flow), the resultant PCMs showed concurrent incorporation of heteroatom dopants (S and N) and In-derived NPs (major species = In_6_S_7_; minor species = In and In_2_S_3_). Among the PCMs, PCM-900 carbonized at 900 °C showed the maximum values of surface area (S_micro_ = 405 m^2^ g^−1^), pore volume (V_total_ = 0.40 cm^3^ g^−1^, V_micro_ = 0.21 cm^3^ g^−1^), and Horváth–Kawazoe (HK) pore size (0.61 nm). Thus, in a 3E system with 1 M Na_2_SO_4_ electrolyte, the PCM-900 electrode yielded maximum values of capacitance and energy density (99.0 F g^−1^ and 13.7 W h kg^−1^ at 0.05 A g^−1^) and good rate capability (78.0 F g^−1^ at 10 A g^−1^).

#### 4.3.4. Composite Containing MoS_2_

From the successive three-step process, i.e., exfoliation, ZIF-8 growth, and carbonization, MoS_2_@MPC with a coating of microporous carbons on the 2D MoS_2_ nanosheets was synthesized [165]. As a first step, bulk MoS_2_ layered material was mechanically exfoliated under sonication and then multi-laminated MoS_2_ with 2D sheet architecture was prepared. Then, MoS_2_@ZIF-8 composite was produced via uniform growth of ZIF-8 crystals on the exfoliated MoS_2_ nanosheets at room temperature (co-solvent volume ratio of H_2_O:EtOH = 1:4, mass ratio of MoS_2_:Zn(Ac)_2_·2H_2_O:2-MeIM = 0.9:10:30). At the final step, the MoS_2_@ZIF-8 was converted into the MoS_2_@MPC through a single-step carbonization at 900 °C (2 h, Ar flow). The obtained MoS_2_@MPC nanocomposite consisted of pseudocapacitive 2D MoS_2_ nanosheets and ZIF-8-derived amorphous MPC. The MPC shell was good for protection and conduction. Thus, in a 3E cell with 1 M H_2_SO_4_ electrolyte, it yielded capacitances of 189 F g^−1^ at 1 A g^−1^.

#### 4.3.5. Other Kinds

Five M_x_S_y_@C composites were synthesized from one-pot treatment (carbonization-sulfurization) of five MOFs with the same formula [M(pa)(bib)]_∞_ (M = Co^II^, Zn^II^, Cd^II^, Ni^II^, and Cu^II^; pa = phthalate) [166]. The five MOFs were transformed into the M_x_S_y_@C composites with good incorporation metal sulfides via one-pot thermal treatment (650 °C, 3 h, N_2_ flow; MOF:S = 1:2). Among the three representative samples, i.e., Cu_2_S@C, Co_9_S_8_@C, and NiS_2_@C, NiS_2_@C had high S_BET_, large V_total_ (0.20 cm^3^ g^−1^), improved graphitization degree due to the catalysis by Ni species, and high theoretical capacitance of NiS_2_. Thus, the NiS_2_@C showed the best performance in a 3E configuration with 2 M KOH electrolyte. It exhibited ultra-high capacitances (806 F g^−1^ at 5 mV s^−1^ and 833 F g^−1^ at 0.5 A g^−1^) as well as good rate capabilities (422 F g^−1^ at 100 mV s^−1^).

### 4.4. Carbon/Polymer Composites

#### 4.4.1. Composites Containing Polyaniline

Poor electric conductivity and low cycling stability of polyaniline (PANI)-based electrode faces problems and limitations despite its many desirable properties, such as high capacitance, cost-effective synthesis, and chemical stability. Therefore, Zn-MOF-derived carbon/PANI composite was prepared to overcome these limitations [167]. As a first step, Zn-MOF precursor was hydrothermally synthesized from zinc (II) acetate dihydrate and 8-hydroxyquinoline. After one-step carbonization at 800 °C (8 h, N_2_ flow) followed by acid washing (4 M HCl), the Zn-MOF was converted into carbonized Zn-MOF. Then, the product composites of ZMP-X (X = mass of the carbonized Zn-MOF in g) were generated through the polymerization of aniline under suspension of the carbonized Zn-MOF. The optimized composite of ZMP-0.04 (MOF-PANI-0.04) contained hierarchical sandwich-like layered structure composed of the PANI for the redox transition and the carbonized Zn-MOF for conducting and protecting carbon skeleton. Therefore, the ZMP-0.04 yielded a high capacitance of 500 F g^−1^ in 1 M H_2_SO_4_.

To prevent self-stacking of layered carbonaceous structure, another type of composite with a 3D core/shell structure was synthesized from ZIF-8 precursor by Yamauchi et al. [168]. First, nanoporous carbon core was derived from ZIF-8 through direct carbonization at 800 °C (4 h, N_2_ flow) and HF washing. Next, multifaceted core/shell carbon/PANI composite of SN (N = polymerization time in h) was produced after the polymerization of aniline on a surface of nanoporous carbon polyhedrons. The 1D PANI nanorod arrays showed uniformly perpendicular growth on the polyhedral carbon core surface under optimization of polymerization time (3 h). The former is helpful for conductivity while the latter is good for ionic diffusion. As a result, the optimized S3 sample showed high performance under 1 M H_2_SO_4_ electrolytic condition. It exhibited an ultra-high capacitance of 1100 F g^−1^ at 5 mV s^−1^ and a good rate capacity of 57% retention at 200 mV s^−1^.

The ZIF-8 carbon precursor was introduced to an interconnected PANI fiber/C composite with a 3D cross-linked structure [169]. The ZIF-8 precursor was directly carbonized at 800 °C (8 h, N_2_ flow) and then washed with 4 M HCl. The carbonized ZIF-8 was transformed into N-doped porous carbon via KOH activation (weight ratio of the carbonized ZIF-8 to KOH = 1/3; 800 °C, 4 h, N_2_ flow) and acid treatment (1 M HCl). After the polymerization of aniline on the N-doped porous carbon, the interconnected composite of PANI fiber/N-doped porous carbon named 3CPC was obtained. In the 3CPC with high conductivity and N contents (11.5%), the N-doped carbon provided high surface area, and the PANI fiber offered structural stability as well as pseudocapacitance. Thus, in a 3E cell with 1 M H_2_SO_4_ electrolyte, it showed an ultra-high capacitance and excellent rate performance (618 F g^−1^ at 20 A g^−1^).

#### 4.4.2. Composite Containing PEDOT

There existed two N-doped porous carbon-based composites, i.e., Au@NC800 and NC800-PEDOT, to resolve the low capacitance or conductivity of ZIF-8-derived carbon (NC800) [170]. First, for the construction of the Au@NC800, AuCl_4_^-^@ZIF-8 suspended in an aqueous solution (Au/Zn = 0.1:1) was reduced by NaBH_4_, and the obtained Au@ZIF-8 was carbonized at 800 °C (N_2_ flow). For the preparation of the NC800-PEDOT, ZIF-8 was also carbonized at 800 °C (N_2_ flow) to produce the NC800, and the resultant NC800 was mixed with poly (3,4-ethylenedioxythiophene) nanotubes (PEDOT NTs) via sonication. Compared with bare NC800, the Au@NC800 had high ratio of (V_meso_+V_macro_)/V_micro_ (19.75), and the NC800-PEDOT contained large values of S_BET_ and V_total_ (1.55 cm^3^ g^−1^). In a 3E in 1 M NaCl electrolyte, the specific capacitances at 5 mV s^−1^ of NC800, NC800-PEDOT, and Au@NC800 were 155.8, 217.7, and 171.7 F g^−1^, respectively. In addition, at 0.1 A g^−1^, the specific capacitances increased in the order of NC800 < Au@NC800 < NC800-PEDOT. Furthermore, both Au@NC800 and NC800-PEDOT exhibited lower values of internal resistance (IR)-drop at 0.1 A g^−1^ and ESR from EIS data than NPC 800. These results suggested that the incorporation of Au NPs or PEDOT NTs into ZIF-8-derived carbon could improve the conductivity and electrochemical performance.

### 4.5. Carbon/LDH Composite

Metal-based layered-double-hydroxides (LDHs) have many useful properties. They are relatively cheap, environmentally friendly, and highly redox-active. However, their self-aggregation tendency and low electric conductivity can often prevent their use as electrode materials. Therefore, to overcome these limitations, ZIF-8-C@NiAl-LDH composite was prepared from ZIF-8 precursor [171]. The ZIF-8-C was synthesized from the carbonization of the ZIF-8 at 900 °C (2 h, Ar flow) and acid etching. In a successive step, the ZIF-8-C was coated with ultrathin layers of AlOOH primer sol, then the obtained ZIF-8-C/AlOOH was stirred with Ni (NO_3_)_2_∙6H_2_O and urea. The mixed precursors were hydrothermally treated to give the ZIF-8-C@NiAl-LDH composite. With the incorporation of 2D NiAl-LDH into 3D nanoporous carbon polyhedrons, the resultant core/shell composite had hierarchical porosity (micro-/meso-/macropores), compact interaction between core and shell, and synergistic structure of the redox-active shell and core to provide surface area, conductivity, and structural stability. The composite exhibited a very high capacitance of 1370 F g^−1^ at 1 A g^−1^ in 1 M KOH.

### 4.6. Composites from Single-Carbon Sources

The Co_3_O_4_ electrode had an ultra-high theoretical capacitance of 3560 F g^−1^, yet it showed low cycling stability and poor conductivity. Therefore, ZIF-67-derived carbon and graphene-like 2D MoS_2_ were employed as shell materials to enhance the electrochemical storage ability of Co_3_O_4_ through the formation of Co_3_O_4_/C@MoS_2_ core/shell composite [172]. Initially, the ZIF-67 was converted into Co/C through direct carbonization at 700 °C (5 h, N_2_ flow). The Co_3_O_4_/C was produced after calcination of Co/C at 270 °C (15 h, in air). Subsequently, the solvothermal treatment of Co_3_O_4_/C with (NH_4_)_2_MoS_4_ in DMF gave Co_3_O_4_/C@MoS_2_-n (n is the solvothermal treatment period in h). The optimized product of Co_3_O_4_/C@MoS_2_-20 (S_BET_ = 257 m^2^ g^−1^) contained hierarchical mesoporosity, highly stable structure, and good transfer abilities of electron and ion. As a result, it showed ultra-high capacitances of 1096 F g^−1^ at 5 mV s^−1^ and 1076 F g^−1^ at 1 A g^−1^ and outstanding rate performance of 840 F g^−1^ at 100 mV s^−1^ and 827 F g^−1^ at 10 A g^−1^ in 2 M KOH.

**Figure 12:**
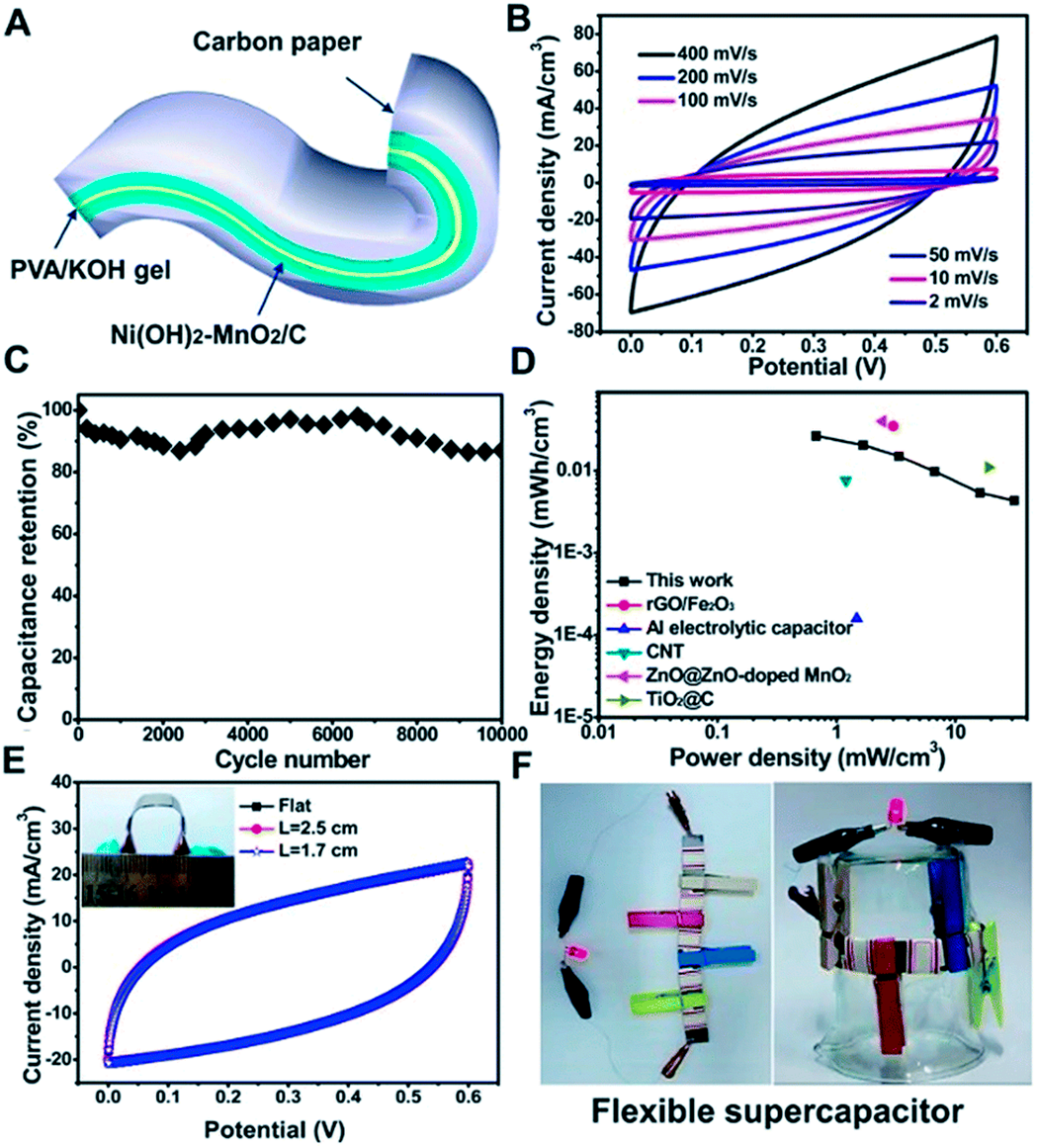
Electrochemical investigation of Ni (OH)_2_–MnO_2_/C composite-based flexible all-solid-phase supercapacitor cell. (**A**) Picture for the configuration of the assembled cell. (**B**) CV curves at various scan rates. (**C**) Cyclic performance at 11.2 mA cm^−3^. (**D**) Ragone plots. (**E**) CV curves at various bending conditions. Inset: photo for a bent cell. (**F**) Photos for a red LED brightened by tandem cells under the flat (left) and the bending condition (right) [173].

To prevent the self-agglomeration, 2D nanosheets of Ni (OH)_2_ and MnO_2_ were incorporated into 3D carbon matrix to generate 3D Ni(OH)_2_-MnO_2_/C composite with a hierarchically ordered structure [173]. The starting Ni-MOF precursor was synthesized at room temperature in an aqueous solution containing NiCl_2_∙6H_2_O, K_2_C_2_O_4_, and ethylene diamine. The annealed Ni-MOF precursor at 300 °C (1 h, N_2_ flow) was transformed into Ni@C via carbonization at 700 °C (2 h, N_2_ flow). Then, the Ni@C was hydrothermally oxidized with KMnO_4_ (150 °C, 24 h) to obtain the Ni (OH)_2_-MnO_2_/C composite. The resultant composite had a large accessible surface area from 2D Ni(OH)_2_ and MnO_2_ nanosheets, highly active faradaic redox centers (major reaction: Ni(OH)_2_ + OH^−^ ↔ NiOOH + H_2_O + e^−^, minor reaction: MnO_2_ + K^+^ ↔ MnOOK + e^−^), and improved electronic/ionic conductivities due to the synergistic effect between individual elements in 6 M KOH electrolyte. Therefore, this composite electrode delivered a capacitance of 862.0 F g^−1^, an energy density of 22.1 W h kg^−1^, and a power density of 430.0 W kg^−1^ at 2 A g^−1^ in 6 M KOH. In addition, at 40 A g^−1^, it yielded the respective values of 574.0 F g^−1^, 14.4 W h kg^−1^, and 8500.0 W kg^−1^. Moreover, using PVA/KOH gel electrolyte, solid-phase SSC with flexible and bendable stabilities showed a high volumetric capacitance of 530 mF cm^−3^ at a current density of 2.24 mA cm^−3^, and the highest energy density of 0.027 mW h cm^−3^ at a powder density of 0.672 mW cm^−3^ (Figure 12).

### 4.7. Composites from Dual-Carbon Sources

Hybrid ZIF-derived nanoporous functional composites (HZ-NPFCs) were synthesized via two-step thermal treatment of a Co^2+^ dominant hybrid Co/Zn-ZIF (HZ) [174]. With the introduction of a 2-fold higher amount of Co^2+^ ion than Zn^2+^ ion, the HZ precursor was prepared under mild conditions and subsequently transformed into hybrid ZIF-derived nanoporous carbon (HZ-NPC) through carbonization at 800 °C (5 h, N_2_ flow). The HZ-NPC had CNT and Co NPs. The HZ-NPC was further thermally oxidized in air for the generation of HZ-NPFC/X-Y (X = oxidation temperature in °C, Y = oxidation period in h). After optimization of the oxidation conditions (250 °C, 5 h), pseudocapacitive Co_3_O_4_, conductive elements (CNTs, CoNPs), and EDLC-active N-doped nanoporous carbon (NPC with micro-/mesoporosity) coexisted in HZ-NPFC/250-5. As a result, the HZ-NPFC/250-5 electrode operating in the 3E mode in 6 M KOH showed capacitances of 545 F g^−1^ at 2 A g^−1^ and 180 F g^−1^ at 10 A g^−1^.

Cotton textile with high surface area was a cheap and effective natural carbon precursor. Thus, the dual-carbon source of HKUST-1 and T-shirt-derived cotton textile (TS) were employed for the synthesis of a helical and tubular hierarchical carbon composite, which contained Cu and CuO_x_ (x = 1 or 2) [175]. Initially, a carbon hybrid template of HKUST-1@TS was fabricated by 80-cycled liquid phase epitaxial (LPE) coating of the HKUST-1 on the TS. The TS was transformed into helical carbon tube, and the HKUST-1 was converted into carbon film with incorporated Cu and CuO_x_ during one-step carbonization of the hybrid template. The obtained hierarchical carbon composite (HKUST-1@TS-800) had pseudocapacitive sites, i.e., Cu and CuO_x_, large electroactive surface area from the helical structure, and electrical conduction sites, i.e., Cu and tubular carbon structure. Therefore, in a 3E cell with 6 M KOH electrolyte, the HKUST-1@TS-800 showed areal capacitances of 1812 mF cm^−2^ at 1 mA cm^−2^ and 510 mF cm^−2^ at 20 mA cm^−2^.

Like most other metal oxide-based electrode materials, low electric conductivity and poor ionic transfer kinetics are frequently encountered in the highly cost-effective electrode materials based on manganese oxide. Thus, using the Mn-MOF precursor, poly(styrene-*co*-AA) (PSA) spherical template and GO stabilizer, the 3D hollow composite of Mn_3_O_4_@N-doped carbon/graphene (MCG) with ordered multilength scale porosity was prepared by two-step thermolysis [176]. The hybrid precursor of PSA@Mn-MOFs/GO-2 was prepared by ultra-sonification-agitation of the reaction mixture containing MnCl_2_, PSA, GO, and 2,3-pyridinedicarboxylic acid (2,3-pda), under controlled molar ratio (Mn^2+^/2,3-pda = 2). Then, the PSA@Mn-MOFs/GO-2 was carbonized at 500 °C (2 h, Ar flow) and subsequently stabilized in air at 150 °C (12 h) to form MCG-2. The obtained MCG-2 contained high N-dopant content (5.4 at%), pseudocapacitive activity from the quaternary N-Q site in carbon and Mn_3_O_4_, interconnected conducting system from N-doped carbon and graphene, ordered hollow porosity of N-doped carbon, and interlinked pores between Mn_3_O_4_ NPs and N-doped carbon. Therefore, the MCG-2 exhibited high capacitances.

A new hierarchical porous carbon composite (PMCP) by in situ polymerization of aniline on a carbon substrate for the preparation of integrated electrode with flexible and free-standing properties was synthesized via four-step treatment, i.e., thermal conversion, deposition, carbonization, and polymerization [177]. In the first step, electrospun polyamide acid (PAA) membrane was thermally transformed into polyimide (PI) nanofiber membrane. In the second step, ZIF-8 crystals were deposited on the electrospun PI nanofiber membrane. In the next step, the obtained PI/ZIF-8 membrane was carbonized to form PMC at 950 °C (5 h, N_2_ flow). In the final step, vertically oriented PANI NW arrays were uniformly grown on the PMC substrate through the polymerization of aniline. Therefore, the PMCP was composed of several ideal components for better electrochemical performance: (i) ZIF-8-derived carbon for well-defined porosity, (ii) carbon nanofiber for conductive, stable, flexible, free-standing, and self-interconnection properties, and (iii) PANI NWs for conductive and pseudocapacitive features. Additionally, in the PMCP, the PANI chains had an infiltrated structure with hierarchical porous carbon–carbon nanofiber architecture and strong π–π interaction with each other. As a result, PMCP-based working electrode showed good performances in both 1 M H_2_SO_4_ aqueous electrolytic 3E cell and PVA/H_2_SO_4_ gel electrolytic symmetric 2E cell. It delivered capacitances of 468.6 F g^−1^ at 0.1 A g^−1^ and 112 F g^−1^ at 5 A g^−1^ in the 3E cell. The flexible solid-phase SSC yielded a high volumetric capacitance of 1973 mF cm^−3^, good bending stability, and cycling stability.

A new preparation of the complex composite of CuO_x_@mC_700_@PANI@rGO started from Cu-btc MOF [178]. The MOF precursor was solvothermally synthesized and then carbonized at 700 °C (3 h, N_2_ flow) to form CuO_x_@mC_700_. The uniform octahedral CuO_x_@mC_700_ particles with an ultra-high Cu content (85%) was composed of the mesoporous carbon (mC_700_) and CuO_x_. Subsequently, well-arranged interfacial layers composed of PANI nanorods and rGO were formed on the surface of CuO_x_@mC_700_ composite after simultaneous treatments for polymerization and 3D-rGO incorporation. The former was beneficial for stability and pseudocapacitance while the latter was good for flexibility and conductivity. Thus, the resultant composite of CuO_x_@mC_700_@PANI@rGO showed high capacitances of 569.4 F g^−1^ at 0.5 A g^−1^ and 408.2 F g^−1^ at 5 A g^−1^.

### 4.8. The Asymmetic Supercapacitor (ASC)

The electrochemical performances of carbon composite-based ASCs are depicted in Table 11. The textural information of the electrodes is given together to compare the surface area and their electrochemical capacitive properties.

#### 4.8.1. Carbon/Carbon Composites

Using a flexible and conductive substrate of CNT film, a new binder-free ASC of CNT@NiCo-LDH//CNT@NC was fabricated from Co-MOF-derived working electrodes [179]. The positive electrode of Ni-Co-LDH on the CNT film (CNT@NiCo-LDH) was prepared by two steps. In the first step, Co-MOF (ZIF-67) was directly grown on the CNT substrate in aqueous medium to form CNT@Co-MOF nanosheets. In the second step, the CNT@Co-MOF was solvothermally treated in Ni (NO_3_)∙6H_2_O ethanol solution at 120 °C for 2 h to generate CNT@NiCo-LDH nanosheets. Also the negative electrode of N-doped carbon nanosheets on the CNT film (CNT@NC) was synthesized by carbonization of the CNT@Co-MOF at 500 °C (2 h, N_2_ flow) and a subsequent treatment with 3 M FeCl_3_. The CNT@NC-based working electrode for the 3E cell in 6 M KOH delivered capacitances of 278.8 F g^−1^ at 2 A g^−1^ and 82 F g^−1^ at 10 A g^−1^. The CNT@NiCo-LDH//CNT@NC ASC exhibited capacitances of 119.8 F g^−1^ at 1 A g^−1^ and 53.3 F g^−1^ at 10 A g^−1^. The respective energy and power densities were 37.4 W h kg^−1^ and 750 W kg^−1^. All Ni-Co-LDH, NC, and CNTs had synergistic effects on the electrochemical performance of the ASC. The hollow and porous structure of nano-sized Ni-Co-LDH contributed to surface area. The NC provided high surface area and good conductivity. The CNTs also enhanced the conductivity and flexibility.

Hu et al. synthesized ZIF-8-derived N-doped porous carbon materials (NPCs) and their modified composites functionalized with pseudocapacitive organic molecules [180]. The NPCs was prepared from ZIF-8 through carbonization at 800 °C (8 h, N_2_ flow) and acid etching (1 M HCl). The NPCs contained well-defined uniform shape of polyhedrons, high S_BET_, large V_total_ (0.57 cm^3^ g^−1^), micro-/mesoporosity, and good graphitization degree (*I*_D_/*I*_G_ = 0.98). Thus, the NPCs were used as host materials to incorporate the redox-active organic molecules (AQ = anthraquinone; NQ = 1,4-naphthoquinone; TCBQ = tetrachlorobenzoquinone). The pseudocapacitive organic guests were implanted into the NPCs via noncovalent interactions through a simple evaporation of solvent. Due to the variable redox potentials of the guest molecules in 1 M H_2_SO_4_ electrolyte (−0.09 V for AQ, 0.28 V for NQ, and 0.53 V for TCBQ vs. SCE), self-matching performance of potential could be displayed in the ASC of TN-NPCs//AQ-NPCs, where TN-NPCs was a positive electrode containing both NQ and TCBQ. The negative electrode of AQ-NPCs composite (AQ mass loading = 15.5%; D_pore_ = 2.96 nm) exhibited a capacitance of 373 F g^−1^ at 1 A g^−1^ in a 3E cell with a potential window of -0.4 ~ 0.2 V. The positive electrode of TN-NPCs (NQ mass loading = 12.3%; TCBQ mass loading = 1.2%; S_BET_ = 356 m^2^ g^−1^; D_pore_ = 2.40 nm) showed a capacitance of 392 F g^−1^ at 1 A g^−1^ with a potential window of 0.0 ~ 1.0 V. In the ASC, satisfactory H^+^ storage was achieved by similar porosity between the positive and negative electrodes. Moreover, the surface-controlled redox behavior was fast and reversible to facilitate the self-matching kinetics. Therefore, the ASC showed a capacitance of 86 F g^−1^ at 1 A g^−1^. The ASC delivered a superior maximum energy density of 23.5 W h kg^−1^ at 0.7 kW kg^−1^ compared to NPCs//NPCs (15.6 W h kg^−1^) and AC//AC (4.3 W h kg^−1^) SSCs.

#### 4.8.2. Carbon/Metal Oxide Composites

##### Composites Containing Cobalt Oxides

The Co-MOF, [Co_4_(phen)_4_Cl_8_] (phen = 1,10-phenanthroline), was used as a sole precursor for the preparation of C/Co-200//C-300 ASC [181]. The Co-MOF precursor was synthesized under reflux condition at 160 °C for 4 h with the help of PVP. Then, the positive electrode of C/CoO-200 was prepared by a single-step carbonization of the precursor at 200 °C (1 h, N_2_ flow). The resultant C/CoO-200 had mesoporous structure (D_pore_ = 5 nm, *I*_D_/*I*_G_ = 0.94), Cl/O-dopants (Cl = 1.9 at%, O = 9.4 at%), CoO NPs (Co = 1.9 at%) with no aggregation, and high carbon content (86.8 at%). The C/CoO-200 showed a good oxygen affinity, so the favorable OH^-^ adsorption could be displayed in the positive electrode. As a result, the C/CoO-200 electrode delivered large capacitances in 2 M KOH. In addition, the negative electrode was obtained by carbonization at 300 °C (1 h, N_2_ flow) and subsequent acid treatment (3 M HCl). The product of C300 (*I*_D_/*I*_G_ = 0.99) also contained mesoporosity (D_pore_ = 4 nm), O-dopant (13.4 at%), high carbon content (86.6 at%), and a good affinity for oxygen. Therefore, the C300 showed capacitances of 207 F g^−1^ at 0.5 A g^−1^ and 81 F g^−1^ at 10 A g^−1^ under the same conditions. Finally, the ASC of C/Co-200//C-300 demonstrated capacitances of 92 F g^−1^ at 0.5 A g^−1^ and 64 F g^−1^ at 10 A g^−1^. The energy and power densities were 25.04 W h kg^−1^ at 350 W kg^−1^ and 17.4 W h kg^−1^ at 7000 W kg^−1^.

The ASC of Co@Carbon//Co_3_O_4_@Carbon was assembled from the Co-bdc nanosheet-derived carbons [182]. The Co-bdc nanosheet self-sacrificing template was prepared by a layering method using H_2_bdc and Co (CH_3_COO)_2_∙4H_2_O dissolved in DMF and CH_3_CN, respectively. Subsequently, this Co-bdc precursor with uniform morphology of parallelogram slice was transformed into the positive electrode of the ASC (Co@Carbon) via one-step carbonization at 700 °C (2 h, N_2_ flow). In addition, the precursor was converted to the negative electrode of the ASC (Co_3_O_4_@Carbon) through a single-step pyrolysis at 400 °C (1 h, O_2_ flow). Both electrodes had well-controlled hierarchical micro-/mesoporosity, uniform loading of Co-based NPs without aggregation, ultrathin carbon layer with laminated structure, and effective interfacial interaction between the carbon and Co-based NPs. As a result, good electrochemical performances of both electrodes were displayed in 6 M KOH electrolyte. In a 3E cell, the Co@Carbon (D_pore_ = 9.6 nm) delivered capacitances of 109 F g^−1^ at 0.25 A g^−1^ and 52 F g^−1^ at 7 A g^−1^. Moreover, in the similar cell, the Co_3_O_4_@Carbon showed capacitances of 261 F g^−1^ at 1 A g^−1^ and 50 F g^−1^ at 10 A g^−1^ due to efficient conducting network between the carbon and Co_3_O_4_ as a pseudocapacitive center. The ASC of Co@Carbon//Co_3_O_4_@Carbon showed capacitances of 28.2 F g^−1^ at 0.5 A g^−1^ and 8.9 F g^−1^ at 4 A g^−1^. The high voltage window of 1.5 V for the ASC was attributed to the ultrathin porous carbon layer.

Guo et al. reported ASCs of AC//Co-ZIF-X (X = carbonization temperature in °C) [183]. The Co-ZIF nanocuboids were grown on carbon cloth via chemical bath deposition. The ZIF-67 nanocuboids were transformed into Co-ZIF-X by single-step carbonization (1 h under N_2_). In the case of Co-ZIF-450, conductive Co metal and redox-active cobalt oxides (CoO and Co_3_O_4_) were effectively incorporated into nanoporous carbon networks. As a result, the Co-ZIF-450 electrode showed the best electrochemical properties among the Co-ZIF-X in 2 M KOH. The Co-ZIF-450 exhibited good areal capacitances: 1177 mF cm^−2^ at 1 mA cm^−2^ and 640 mF cm^−2^ at 20 mA cm^−2^. The ASC of AC//Co-ZIF-450 had excellent volumetric capacitive properties: 4 F cm^−3^ at 1mA cm^−2^, 2.15 F cm^−3^ at 40 mA cm^−2^, 1.32 mW h cm^−3^ at 9.4 mW cm^−3^, and 0.73 mW h cm^−3^ at 376 mW cm^−3^.

##### Composites Containing Iron Oxides

**Figure 13:**
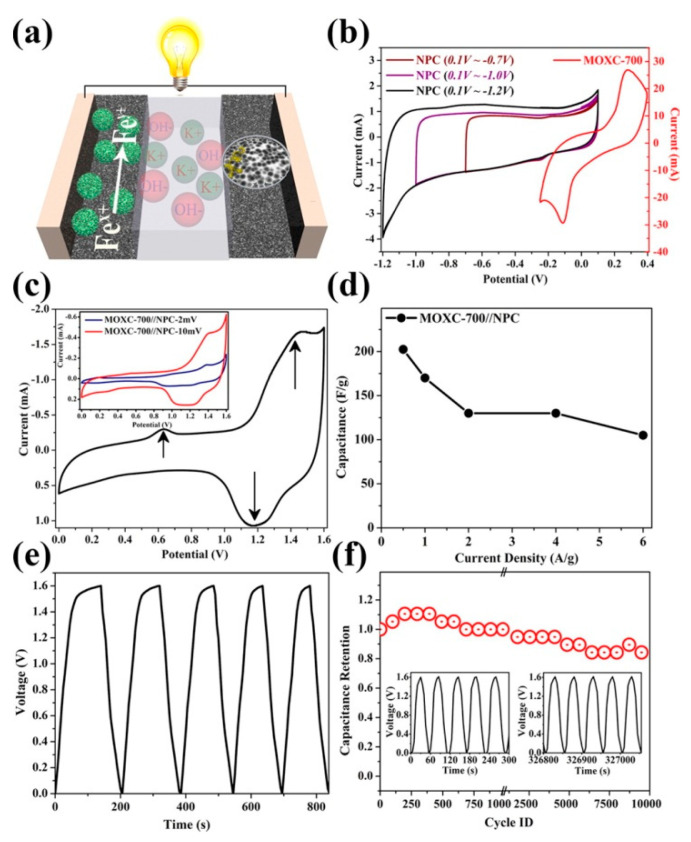
Schematic explanation of MOXC-700//NPC device in 6 M KOH electrolyte: (**a**) view of assembly, (**b**) comparative CV curves of positive and negative electrodes, (**c**) CV curves of the device, (**d**) capacitances of the device at different current loads, (**e**) charge−discharge curve of the device for the first five cycles, and (**f**) cycling performance for the device [184].

Dual precursors of btc-based MOF xerogels were used for the delicate and scalable synthesis of Fe_3_O_4_/Fe/C//NPC ASC (Figure 13) [184]. First, the polymerized MOF clusters formed nano-MOF particles (nMOFP). In addition, the MOF xerogels containing hierarchical porosity were formed by hetero-aggregated nMOFP. The MOX-Fe xerogel was prepared from MIL-100(Fe) and the MOX-Al xerogel from MIL-100(Al). MIL-100(Fe)-derived MOX-Fe was chosen as a precursor for the positive electrode due to appropriate features of Fe element, such as natural abundance, cheap cost, high conductivity, and redox-active properties. The positive electrode of MOXC-700 (N = 3.97 at%) was simply prepared via direct carbonization of the MOX-Fe xerogel at 700 °C (5 h, Ar flow). The resultant MOXC-700 was composed of thin carbon layer with hierarchical porosity (D_pore_ = 1.2 and 12 nm) and uniformly dispersed Fe_3_O_4_/Fe NPs. The carbon layer played a role of maintaining structural stability and the Fe_3_O_4_/Fe NPs were beneficial for electrochemical performance due to their redox activities of Fe/Fe^2+^ and Fe^2+^/Fe^3+^. Therefore, the MOXC-700 exhibited excellent electrochemical performance (600 F g^−1^ at 1 A g^−1^, 500 F g^−1^ at 8 A g^−1^) and outstanding cycling stability. The negative electrode of NPC was prepared by carbonization of the MOX-Al at 1000 °C (5 h, Ar) and subsequent 6 M KOH solution-driven green activation. The obtained 3D NPC (V_pore_ = 1.95 cc g^−1^, N = 1%) had hierarchically interconnected porous structure (D_pore_ = 1.2 and 5.8 nm). As a result, it showed high rate capability with capacitances of 272 F g^−1^ at 2 mV s^−1^ and 207 F g^−1^ at 250 mV s^−1^. The ASC of MOXC-700//NPC was fabricated with a controlled mass ratio of m_+_/m_-_ = 0.42, and the cell operated effectively in 6 M KOH electrolyte. It delivered capacitances of 170 F g^−1^ at 1 A g^−1^ and 105 F g^−1^ at 6 A g^−1^.

A novel Fe_2_O_3_-based anode material for fiber shaped ASC was reported [185]. Due to low conductivity and poor ionic diffusion rate, a simple Fe_2_O_3_-based electrode had limitations to practical application despite its many favorable properties. Fe_2_O_3_ is a cheap, green, and highly abundant resource with high theoretical capacitance and broad operating voltage window. Therefore, S-α-Fe_2_O_3_@C composite derived from MIL-88-Fe was used as an anode for ASC of S-α-Fe_2_O_3_@C/OCNTF//Na-MnO_2_ NSs/CNTF. The oxidized carbon nanotube fibers (OCNTFs) were prepared by electrochemical oxidation of CNTFs (0.8 M H_2_SO_4_, 1 ~ 2 V, 25 mV s^−1^, 15 cycles) and used as a substrate for anode. CNTFs contain light weight, flexibility, good conductivity, and mechanical robustness. After that, the MIL-88-Fe was grown on the substrate and then pyrolyzed in air at 350 °C (2 h) to form the S-α-Fe_2_O_3_@C/OCNTF. The S-α-Fe_2_O_3_@C showed spindle-like structure and mesoporosity (D_pore_ = 19.2 nm). As the result of improved interfacial compatibility between S-α-Fe_2_O_3_@C and OCNTF, the S-α-Fe_2_O_3_@C/OCNTF yielded a large areal capacitance of 1232.4 mF cm^−2^ at 2 mA cm^−2^ and 63% rate capacity at 20 mA cm^−2^ in 1 M Na_2_SO_4_ electrolyte. Furthermore, the ASC of S-α-Fe_2_O_3_@C/OCNTF//Na-MnO_2_ showed good performance in Na_2_SO_4_-CMC gel electrolyte (CMC = carboxymethyl cellulose sodium). It delivered areal capacitances of 201.3 mF cm^−2^ at 2 mA cm^−2^ and 136.4 mF cm^−2^ at 20 mA cm^−2^. The areal energy and power densities were 135.3 μW h cm^−2^ at 2199.9 μW cm^−2^, and 91.7 μW h cm^−2^ at 21998.4 μW cm^−2^.

ZIF-8 and ZIF-67 were employed as starting precursors for the electrode materials of CF@NiCo-A-S//Fe_x_O_y_@CNS ASC by Shi et al. [186]. First, 2D ZIF-8 precursor was transformed into carbon nanosheets (CNS) via direct carbonization at 930 °C (2 h, Ar flow). After that, the CNS were mixed with (NH_4_)_2_Fe (SO_4_)_2_/DMF solution and followed by a brief calcination at 550 °C (30 min, Ar flow) to produce Fe_x_O_y_@CNS. The Fe_x_O_y_@CNS electrode yielded an energy density of 30.3 mA h g^−1^ at 1 A g^−1^ and 66% rate retention (20.6 mA h g^−1^) at 20 A g^−1^ in a 3E cell with 3 M KOH electrolyte. The fabrication of cathode material was started from ZIF-67 crystal growth on carbon fibers (CF@Co-MOF). Then, the CF@Co-MOF was ion-exchanged with Ni (NO_3_)_2_∙6H_2_O and thermally treated with at 120 °C (2 h) to produce CF@NiCo-LDH. After that, the CF@NiCo-LDH nanosheets were converted into CF@NiCo-A through a reduction process at 250 °C (30 min, 10 vol% H_2_/Ar flow). Finally, the cathode of CF@NiCo-A-S was prepared by the hydrothermal vulcanization of CF@NiCo-A in the presence of TAA at 120 °C for 3 h. In the same electrolyte, the ASC of CF@NiCo-A-S//Fe_x_O_y_@CNS showed energy densities of 16.8 mA h g^−1^ at 1 A g^−1^, 11.3 mA h g^−1^ at 10 A g^−1^, 48.2 W h kg^−1^ at 840.0 W kg^−1^, and 32.3 W h kg^−1^ at 8300.0 W kg^−1^.

##### Composites Containing Manganese Oxides

Manganese oxides also possess many favorable features to enhance electrochemical performance, such as high theoretical capacitance, cheap cost, and natural abundance. Nevertheless, they usually showed poor rate and cycling performance owing to unfavorable characteristics, such as low conductivity, large volume change, and partial dissolution in electrolytes. In this sense, Mn-bdc-derived composite of MnO_x_-CSs-600 was used as both working electrode of the 3E cell and the cathode of ASC in 1.0 M Na_2_SO_4_ electrolyte [187]. The Mn-bdc MOF precursor was synthesized from DMF solution containing Mn(Ac)_2_∙6H_2_O and H_2_bdc with the help of PVP at room temperature. It was converted to the MnO_x_-CSs-600 via one-step carbonization at 600 °C (2 h, 5 vol% H_2_/Ar flow). In the obtained composite with S_BET_ of 182.3 m^2^ g^−1^, numerous MnO_x_ particles (Mn_3_O_4_: MnO = 70.6:29.4) were evenly distributed throughout the 2D ultrathin carbon sheet architecture without self-aggregation. As a result, the enhancements of electrical conductivity, structural stability, and defects formation could be accomplished by strong combined effects of each element in the composite. The working electrode showed capacitances of 220 F g^−1^ at 1 A g^−1^ and 106 F g^−1^ at 8 A g^−1^. MnO_x_-CSc-600//AC ASC delivered capacitances of 61.1 F g^−1^ at 0.25 A g^−1^ and 26.7 F g^−1^ at 6 A g^−1^.

The MOF-derived nanoporous carbon composite (MOF-NPC/MnO_2_) was used for the fabrication of an ASC by Zhao et al. [188]. The best negative electrode of MNC950 was synthesized via carbonization of ZIF-8 at 950 °C (5 h, N_2_ flow) and HCl washing. In addition, the best positive electrode of MNCMn60 was prepared by the reaction of MNC950 with KMnO_4_ solution for 60 min and subsequent H_2_O washing. Although MnO_2_ nanodots were confined in the pores of MNC950, both electrodes showed similar surface areas, 920 m^2^ g^−1^ for MNC950 and 906 m^2^ g^−1^ for MNCMn60. As a working electrode for the 3E cell in 1 M H_2_SO_4_ electrolyte, the MNCMn60 showed better maximum specific capacitance of 163 F g^−1^ than that of MNC950 (146 F g^−1^) due to additional pseudocapacitance provided by the MnO_2_. It also exhibited ultra-high rate performance up to 5 A g^−1^ (120 F g^−1^). The ASC of MNCMn60//MNC950 operated in the same electrolyte with appropriate mass ratio (MNCMn60:MNC950 = 1:0.90).

Mn-btc was introduced for the synthesis of Mn_2_O_3_/C composite [189]. The Mn-btc was transformed into the Mn_2_O_3_/C composite via a single-step carbonization at 500 °C (2 h). The obtained Mn_2_O_3_/C (V_total_ = 0.2442 cm g^−1^, D_pore_ = 9.5 nm) contained interconnected porous network ideal for electron transfer and generation of electrochemical active sites, rectangular bar-shaped microstructure, and effective carbon coating on the Mn_2_O_3_ particles for prevention of dissolution and contribution of ionic adsorption. The composite yielded capacitances of 553 F g^−1^ at 5 mV s^−1^ and 273 F g^−1^ at 50 mV s^−1^, 776 F g^−1^ at 1 A g^−1^, and 125 F g^−1^ at 20 A g^−1^ in 1.0 M Na_2_SO_4_ electrolyte. The ASC of AC//Mn_2_O_3_/C was operable in the same electrolyte with a controlled mass ratio of m_+_/m_−_ = 3.936. It delivered capacitances of 166 F g^−1^ at 5 mV s^−1^, 87 F g^−1^ at 50 mV s^−1^, 122 F g^−1^ at 2.5 A g^−1^, and 28 F g^−1^ at 25 A g^−1^.

[Mn(1,4-ndc)] _n_ was used as a precursor for anode of Li-HEC [190]. The precursor was hydrothermally synthesized from MnCl_2_∙4H_2_O and 1,4-ndc in the basic KOH solution at 180 °C (72 h). Subsequently, it was converted to MnO_2_@C-NS via direct carbonization at 800 °C (6 h, N_2_ flow). The resultant MnO_2_@C-NS was consisted of homogeneously dispersed MnO_2_ nanocrystals (45 wt.%) and ultrathin carbon nanosheets with graphene-like structure with porosity and conductivity (S_BET_ = 45 m^2^ g^−1^, S_Langmuir_ = 65 m^2^ g^−1^). The carbon nanosheets acted as a structural substrate. Moreover, it showed large V_pore_ (1.09 cm^3^ g^−1^), laminated structure, and meso-/macroporosity as well. The precursor of cathode (K-MOF) was prepared from btc, KNO_3_, and NH_4_F. Then, the cathode of 2D ultrathin nanoporous carbon nanosheets (NPCS) was obtained by single-step carbonization at 800 °C (8 h, N_2_) and acid etching (5 wt.% HCl). The cathode had 2D-layered structure, conductive network, high S_BET_, and large V_pore_ (1.06 cm^3^ g^−1^). Thus, under 1 M LiPF_6_ in EC/DMC (1:1) electrolyte, the Li-HEC of MnO_2_@C-NS//NPCS showed energy and power densities of 129 mA h g^−1^ (~124 F g^−1^) at 0.45 A g^−1^, 166 W h kg^−1^ at 550 W kg^−1^, 49.3 W h kg^−1^ at 3.9 kW kg^−1^.

##### Other Kinds

Nb_2_O_5_ is a promising anode material for Li-HEC due to its high theoretical capacity (~200 mA h g^−1^), long-term cycling stability, and fast rate of charge. Nonetheless, its application was restricted by poor electric conductivity (3 × 10^−6^ S cm^−1^) and difficulty in controlling the crystal structure. Thus, Liang et al. reported a new anode material of NQD-NC, which was composed of orthorhombic phase of Nb_2_O_5_ quantum dots (QDs) and ZIF-8-derived N-doped porous carbon (NC) [191]. The NC was prepared by carbonization of rhombic dodecahedral ZIF-8 crystals at 900 °C (4 h, Ar flow) and acid etching (2 M HCl). The NC was introduced to niobium oxalate aqueous solution, and then hydrothermally transformed at 180 °C for 12 h. Subsequently, one-step carbonization of the hydrothermally synthesized product at 700 °C (4 h, Ar flow) generated the NQD-NC. The obtained NQD-NC (C = 10 wt.%, N = 1.23 wt.%) had microporosity (D_pore_ = 1~2 nm), mesoporosity, high S_BET_ (268 m^2^ g^−1^), large V_pore_ (0.413 cm^3^ g^−1^), and Li-insertion center for the following reaction: Nb_2_O_5_ + *x*Li^+^ + *x*e^-^ ↔ Li*_x_*Nb_2_O_5_. With the controlled mass ratios (NQD-NC/AC = 2.2 in 0.5~3.0 V, 5.5 in 0.5~4.0 V), Li-HEC of NQD-NC//AC was operable in EC/DMC (1:1) electrolyte containing 1.0 M LiPF_6_. When the operating potential range was 0.5~3.0 V, the Li-HEC exhibited an energy density of 51.4 W h kg^−1^ at 350 W kg^−1^, and a power density of 8750 W kg^−1^ at 16.3 W h kg^−1^. When the potential range was extended to 0.5~4.0 V, the NQD-NC//AC showed an energy density of 76.9 W h kg^−1^ at 450 W kg^−1^ and a power density of 11,250 W kg^−1^ at 22.4 W h kg^−1^. Notably, the Li-HEC device could turn on a 3 V blue LED for more than 12 min. In the NQD-NC, the Nb_2_O_5_ QDs facilitated the rates of Li^+^ diffusion and electron transport.

Yamauchi et al. fabricated a new ASC of NiCo_2_O_4_-NC//NC [192]. The nanoporous carbon (NC) with 3D polyhedral shape was synthesized via direct carbonization of ZIF-8 at 900 °C (5 h, N_2_ flow) and followed by etching by 10 wt.% HF. The resultant NC was a good source for composite because of its mechanical stability, interconnected conducting pathways, and regular shape. For the preparation of the NiCo_2_O_4_-NC, the NC was treated with c-H_2_SO_4_ to form hydrophilic S-NC. Then, the S-NC was transformed into sheet-like core/shell precursor of NiCo_2_O_4_-NC under reflux conditions with the mixture containing Ni(NO_3_)_2_∙6H_2_O, Co(NO_3_)_2_∙6H_2_O, hexamethylenetetramine (HMT), and trisodium citrate (TSC, modulator). After that, the positive electrode of NiCo_2_O_4_-NC was obtained by pyrolysis of the precursor at 300 °C (3 h, air flow). As a result, the 2D NiCo_2_O_4_ nanosheets was successfully incorporated into the hierarchically structured composite. The 2D NiCo_2_O_4_ nanosheets were cheap conductors and had a large accessible surface area for high electrochemical activity (V_pore_ = 0.53 cm^3^ g^−1^). Furthermore, the composite had good mesoporosity (3~10 nm) and favorable synergetic interaction between 3D NC core and 2D NiCo_2_O_4_ shell. Therefore, NiCo_2_O_4_-NC yielded good electrochemical properties in 1 M KOH basic electrolyte (NiCo_2_O_4_ + OH^−^ ↔ NiOOH + 2CoOOH + 2e^−^, CoOOH + OH^−^ ↔ CoO_2_ + H_2_O + e^−^). In a 2E cell, the ASC of NiCo_2_O_4_-NC//NC delivered capacitances of 89 F g^−1^ at 0.1 A g^−1^ and 26 F g^−1^ at 10 A g^−1^.

**Figure 14:**
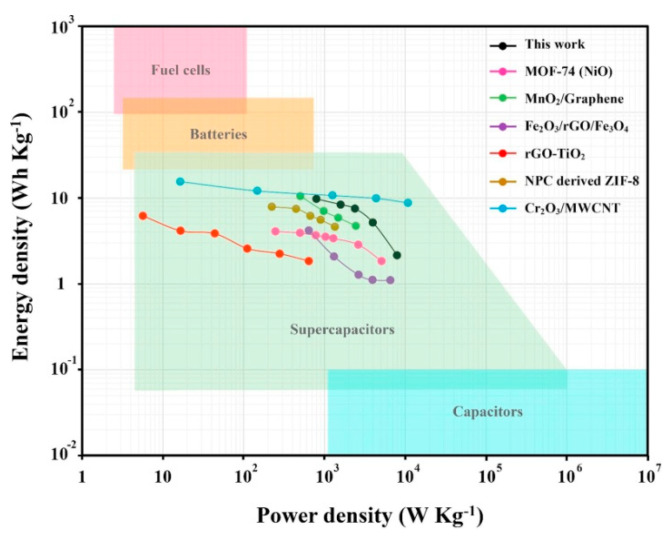
Ragone plots of Cr_2_O_3_/C//Fe_x_O_y_/C and other metal oxide/carbon composite-based ASCs [193].

Moradi et al. reported a new ASC of Cr_2_O_3_/C//Fe_x_O_y_/C to overcome the limitations of general metal-oxide-based electrodes, such as low conductivity and inferior cycling and rate performance [193]. The MIL-101 (Fe) was hydrothermally synthesized using Cr (NO_3_)_2_∙9H_2_O, H_2_bdc, and 40% HF at 220 °C (8 h). Then, it was converted into the Cr_2_O_3_/C cathode via a single-step carbonization at 800 °C (5 h, Ar flow). The resultant Cr_2_O_3_/C with S_BET_ of 59.9 m^2^ g^−1^ showed capacitances of 426 F g^−1^ at 5 mV s^−1^ in 6 M KOH electrolyte. Additionally, MIL-101(Fe) was solvothermally prepared from the mixture of FeCl_3_∙6H_2_O and H_2_bdc (110 °C, 20 h), and subsequently transformed into the Fe_x_O_y_/C anode through single-step carbonization at 800 °C (2 h, Ar flow). The obtained Fe_x_O_y_/C with S_BET_ of 67.6 m^2^ g^−1^ showed a capacitance of 114 F g^−1^ at 2 A g^−1^ in the same cell conditions. In the same electrolyte, the Cr_2_O_3_/C//Fe_x_O_y_/C ASC exhibited capacitances of 27.2 F g^−1^ at 1 A g^−1^ and 14.4 F g^−1^ at 5 A g^−1^. The Ragone plots are shown in Figure 14, and the performances of Cr_2_O_3_/C//Fe_x_O_y_/C ASC are better than other reported systems.

#### 4.8.3. Composites Containing Carbon/Metal Sulfide

##### Composites Containing Co_9_S_8_

Co_9_S_8_-containing composite with the inclusion of 2D CoNi alloys NPs into S/N-co-doped carbon nanosheets (CoNi@SNC) was fabricated from the 2D Co/Ni-MOF nanosheets [194]. The S,N-containing Co/Ni-MOFs were synthesized under mild basic condition at room temperature using mixed metal sources (CoCl_2_∙6H_2_O and NiCl_2_∙6H_2_O) and two different bridging linkers (tdc = thiophene-2,5-dicarboxylate, 4.4′-bpy = 4,4′-bipyridine). The as-prepared Co/Ni-MOFs with the same molar ratio of Co^2+^ and Ni^2+^ were directly carbonized to form the CoNi@SNC at 550 °C (2 h, N_2_ flow). The obtained CoNi@SNC (V_pore_ = 0.508 cm^3^ g^−1^) had uniform mesoporosity (D_pore_ = 3.0 and 4.0 nm) and good conductivity from graphitic carbon and the CoNi alloy NPs. Interestingly, the surfaces of CoNi alloy NPs could be partially transformed into electrochemically active sites in an alkaline electrolytic environment by forming either Co_3_O_4_/NiO or Co (OH)_2_/Ni(OH)_2_. As a result, the CoNi@SNC working electrode exhibited very high capacitances of 1970 F g^−1^ at 10 A g^−1^ and 1282 F g^−1^ at 20 A g^−1^ in 6.0 M KOH. Thus, this cell was very stable and highly active. Moreover, it also showed great improvement of capacitance from 972 to 1660 F g^−1^ after 1000 cycles at 2.0 A g^−1^. In the same electrolyte, the ASC of CoNi@SNC//AC showed capacitances of 156.7 F g^−1^ at 1 A g^−1^ and 109.3 F g^−1^ at 10 A g^−1^.

DUT-58-derived composite of Co_9_S_8_/NS-C with Co_9_S_8_ NPs and S,N-co-doped porous graphitic carbon wall was synthesized to solve the low conductivity and stability problems of Co_9_S_8_ electrode [195]. The environmentally friendly Co_9_S_8_ has a high theoretical capacitance, low cost, and high natural abundance. The precursor of DUT-58 was solvothermally synthesized from the mixture containing Co(NO_3_)_2_∙6H_2_O, 4,4′-biphenyldicarboxylic acid (4,4′-bpdc), and 1,3-bis(imidazol-1-yl)benzene (1,3-bib). The Co_9_S_8_/NS-C was produced by the carbonization of TAA-encapsulated DUT-58 at 700 °C (2 h, N_2_ flow). After that, the optimized product of Co_9_S_8_/NS-C-1.5h was generated via CO_2_ treatment (600 °C, 1.5 h). The resultant Co_9_S_8_/NS-C-1.5h (V_pore_ = 0.136 cm^3^ g^−1^) showed good mesoporosity (D_pore_ = 8.2 nm) and great interfacial contact between graphitic carbon (*I*_D_/*I*_G_ = 0.98) and pseudocapacitive Co_9_S_8_ (Co_9_S_8_ + 9OH^-^ ↔ Co_9_S_8_(OH)_9_ + 9e^-^). Therefore, the Co_9_S_8_/NS-C-1.5h electrode exhibited high capacitances of 734.09 F g^−1^ at 1 A g^−1^ and 653.64 F g^−1^ at 10 A g^−1^. The 3E cell was stable enough to show 99.8% capacitance retention at 10 A g^−1^ up to 140,000 cycles in 6 M KOH. In the same electrolyte, the ASC of Co_9_S_8_/NS-C-1.5h//AC delivered capacitances, energy and power densities of 75.59 F g^−1^ at 1 A g^−1^, 32.41 F g^−1^ at 10 A g^−1^, 14.85 W h kg^−1^ at 681.82 W kg^−1^, and 6.63 W h kg^−1^ at 6818.18 W kg^−1^.

The ASC of Co_9_S_8_@C//AC was also assembled with an actual mass loading of electrode more than 10 mg cm^−2^ [196]. The Co_9_S_8_@C-500 positive electrode was produced by thermolysis of Co-btc MOF with sulfur powder at 500 °C (2 h, 5% H_2_/Ar flow) and additional heat treatment at 300 °C (10 h). The Co_9_S_8_@C-500 showed 3D porous honeycomb-like structure (S_BET_ = 62.95 m^2^ g^−1^, V_pore_ = 0.26 cm^3^ g^−1^) and multilength scale porosities. The pseudocapacitive Co_9_S_8_ NPs buried in the conducting and structural carbon support were found in the Co_9_S_8_@C-500 composite (Co_9_S_8_ + 11OH^−^ ↔ Co_9_S_8_(OH)_11_ + 11e^−^, Co_9_S_8_(OH)_11_ + 9OH^−^ ↔ Co_9_S_8_O_10_ + 10H_2_O + 9e^−^). As a working electrode of the 3E cell in 2.0 M KOH electrolyte, the composite yielded large capacitances of 1887 F g^−1^ at 1 A g^−1^ and 1182 F g^−1^ at 10 A g^−1^. The Co_9_S_8_@C//AC ASC assembled with a controlled mass ratio (m_+_/m_−_ = 0.3) operating in the same electrolyte exhibited a capacitance of 166 F g^−1^ at 1 A g^−1^.

##### Composites Containing Nickel-Cobalt-Sulfides

Lin et al. synthesized Ni-Co-S@G from the activated nickelocene/ZIF-67 composite via single-step pyrolysis at 600 °C (2 h, Ar flow, mass ratio of S/composite = 5) to improve rate performance and cycling stability of nickel-cobalt sulfides [197]. The obtained Ni-Co-S@G (S_BET_ = 42.5 m^2^ g^−1^) was composed of a thin-layered graphene shell and various Ni-Co-S NPs (NiCo_2_S_4_, Ni_4_S_3_, Co_4_S_3_, and so on). The graphene shell was formed due to the catalysis by Co and Ni. The Ni-Co-S@G had interconnected framework architecture, uniform core/shell polyhedral structure, and mesoporosity (D_pore_ = 3.9 and 11 nm). The pseudocapacitive behavior of the Ni-Co-S could be effectively displayed in basic electrolyte aided by interfacial binding with conductive and protective graphene layer through the following reactions: CoS + OH^−^ ↔ CoSOH + e^−^, CoSOH + OH^−^ ↔ CoSO + H_2_O + e^−^, NiS + OH^−^ ↔ NiSOH + e^−^. As a result, in a 3E cell with 6 M KOH electrolyte, the Ni-Co-S@G electrode showed excellent performances: 1463 F g^−1^ at 1 A g^−1^, 750 F g^−1^ at 10 A g^−1^, and 12.6% capacitance loss after 1000 cycles at 17 A g^−1^. Moreover, the all-solid-phase ASC of Ni-Co-S@G//AC by using PVA/KOH electrolyte and flexible Ni film substrate also showed good performances: 217.8 F g^−1^ at 1 A g^−1^, 101.6 F g^−1^ at 10 A g^−1^, 51.0 W h kg^−1^ at 650.3 W kg^−1^, and 12.7 W h kg^−1^ at 11,700 W kg^−1^.

The hollow spherical composites of Ni-Co-S-n/NC (NC = N-doped carbon, n = Ni/Co molar ratio) were also reported [198]. The Ni-Co-btc-n with spherical morphology was solvothermally synthesized in the presence of PVP. The co-carbonization of melamine and Ni-Co-BTC-n (mass ratio = 20/1) at 450 °C (5 vol% H_2_/Ar flow) was performed to produce the Ni-Co-n/NC. The Ni-Co-n/NC composite was hydrothermally sulfidated with TAA (mass ratio of TAA/composite = 4) at 140 °C for 6 h. The optimized sample of Ni-Co-S-0.5/NC contained mesoporosity (N = 4.96 wt.%, S_BET_ = 10.27 m^2^ g^−1^), graphitic N-doped carbon (55.2 wt.%), and Ni-Co-S NPs (Co_9_S_8_ and NiCo_2_S_4_). The reversible battery-type faradaic reactions, i.e., MS//MSOH and MSOH//MSO where M is Ni and Co ions, were supported by the stable and conductive NC. The Ni-Co-S-n working electrode yielded a specific capacity of 519.6 C g^−1^ at 1 A g^−1^, 366.0 C g^−1^ at 20 A g^−1^, and good cycling performance after 2000 cycles in a 3E cell with 3 M KOH. Moreover, the Li-HEC of Ni-Co-S-0.5/NC//AC acting on 1 M LiPF_6_ in EC/DMC/DEC (1:1:1) electrolyte showed a capacitance of 111.2 F g^−1^ at 1 A g^−1^ and good rate performance (68.5% retention at 10 A g^−1^).

A new composite electrode of CC/CNWAs@Ni@CoNi_2_S_4_ (CNWAs = carbon nanowall arrays) with hierarchical structure was fabricated [199]. The vertical growth of ZIF-67 crystals on the free-standing CC substrate was performed via solution method. Neither binder nor conducting agents were used during the synthesis. The resultant CC/ZIF-67 was directly carbonized at 800 °C (2 h, 5 vol% H_2_/Ar flow) to produce carbon nanowall arrays on CC (CC/CNWAs). Subsequently, thin Ni layer was electrodeposited on the CC/CNWAs using NiSO_4_ and NH_4_Cl for the construction of CC/CNWAs@Ni. Additional electrodeposition on the CC/CNWAs@Ni was conducted by using CoCl_2_∙6H_2_O, NiCl_2_∙6H_2_O, and thiourea to obtain the CC/CNWAs@Ni@CoNi_2_S_4_ composite. In the composite, the Ni layer could act as an interfacial conducting bridge between CNWAs and CoNi_2_S_4_. Thus, the faradaic reaction center of mesoporous CoNi_2_S_4_ was effectively activated in a basic electrolyte: CoNi_2_S_4_ + 2OH^−^ ↔ CoS_2x_OH + Ni_2_S_4-2x_OH + 2e^−^. Therefore, the CC/CNWAs@Ni@CoNi_2_S_4_ electrode demonstrated ultra-high capacitances of 3163 F g^−1^ at 1 A g^−1^ and 2825 F g^−1^ at 5 mV s^−1^, and good rate performances (1503 F g^−1^ at 40 A g^−1^ and 1500 F g^−1^ at 50 mV s^−1^). Moreover, in the same electrolyte, the ASC of CC/CNWAs@Ni@CoNi_2_S_4_//AC exhibited good performances: 151.3 F g^−1^ at 1 A g^−1^, 73.1 F g^−1^ at 30 A g^−1^, 53.8 W h kg^−1^ at 800 W kg^−1^, and 32.2 W h kg^−1^ at 8000 W kg^−1^.

Recently, Zhou et al. reported another new hierarchical composite electrode of NiCo_2_S_4_-Ni_9_S_8_-DYMs (DYMs = double-layered yolk-shell microspheres) from Co/Ni-btc-MOF [200]. The Co/Ni-MOF was transformed into the of NiCo_2_S_4_-Ni_9_S_8_-DYMs via two-step calcinations (500 °C for 2 h under N_2_ atmosphere and another 2 h under air atmosphere) followed by sulfurization (400 °C for 3 h under N_2_ atmosphere, mass ratio of S/calcined sample = 5). Conductive carbon and heterogeneous sulfides (NiCo_2_S_4_ and Ni_9_S_8_) with faradaic reactivity effectively constructed the composite with hierarchical and porous structure. The NiCo_2_S_4_-Ni_9_S_8_-DYMs was a battery-type electrode for ASC. The ASC of NiCo_2_S_4_-Ni_9_S_8_-DYMs//rGO gel showed high maximum capacitance (143.5 F g^−1^ at 2 A g^−1^) and suitable rate performance (51.0 W h kg^−1^ at 1399.4 W kg^−1^ and 32.5 W h kg^−1^ at 8004.4 W kg^−1^) in 6 M KOH.

##### Another Kind

A novel composite of MnS/MoS_2_/C was prepared from the Mn/Mo-MOF self-sacrificing precursor, [Mn(4,4′-bpy)0.5∙MoO_4_]∙1.5H_2_O, and sulfur powder by simultaneous thermal process of calcination and sulfurization [201]. The precursor was synthesized from MnSO_4_∙4H_2_O, Na_2_MoO_4_∙2H_2_O and 4,4′-bpy under reflux at 120 °C (4 h). Then, single-step thermolysis of the precursor with the S (mass ratio of precursor/powder = 1:14) was conducted to form the MnS/MoS_2_/C composite at 300 °C (1 h, Ar). The obtained microporous composite (S_BET_ = 8.1 m^2^ g^−1^, *I*_G_/*I*_D_ = 1) was made up of MnS/MoS_2_/C nanoflakes and MoS_2_/C nanorods. The carbon matrix played a role for conducting and maintaining the structure. The active centers for pseudocapacitance, such as MnS and MoS_2_, could effectively undergo the following redox reactions under basic conditions: MnS + OH^−^ ↔ Mn(OH)S + e^−^, Mn(OH)S + OH^−^ ↔ MnOS + H_2_O + e^−^, (MoS_2_) + K⁺ + e^−^ ↔ MoS-SK⁺. In the 3E system with 2 M KOH electrolyte, the MnS/MoS_2_/C electrode delivered high capacitances of 1162 F g^−1^ at 0.5 A g^−1^ and 880 F g^−1^ at 10 A g^−1^. In the same electrolyte, the ASC composed of MnS/MoS_2_/C positive electrode and AC negative electrode (m_+_/m_-_ = 1/2.4) also demonstrated good performances: 93 F g^−1^ at 0.5 A g^−1^, 45 F g^−1^ at 10 A g^−1^, 31.0 W h kg^−1^ at 388.3 W kg^−1^, and 15.0 W h kg^−1^ at 7722.2 W kg^−1^. At 1.5 A g^−1^, the ASC showed 81% and 61% capacitance retentions after 5000 and 10,000 GCD cycles, respectively, due to the transformation of metal sulfides into hydrated polyoxometalates or metal hydroxides (for example, MoS_2_ → hydrated polymolybdates, MnS → Mn(OH)_x_).

#### 4.8.4. Carbon/Metal Hydroxide Composite

By using ZIF-67 self-sacrificing precursor, the 3D flower morphological composite of Co/C@Ni(OH)_2_ was synthesized to improve the conductivity and cycling stability of Ni(OH)_2_ electrode [202]. The ZIF-67 precursor was directly carbonized at 600 °C (3 h, N_2_ flow) to prepare Co/C core. The obtained Co/C contained microporosity and uniform hexagonal shape (V_total_ = 0.640 cm^3^ g^−1^). The Co/C core was transformed into the core/shell type of composite, i.e., Co/C@Ni(OH)_2_, via chemical deposition method using K_2_S_2_O_8_, NiSO_4_, and NH_3_∙3H_2_O at 80 °C. In the resultant composite, Co NPs tended to enhance the conductivity. The amorphous porous carbon upgraded the cycling stability, and the 2D ultrathin nanoflake shell, i.e., Ni (OH)_2_, provided high capacitance. As a result, in the 3E system with 6 M KOH electrolyte, a working electrode of the Co/C@Ni(OH)_2_ showed high capacitances of 952 F g^−1^ at 0.5 A g^−1^ and 692 F g^−1^ at 5 A g^−1^, and low charge transfer resistance (R_ct_) of 0.28 Ω. Co/C@Ni(OH)_2_//AC ASC (m_+_/m_−_ = 0.3) demonstrated moderately good performance in the same electrolyte: 73.8 F g^−1^ at 0.5 A g^−1^, 61.2 F g^−1^ at 2 A g^−1^, and 33.6 W h kg^−1^ at 516.3 W kg^−1^.

#### 4.8.5. Other Kinds

**Figure 15:**
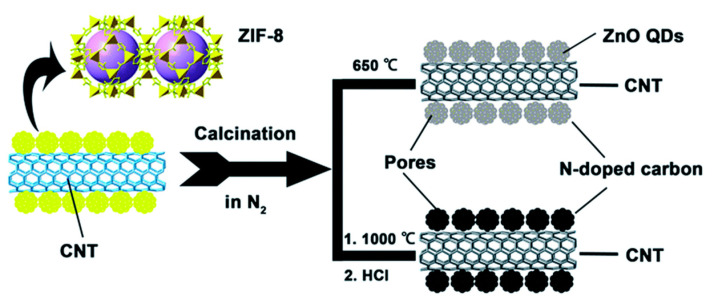
Fabrication process of single-templating approach for ZnO QDs/carbon/CNTs//N-doped carbon/CNTs ASC [203].

Lin et al. employed ZIF-8/CNTs as a sole precursor for the construction of ZnO QDs/carbon/CNTs//N-doped carbon/CNTs ASC as illustrated in Figure 15 [203]. First, the precursor of ZIF-8/CNT-*x* (*x* = CNT contents in mg) was hydrothermally synthesized from ZIF-8 and carboxyl-functionalized CNTs. Then, the positive electrode of ZnO QDs/carbon/CNTs was prepared by one-step carbonization of ZIF-8/CNT-120 at 650 °C (N_2_ flow). The resultant ZnO QDs/carbon/CNTs had micro-/mesoporosity (D_pore_ = 4.0 nm), spherical defect-free ZnO QDs, CNT, and amorphous carbon. In the 3E cell with 1 M Na_2_SO_4_, the positive electrode showed capacitances of 185 F g^−1^ at 0.5 A g^−1^, 152 F g^−1^ at 20 A g^−1^, 175 F g^−1^ at 5 mV s^−1^, and 153.6 F g^−1^ at 100 mV s^−1^. Meanwhile, the negative electrode of N-doped carbon/CNTs was obtained from direct carbonization of the ZIF-8/CNT-120 at 1000 °C (2 h, N_2_ flow) and HCl washing. The negative electrode showed micro-/mesoporosity, N-doping (~2.7 at%), and hierarchical structure. Thus, it delivered capacitances of 250 F g^−1^ at 1 A g^−1^ and 102 F g^−1^ at 20 A g^−1^. Moreover, all-solid-phase ASC of the ZnO QDs/carbon/CNTs//N-doped carbon/CNTs was assembled with a controlled mass ratio (m_+_/m_−_ = 1.35), and it operated with PVA-NaNO_3_ electrolyte. The ASC exhibited capacitances of 59 F g^−1^ at 1 A g^−1^ and 27 F g^−1^ at 20 A g^−1^. These capacitive performances were attributed to the stable architecture, abundant pores for electrochemical activity, and conducting networks.

Shao et al. introduced a single-templating approach for the flexible all-solid-phase ASC of ZnO@C@CoNi-LDH//Fe_2_O_3_@C [204]. The vertical growth of ZnO NRAs on a Ni foam substrate surface was hydrothermally achieved. After that, the ZIF-8 template was epitaxially grown on the ZnO NRAs’ surfaces. The obtained ZnO@ZIF-8 was directly carbonized to form ZnO@C at 650 °C (2 h, N_2_ flow), and the ZnO@C was converted into the ZnO@C@CoNi-LDH via electrosynthesis using CoCl_2_∙6H_2_O and Ni(NO_3_)_2_∙6H_2_O at −1.0 V. In 1 M KOH electrolytic 3E cell, the positive electrode yielded an areal capacitance of 6.2578 F cm^−2^ at 2 mA cm^−2^ and 30.4% rate performance at 50 mA cm^−2^ with the help of two redox coupling reactions (Co^2+^/Co^3+^ and Ni^2+^/Ni^3+^). Additionally, the ZnO@ZIF-8 NRAs were transformed into Fe(OH)_3_@ZIF-Fe NRAs through cation-exchange reaction followed by carbonization to produce core-shell NRAs of the Fe_2_O_3_@C at 450 °C (5 h, N_2_). Under the same cell conditions, the negative electrode showed an areal capacitance of 0.194 F cm^−2^ at 2 mA cm^−2^ with the formation of Fe(OH)_2_ and FeOOH. Furthermore, the ASC of ZnO@C@CoNi-LDH//Fe_2_O_3_@C (area = 1.0 cm^2^, thickness = 0.04 cm) was fabricated and operated in PVA/KOH electrolyte. The following reaction also occurred to enhance the electrochemical performances: 6Ni(OH)_2_ + Fe_2_O_3_ ↔ 6NiOOH + 2Fe + 3H_2_O. It delivered volumetric energy and power densities of 1.078 mW h cm^−3^ at 0.02 W cm^−3^ and 0.4 W cm^−3^ at 0.254 mW h cm^−3^. Hierarchical core/shell structures with plentiful electrochemical active sites much favorable for conductivity and stability were found in both electrodes.

The hollow composite of C/LDH/S with inter-layered hybrid system of Ni-Co-LDH/Co_9_S_8_ was derived from ZIF-67 [205]. The ZIF-67 crystals were converted into ZIF-67-C via direct carbonization at 400 °C (2 h, N_2_ flow). Subsequently, the ZIF-67-C was hydrothermally transformed into hollow C/LDH by treating Ni(NO_3_)_2_∙6H_2_O at 120 °C for 2 h. Then, the hollow C/LDH/S was obtained by hydrothermal sulfurization of the C/LDH by treating TAA at 100 °C for 2 h. The resultant C/LDH/S (V_pore_ = 0.439 cm^3^ g^−1^, D_pore_ = 49.4 Å) contained polyhedral morphology, amorphous carbon, extended interlayer spacing, and ~20 wt.% of the Co_9_S_8_. As a result, the optimal interfacing system of the C/LDH/S with the combination of conductive matrix and rich redox centers could be realized in 1 M KOH electrolyte. Thus, C/LDH/S working electrode in a 3E cell showed high capacitances of 1653 F g^−1^ at 4 A g^−1^ and 1025 F g^−1^ at 20 A g^−1^. Moreover, the ASC of C/LDH/S//CNTs (m_+_/m_-_ ≅ 8.2) delivered capacitances of 194 F g^−1^ at 4 A g^−1^ and 151 F g^−1^ at 12 A g^−1^.

## 5. Conclusions

Many attempts to synthesize porous carbons directly from MOFs have been intensively pursued for the past decade. It has been very clearly revealed that MOFs would be ideal self-sacrificing templates for the preparation of porous carbon electrodes in supercapacitors. This MOF-based carbonization may have several advantages compared to traditional carbonization method using organic-based precursors. For example, depending on the types of MOF templates and detailed conditions of carbonization, several different types of porous carbon-based electrode materials can be easily accessible on demand. Porous pure carbonaceous materials can be prepared through the carbonization of MOFs with/without subsequent acid etching process. Some volatile derivatives of metal ions can be removed without any difficulty during the high-temperature carbonization process. Sometimes, however mild acid etching is required to completely remove unwanted metal species. The heteroatom-doped porous carbons can be effectively synthesized by using a similar approach to use MOFs with bridging ligands containing heteroatoms, such as N, S, P, and B. The heteroatom dopants can also be derived from the solvate molecules confined in MOFs. Generally, O-dopant is also naturally occurring during the carbonization. Thus, multiply doped porous carbons can be prepared in a controlled manner. Metallic NP-containing carbons are obtainable through the pyrolysis of MOFs containing non-volatile metallic elements. Porous carbon composites with other carbon-based materials or redox-active metal species can be synthesized in a simple manner as well. As we noted, most of these MOF-derived porous carbons acted as good and stable electrodes of supercapacitors. Nonetheless, one of the most challenging issues using these porous carbon-based electrodes directly derived from MOFs is the preparation of porous carbon with sufficiently large surface area and optimal porosity. Many examples showed that the activation process was usually required to obtain such porous carbons. Therefore, further works may focus on the preparation of MOFs generating high-surface porous carbons with suitable pore dimensions without additional activation process. Additional electrochemical performance enhancement by redox-active species is also a critical factor for high performance supercapacitors. To increase the electrical conductivity of the composite electrodes is another interesting research topic. Overall, the composite-based electrodes showed enhanced energy and power densities compared to pure carbon electrodes. Notwithstanding, continuous efforts towards supercapacitors with better performance suited for practical applications may be needed. Despite several advantages of MOF-derived carbons as electrode materials for supercapacitors, one difficulty for commercializing these materials may be their high cost. Although there is no reliable production cost information about MOFs, MOFs are still more expensive than other organic-based precursors due to the constituent parts of MOFs. Therefore, future research may include the scale-up synthesis of MOF-derived carbons, development of cost-effective synthetic processes, and expansion of their practical uses.

## Figures and Tables

**Table 1:** The electrochemical capacitive performances of porous carbons derived from MOF-5 and other non-ZIF-based Zn-MOFs.

Samples	*S*_BET_ (m^2^ g^−1^)	*C*_max_ (F g^−1^)	*E*_max_ (W h kg^−1^)	*P*_max_ (W kg^−1^)	System	Ref.
NPC	2872	312	-	-	2E	27
NPC530	3040	158	-	-	2E	28
NPC650	1521	222	-	-	2E	28
NPC800	1141	151	-	-	2E	28
NPC900	1647	148	-	-	2E	28
NPC1000	2524	149	-	-	2E	28
MC	1812	172	23.6	-	3E/2E	29
MPC	1543	193	24.6	-	3E/2E	29
MAC	384	104	3.6	-	3E/2E	29
MC-A	1673	222	27.4	-	3E/2E	29
MPC-A	1271	196	27.6	-	3E/2E	29
MAC-A	2222	274	31.2	-	3E/2E	29
3D hierarchical porous carbon	1600	175	-	-	2E	30
MDC-D	2980	-	-	-	3E	31
IPC3-M	1515	212	-	-	2E	32
MOF-5-C 850	609	244	-	-	3E	33
MOF-5/AC-C nsp 850	677	300	-	-	3E	33
IMCS4-8-6	860	242	8.41	9907	2E	34
Porous carbon	2619	148.8	17.37	13,516.4	3E/2E	35
PVDF/CMOF-5	847	218	-	-	2E	36
Laminated HPC	2368	234	-	-	3E	37
C-MOF-5	2184	150	-	-	2E	38
C-MOF-2	1378	170	-	-	2E	38
C-Zn-BTC	1326	134	-	-	2E	38
C-Zn-NDC	920	114	-	-	2E	38
C-Zn-PAA	495	110	-	-	2E	38
C-Zn-ADA	513	95	-	-	2E	38
CIRMOF-3-950	553	239	-	-	2E	39
CIRMOF-3-800	402	153	-	-	2E	39
CIRMOF-3-700	454	54	-	-	2E	39
CIRMOF-3-600	391	0.9	-	-	2E	39
CMOF-5-950	572	24	-	-	2E	39
GNRib	1492	273	-	-	2E	40
CNRod	1559	187	-	-	2E	40
MPC	1286	120	-	-	2E	40
C-S-800	319	61	-	-	2E	41
C-S-900	1356	369	12.5	7200	2E	41
C-S-1000	1122	248	-	-	2E	41
C-B-800	191	35	-	-	2E	41
C-B-900	543	116	-	-	2E	41
C-B-1000	528	148	-	-	2E	41
BM-700	682	192	-	-	3E	42
BM-800	823	175	-	-	3E	42
BM-900	1115	188	-	-	3E	42
BM-1000	1241	179	-	-	3E	42
KBM-700	1129	230	-	-	3E	42
KBM-800	1059	225	-	-	3E	42
KBM-900	959	176	-	-	3E	42
KBM-1000	909	154	-	-	3E	42
MPC-950	1455	471	-	-	3E	43
MPC-850	746	437	-	-	3E	43
MPC-750	589	401	-	-	3E	43
MPC-650	518	377	-	-	3E	43
S800	774	418	-	-	3E	44
S800	774	73	-	-	3E	45
S900	828	318	-	-	3E	45
S1000	903	284	-	-	3E	45
CNFs	1725	280	-	-	3E	46
NPC800	562	269.3	-	-	3E	47
NPC1000	546	164.3	-	-	3E	47
ZBDh-D10-900	1490	~80	-	-	2E	48
ZBDt-M10-900	1230	~80	-	-	2E	48
HPC	1391	166	-	-	3E	87
HPCs-0	1796	185	-	-	3E	87
MC-Zn	420	121.3	-	-	3E	88
PC-Zn	1558	138	-	-	3E	89
MOF-DC	2714	-	~20	-	2E	90
CMOF-5	2489	206	-	-	3E	112
C-MOF	1117	225	-	-	3E	113

**Table 2:** The electrochemical capacitive performances of porous carbons derived from ZIF-8.

Samples	*S*_BET_ (m^2^ g^−1^)	*C*_max_ (F g^−1^)	*E*_max_ (W h kg^−1^)	*P*_max_ (W kg^−1^)	System	Ref.
C1000	3405	188	-	-	2E	49
Z-900	1075	214	-	-	3E	50
AS-ZC-800	2972	251	-	-	2E	51
NMCs	2737	307	-	-	3E	52
C-ZIF-8	745	181	-	-	3E	53
NPC	1523	251	10.86	2281	3E/2E	54
Carbon-ZS	934	285.8	-	-	3E	55
NPC	1873	21.0	~6.6 ^a^	-	2E	56
3D hybrid-porous carbon	1057	332	-	-	3E	57
S-900	1823	219	14.64	-	3E/2E	58
HPCNFs-N	418	307.2	10.96	25,000	2E	59
NPCF	315	332	-	-	3E	60
NHCF-66.7%ZIF-8	560	302	-	-	3E	61
3D-BN-CNF-ZF900	352	295	-	-	3E	62
N-NFC-8	277	387.3	7.9	-	3E/2E	63
NMCS-8	1937	232	-	-	3E	64

^a^ In mW h cm^−3^.

**Table 3:** The electrochemical capacitive performances of porous carbons derived from other ZIF-based Zn-MOFs.

Samples	*S*_BET_ (m^2^ g^−1^)	*C*_max_ (F g^−1^)	*E*_max_ (W h kg^−1^)	*P*_max_ (W kg^−1^)	System	Ref.
CZIF68	472	54	-	-	3E	65
CZIF69	586	37	-	-	3E	65
CZIF68a	1861	112	-	-	3E	65
CZIF69a	2264	168	-	-	3E	65
Carbon-L-750	455	127.6	-	-	3E	66
Carbon-L-850	686	200.72	-	-	3E	66
Carbon-L-950	783	228.1	-	-	3E	66
Carbon-L-1000	799	135.4	-	-	3E	66
Carbon-G-950	640	238	-	-	3E	66
Carbon-F-950	566	100.9	-	-	3E	66
Carbon-E-950	515	152	-	-	3E	66
Carbon-Z-950	406	202	-	-	3E	66
Z950	76	210	41.3	3600	2E	67
N-PCMPs	895	180	-	-	3E	68
N-PCMPs-A	2188	307	-	-	3E	68
UT-CNSs	1535	347	-	-	3E	69
NNPC-700	736	158	-	-	3E	70
NNPC-800	942	272	5.36	~1000	3E/2E	70
NNPC-900	1018	225	-	-	3E	70

**Table 4:** The electrochemical capacitive performances of carbons derived from other types of MOFs.

Samples	*S*_BET_ (m^2^ g^−1^)	*C*_max_ (F g^−1^)	*E*_max_ (W h kg^−1^)	*P*_max_ (W kg^−1^)	System	Ref.
MIL-C	1328	145	-	-	2E	71
MIL-C-0.5	1699	143	-	-	2E	71
MIL-C-1	2116	180	-	-	2E	71
MIL-C-2	1397	185	-	-	2E	71
CNs	415	119	4.3	2068	3E/2E	72
PC-bib	224	107	-	-	3E	73
PC-bbib	56	63	-	-	3E	73
PC-bibp	165	93	-	-	3E	73
PC-bbibp	18	48	-	-	3E	73
APC-bib	1290	164	-	-	3E	73
APC-bbib	997	121	-	-	3E	73
APC-bibp	1269	127	-	-	3E	73
APC-bbibp	622	115	-	-	3E	73
PC-me	23	32	-	-	3E	74
PC-eth	51	54	-	-	3E	74
PC-ipr	10	7	-	-	3E	74
PC-dmf	122	92	-	-	3E	74
PC-nmp	96	76	-	-	3E	74
APC-me	1143	106	-	-	3E	74
APC-eth	1312	103	-	-	3E	74
APC-ipr	1074	85	-	-	3E	74
APC-dmf	1408	156	-	-	3E	74
APC-nmp	1337	105	-	-	3E	74
NPC-800	943	238	19.6	22,900	3E/2E	75
NPC-600	286	114	-	-	3E	76
NPC-750	440	110	-	-	3E	76
NPC-900	553	149	-	-	3E	76
APC	2491	260.5	18.38	6881	3E/2E	77
C-S700	817	182	-	-	3E	78
C-S900	704	156	-	-	3E	78
C-Cl700	311	117	-	-	3E	78
C-Cl900	199	70	-	-	3E	78
HCPs	1147	214	-	-	2E	79
NPS-800	1192	1636	89.73	-	2E	80
CP-II	768	121	-	-	3E	81
CP-III	732	127	-	-	3E	81
PGC-900	1148	217	-	-	3E	82
NPGC-1-900	1116	254	-	-	3E	82
NPGC-2-900	1027	293	47.5	~30,000	3E/2E	82
NPGC-3-900	993	284	-	-	3E	82
NPGC-2-1000	579	184	-	-	3E	82
NPGC-2-800	649	167	-	-	3E	82
AC-2-900	1002	205	-	-	3E	82
GC-2-900	196	49	-	-	3E	82
N-C-450	-	42	-	-	3E	83
N-C-550	-	229.9	-	-	3E	83
N-C-650	-	223.7	-	-	3E	83
NC0.9	603	296	-	-	3E	84
NC1.1	773	426	-	-	3E	84
NC1.35	786	425	-	-	3E	84
NC2.0	534	210	-	-	3E	84
NC2.7	393	186	-	-	3E	84
UCN-10-550	367	125	-	-	3E	85
UCN-20-550	1084	210	-	-	3E	85
UCN-30-550	1034	175	-	-	3E	85
UCN-40-550	837	140	-	-	3E	85
UCN-20-650	931	165	-	-	3E	85
UCN-20-750	843	256	-	-	3E	85
UCN-20-850	426	128	-	-	3E	85
WMC	2587	344	-	-	3E	86
HPCs-0.1	2137	215	-	-	3E	87
HPCs-0.4	2857	241	-	-	3E	87
MC-Cu	50	142.3	-	-	3E	88
MC-Al	1103	232.8	-	-	3E	88
PC-Cd	1430	126	-	-	3E	89
GC-Ni	151	43	-	-	3E	89
GC-Co	118	42	-	-	3E	89
GC-Mn	232	60	-	-	3E	89
C-Cu	86	26	-	-	3E	89
CC@NC	-	321.9	-	-	3E	95
TM-NPCs	998	330	-	-	3E	97
C2-700	-	186	-	-	3E	138
C-300	145	207	-	-	3E	180
NPC	1757	272	-	-	3E	182

**Table 5:** Electrochemical performances of the ASCs based on pure and heteroatom-doped carbons.

Samples	*S*_BET_ (m^2^ g^−1^)	*C*_max_ (F g^−1^)	*E*_max_ (W h kg^−1^)	*P*_max_ (W kg^−1^)	Ref.
MOF-DC//Li_4_Ti_5_O_12_	2714//-	-	~65	~10,000	90
WO_3_/C//MOF-NC	127//1474	184.3	159.97	1736	91
CNT@NiO//PCPs	-//1980	72	25.4	16,000	92
Mn_2_O_3_@NF//MC	76//365	317.5	112.82	~2000	93
Zn-Co-O@CC//NPC@CC	90//-	210	117.92	13,520	94
Ni-Co-O@CFP//NPC@CFP	119//332	201	69	-	95
CC@Co_3_O_4_//CC@NC	-//-	~116.8	41.5	49,200	96
CC@NiCo_2_O_4_//CC@NC	12//-	~89.7	31.9	22,900	97
TM-nanorods//TM-NPCs	477//998	161	47.1	17,104	98

**Table 6:** The electrochemical capacitive performances of metallic NP-containing carbons derived from MOFs.

Samples	*S*_BET_ (m^2^ g^−1^)	*C*_max_ (F g^−1^)	*E*_max_ (W h kg^−1^)	*P*_max_ (W kg^−1^)	System	Ref.
C800MOF	140	886	25	11,250	3E	99
CNT	400	272 ^a^	0.0544 ^b^	5.988 ^c^	2E	100
HP-Ni@C-N	111	912	-	-	3E	101
CNRod700	373	67	-	-	3E	102
CNRod800	367	127	4.4	-	3E	102
CNRod900	375	98	-	-	3E	102
Ni/P/N/C-500	22	2887.87	-	-	3E	103
Ni/P/N/C-600	27	1699.3	-	-	3E	103
Ni/P/N/C-700	35	1901.9	-	-	3E	103
Ni/P/N/C-800	247	402	-	-	3E	103
Metallic cobalt/carbon	236	144.5	-	-	3E	104
Ni foam/cobalt/carbon	-	512.0	-	-	3E	105
LIMDG	440	1.36 ^a^	0.14 ^b^	580 ^c^	2E	106
Co(CO_2_)_2_Pz 700 AL	320	330	9.1	7000	3E/2E	107
Co(CO_2_)_2_Pz 900 AL	394	430	-	-	3E	107
CNS-800	770	156	-	-	2E	108
NC-800	84	1069	-	-	3E	109
Ni-Co@carbon	238	236	-	-	3E	110
Co@carbon	-	50	-	-	3E	110
Nanoporous carbon	350	272	7.1	-	3E/2E	111
Ni/N-doped PC-500	112	2002.6	-	-	3E	112
GC	496	119	-	-	3E	141
Au@NC800	954	171.7	-	-	3E	170
Co@Carbon	110	109	1.83	3500	3E/2E	182
Co/C	216	146	-	-	3E	200

^a^ In mF cm^−2^. ^b^ In mW h cm^−2^. ^c^ In mW cm^−2^.

**Table 7:** The electrochemical capacitive properties of Zn-MOF-derived carbon/carbon composites.

Samples	*S*_BET_ (m^2^ g^−1^)	*C*_max_ (F g^−1^)	*E*_max_ (W h kg^−1^)	*P*_max_ (W kg^−1^)	System	Ref.
rGO/CMOF-5	2040	312	17.2	5200	3E/2E	113
C-GMOF	979	345	30.3	11,900	3E/2E	114
C-GZ-2	353	238	-	-	2E	115
NPC/G	703	235	-	-	3E	116
GNPC	-	144	-	-	3E	117
HPNCs/rGO-800	710	298	18.0	12,000	3E/2E	118
ZIF-8/GO (1ǀ700)	1201	181	-	-	3E	119
ZIF-8/GO (1ǀ800)	1304	246	-	-	3E	119
ZIF-8/GO (1ǀ900)	1303	81	-	-	3E	119
NCGs-600	204	143	-	-	3E	120
NCGs-700	773	174	-	-	3E	120
NCGs-800	816	225	12.7	15,126	3E/2E	120
ZC@G-40	1091	77	-	-	2E	121
HC-40-4	2837	206	87.5	43,750	2E	121
NC/rGO-1	400	188	-	-	2E	122
NC/rGO-2	489	280	19.45	~10,000	2E	122
NC/rGO-3	290	184	-	-	2E	122
C(ZIF8)@GQDs	668	159.6	-	-	3E	123
hCNT/PCP	898	~130	-	-	2E	124
C-ZIF-8@MWCNT	569	326	-	-	3E	125
CNTs/NCP	898	308	12.0	~7000	3E/2E	126
MWCNT/NPC-S	643	198.6	-	-	3E	127
MWCNT/NPC-M	885	229	-	-	3E	127
MWCNT/NPC-L	928	302.2	12.65	2257.2	3E/2E	127
CNCF-5/1	468	243	-	-	3E	128
CNCF-10/1	645	340	26.6	5000	3E/2E	128
CNCF-15/1	600	270	-	-	3E	128
CNT@CZIF-1	264	168	-	-	3E	129
CNT@CZIF-2	287	324	-	-	3E	129
CTAs@NCBs-700(T)	-	244	-	-	3E	130
ZM-C-800	558	376.2	16.4	4985	3E/2E	131
g-CN/NCPPs0.1	454	495	11.89	-	3E/2E	132
GCNP-800	920	426	59.40	4560	3E/2E	133
PC1000@C	1116	225	-	-	3E	134
NHCSF-1	585	198.5	-	-	3E	135
NHCSF-2	679	208.2	-	-	3E	135
NHCSF-3	816	253.6	13.33	~8000	3E/2E	135
NHCSF-4	667	219.5	-	-	3E	135
N-GQD@cZIF-8/CNT	520	541	18.75	2175	3E/2E	136
PMC	-	180.4	-	-	3E	177
AQ-NPCs	268	373	-	-	3E	180
TN-NPCs	356	392	-	-	3E	180
N-doped carbon/CNTs	600	250	-	-	3E	201

**Table 8:** The electrochemical capacitive properties of other MOF-derived carbon/carbon composites.

Samples	*S*_BET_ (m^2^ g^−1^)	*C*_max_ (F g^−1^)	*E*_max_ (W h kg^−1^)	*P*_max_ (W kg^−1^)	System	Ref.
C/NG-A	814	421	33.89	25,000	3E/2E	137
HPCF1	330	177.4	-	-	3E	138
HPCF2	449	249.4	-	-	3E	138
HPCF3	525	356.1	-	-	3E	138
HPCF4	620	381.2	~10	~5000	3E/2E	138
HPCF5	569	323.7	-	-	3E	138
C-600	1094	252	-	-	3E	139
C-700	1563	302	-	-	3E	139
C-800	1446	265	-	-	3E	139
C-900	1354	223	-	-	3E	139
GA@CZIF-67-E	207	53	-	-	2E	140
NC@GC(0.05)	1276	270	-	-	3E	141
NC@GC(0.15)	-	255	-	-	3E	141
NC@GC(0.35)	813	149	-	-	3E	141
NC@GC(0.5)	-	136	-	-	3E	141
Co/Zn NPC	415	286	-	-	3E	142
HZ-NPC	298	171	-	-	3E	174
CNT@NC	-	278.8	-	-	3E	179

**Table 9:** The electrochemical capacitive properties of carbon/metal oxide composites.

Samples	*S*_BET_ (m^2^ g^−1^)	*C*_max_ (F g^−1^)	*E*_max_ (W h kg^−1^)	*P*_max_ (W kg^−1^)	System	Ref.
CeO_2_@C(RT)	384	895	-	-	3E	143
CeO_2_@C(ST)	53	1102	-	-	3E	143
Co_3_O_4_/C NAs	-	776.5	8.54	-	3E/2E	144
Co_3_O_4_/NC-90-15	74	1.22 ^a^	-	-	3E	145
NC600	310	57	-	-	3E	146
NC600AT	764	98	-	-	3E	146
NC900	115	88	-	-	3E	146
NC900AT	272	67	-	-	3E	146
Cu-Cu_2_O-CuO/C 700	-	782	-	-	3E	147
Cu-Cu_2_O-CuO/C 800	-	773	-	-	3E	147
Fe_3_O_4_/carbon	38	162	-	-	3E	148
MOFC-300	-	232	-	-	3E	149
MOFC-400	-	520	-	-	3E	149
MOFC-500	-	109	-	-	3E	149
MOFC-600	72	972	-	-	3E	149
MOFC-700	-	381	-	-	3E	149
NC/Mn_3_O_4_-1	-	136	-	-	3E	150
MoO_2_@Cu@C	183	28.56 ^b^	2.58	790.38	3E/2E	151
RuO_2_/PCs-1	-	444.4	-	-	3E	152
RuO_2_/PCs-2	-	481.4	-	-	3E	152
RuO_2_/PCs-3	198	539.6	23.38	12,000	3E/2E	152
RuO_2_/PCs-4	-	493.3	-	-	3E	152
ZnO/C	140	394	-	-	3E	153
(Co_0.94_Fe_0.06_)_3_O_4_@CON	-	161	-	-	3E	154
ZMCN	143	589	-	-	3E	155
BMM-9-800	-	159.4	-	-	3E	156
BMM-9-900	-	263.2	-	-	3E	156
BMM-9-1000	-	159.5	-	-	3E	156
M/MO@C-700	242	894	-	-	3E	157
ZnO@C@NiCo_2_O_4_ NRSA	-	2650	-	-	3E	158
Co_3_O_4_/C	273	793	-	-	3E	172
CuO_x_@mC700	-	93.3	-	-	3E	178
C/CoO-200	5	1052	-	-	3E	181
Co_3_O_4_@Carbon	24	261	0.97	6000	3E/2E	182
MOXC-700	181	600	-	-	3E	183
S-α-Fe_2_O_3_@C	117	1538	-	-	3E	184
MnO_x_-CSs-600	182	220	-	-	3E	186
MnO_x_-CSs-800	61	130	-	-	3E	186
MNCMn-60	906	163	-	-	3E	187
Mn_2_O_3_/C	22	776	-	-	3E	188
NiCo_2_O_4_-NC	126	310	-	-	3E	191
Cr_2_O_3_/C	60	426	-	-	3E	192
Fe_x_O_y_/C	68	114	-	-	3E	192
ZnO QDs/carbon	-	85.4	-	-	3E	201

^a^ In F cm^−2^. ^b^ In mA h g^−1^.

**Table 10:** The electrochemical capacitive properties of other types of composites.

Samples	*S*_BET_ (m^2^ g^−1^)	*C*_max_ (F g^−1^)	*E*_max_ (W h kg^−1^)	*P*_max_ (W kg^−1^)	System	Ref.
CoSNC	-	360.1	-	-	3E	159
(*o*)-CoS_2_@CNT	28	155	-	-	3E	160
(*m*)-CoS_2_@CNT	8	422	-	-	3E	160
(*p*)-CoS_2_@CNT	18	839	-	-	3E	160
Co_9_S_8_@SNCC	212	429	-	-	3E	161
Co_9_S_8_@SNCB	88	320	-	-	3E	161
Cu_1.96_S/C-650	140	200	-	-	3E	162
Cu_7_S_4_/C	-	321.9	-	-	3E	163
PCM-900	448	99.0	13.7	-	3E	164
MoS_2_@MPC	-	189	-	-	3E	165
Cu_2_S@C	62	42	-	-	3E	166
Co_9_S_8_@C	202	227	-	-	3E	166
NiS_2_@C	349	833	-	-	3E	166
ZMP-0.04	-	500	-	-	3E	167
S3	-	1100	21	-	3E/2E	168
3CPC	-	755	-	-	3E	169
NC800-PEDOT	1186	217.7	-	-	3E	170
ZIF-8-C@NiAl LDH	383	1403	-	-	3E	171
Co_3_O_4_/C@MoS_2_-10	-	902	-	-	3E	172
Co_3_O_4_/C@MoS_2_-20	257	1096	-	-	3E	172
Co_3_O_4_/C@MoS_2_-30	-	713	-	-	3E	172
Ni(OH)_2_-MnO_2_/C	-	862.0	22.1	8500.0	3E	173
HZ-NPFC/250-2	223	323	-	-	3E	174
HZ-NPFC/250-5	202	545	-	-	3E	174
HKUST-1@TS-800	-	1812 ^a^	-	-	3E	175
MCG-1	-	212	-	-	3E	176
MCG-2	326	456	-	-	3E	176
MCG-3	-	301	-	-	3E	176
PMCP	-	468.6	-	-	3E	177
CuO_x_@mC300@PANI	-	381.6	-	-	3E	178
CuO_x_@mC500@PANI	-	413.2	-	-	3E	178
CuO_x_@mC700@PANI	-	509.5	-	-	3E	178
CuO_x_@mC900@PANI	-	319.8	-	-	3E	178
CuO_x_@mC700@PANI@rGO	-	569.4	-	-	3E	178
CoNi@SNC	224	1970	-	-	3E	193
Co_9_S_8_/NS-C-1.5 h	66	734.09	25.49	2840.9	3E	194
Co_9_S_8_@C-500	63	1887	-	-	3E	195
Co_9_S_8_@C-600	37	1672	-	-	3E	195
Ni-Co-S@G	43	1463	-	-	3E	196
CC/CNWAs@Ni@CoNi_2_S_4_	-	3163	-	-	3E	198
MnS/MoS_2_/C	8	1162	-	-	3E	199
MnS/MoS_2_/MoO_3_/C	-	495	-	-	3E	199
Ni(OH)_2_@Co/C	-	952	-	-	3E	200
ZnO QDs/carbon/CNTs	435	185	-	-	3E	201
C/LDH/S	116	1653	-	-	3E	203

^a^ In mF cm^−2^.

**Table 11:** Electrochemical performances of the asymmetric supercapacitors (ASCs) based on carbon composites.

Samples	*S*_BET_ (m^2^ g^−1^)	*C*_max_ (F g^−1^)	*E*_max_ (W h kg^−1^)	*P*_max_ (W kg^−1^)	Ref.
CNT@NiCo-LDH//CNT@NC	-//-	119.8	37.4	-	179
TN-NPCs//AQ-NPCs	356//268	86	23.5	~20,000	180
C/CoO-200//C-300	5//145	92	25.04	7000	181
Co@Carbon//Co_3_O_4_@Carbon	110//24	28.2	8.8	3000	182
AC//Co-ZIF-450	-//-	4 ^a^	1.32 ^b^	376 ^c^	183
MOXC-700//NPC	181//1757	170	17.496	-	184
S-α-Fe_2_O_3_@C//Na-MnO_2_ NSs	117//-	201.3 ^d^	135.3 ^e^	21,998.4 ^f^	185
CF@NiCo-A-S//Fe_x_O_y_@CNS	-//-	16.8 ^g^	48.2	8300.0	186
MnO_x_-CSs-600//AC	182//-	61.1	27.5	5400	187
MNCMn60//MNC950	906//920	-	76.02	22,000	188
AC//Mn_2_O_3_/C	-//22	166	54.9	22,680	189
MnO_2_@C-NS//NPCS	45//1292	~124	166	3900	190
NQD-NC//AC	268//-	-	76.9	11,250	191
NiCo_2_O_4_-NC//NC	126//1823	89	28	8500	192
Cr_2_O_3_/C//Fe_x_O_y_/C	60//68	27.2	9.6	8000	193
CoNi@SNC//AC	224//-	156.7	55.7	-	194
Co_9_S_8_/NS-C-1.5 h//AC	66//-	75.59	14.85	6818.18	195
Co_9_S_8_@C-500//AC	63//-	166	58	17,200	196
Ni-Co-S@G//AC	43//-	217.8	51.0	11,700	197
Ni-Co-S-0.5/NC//AC	10//-	111.2	39.6	7910	198
CC/CNWAs@Ni@CoNi_2_S_4_//AC	-//-	151.3	53.8	-	199
NiCo_2_S_4_-Ni_9_S_8_-C DYMs//rGO gel	62//-	143.5	51.0	8004.4	200
MnS/MoS_2_/C//AC	8//-	93	31.0	7722.2	201
Ni(OH)_2_@Co/C//AC	-//-	73.8	33.6	~2000	202
ZnO QDs/carbon/CNTs//N-doped carbon/CNTs	435//600	59	23.6	16,900	203
ZnO@C@CoNi-LDH//Fe_2_O_3_@C	-//-	-	1.078 ^b^	0.4 ^h^	204
C/LDH/S//CNTs	116//-	194	39	7400	205

^a^ In F cm^−3^. ^b^ In mW h cm^−3^. ^c^ In mW cm^−3^. ^d^ In mF cm^−2^. ^e^ In μW h cm^−2^. ^f^ In μW cm^−2^. ^g^ In mA h g^−1^. ^h^ In W cm^−3^.
